# Supplementary material for: Assessing the relationship between agency and peer violence among adolescents aged 10 to 14 years in Kinshasa, Democratic Republic of Congo and Blantyre, Malawi: A cross-sectional study
Source: PLoS Med. 2021 Dec 13;18(12):e1003552. doi: 10.1371/journal.pmed.1003552 (PMC8716028; doi:10.1371/journal.pmed.1003552)
Supplement: S1 IRB — DRC, Democratic Republic of Congo; IRB, Institutional Review Board. (DOCX) [file pmed.1003552.s002.docx]

**JHSPH IRB Research Plan for New Data Collection**

**PI Name:** Robert Blum

**Study Title:** Global Early Adolescent Study – Phase 2 (Kinshasa)

**IRB No.:**

**PI Version No./Date:** V1/November 14, 2016

**I. Aims of the Study:**

The Global Early Adolescent Study (GEAS) is the first international study exploring how gender norms evolve over time and inform a spectrum of adolescent health outcomes, including sexual and mental health, through the adolescent years. The first phase, consisting of formative research and the face validity and pilot testing of instruments among early adolescents 10-14 years of age across 15 countries, is nearing completion. IRB oversight for all instrument development was provided for the first phase under IRB #00005684. The present research plan is in reference to the second, longitudinal phase of the GEAS. This phase, like the first, will be conducted in multiple international sites. However, because the longitudinal phase will likely be paired with different interventions or approaches in the partner sites, protocol details will vary and thus IRB approval will be sought for each site separately. The present application is for conducting Phase 2 of the Global Early Adolescent Study in Kinshasa, Democratic Republic of the Congo (DRC).

The objective of Phase 1 of the GEAS was to develop a theoretically derived, cross-culturally validated set of instruments appropriate for use with early adolescents in urban poor settings. With pilot testing data collection coming to a close, Phase 1 has resulted in a set of survey instruments that measure perceptions of gender norms, attitudes toward gender equitability, school retention, empowerment, healthy sexuality, physical and mental health, sexual health, exposure to gender-based violence, and other ecological factors influencing adolescent health such as family and peer relationships, parental connectedness, and neighborhood cohesion.

This longitudinal phase builds on the first by exploring how gender norms relate to health across the adolescent years, beginning with early adolescence. The GEAS in Kinshasa has two sets of objectives: 1) To explore how perceptions of gender norms evolve across adolescence, the factors influencing these changes, and how perceptions of gender norms predict a spectrum of adolescent outcomes, and 2) Assess the impact of a gender norms transformative intervention developed and implemented by Save the Children. The intervention, Growing Up GREAT (GUG), and evaluation components are part of a larger project, *Passages*, which is led by the Institute for Reproductive Health (IRH) at Georgetown University. Our research partner is the Kinshasa School of Public Health (KSPH), which will implement the GEAS study. The Kinshasa component of the project has both USAID and Gates Foundation support.

In both an intervention and control group 1,400 young people ages 10-14 will be followed over a period of 4 years, with one additional year of data analysis. To gauge effectiveness of the intervention, we will assess the following measurable primary and secondary study outcomes:

Primary outcomes:

- Doubling of contraception use among sexually active youth
- 10% decrease in reports of exposure to gender-based violence (GBV) compared with the previous 6 months

Secondary outcome:

- 20% shift toward more gender-equitable attitudes and beliefs

**II. Background and Rationale:**

While its expressions may vary, gender inequality is pervasive and persistent across time and place. There is growing recognition that gender norms significantly shape sexual behaviors and contribute to negative sexual and reproductive health outcomes among adolescents including sexual coercion, sexually transmitted infections, and early pregnancies. The qualitative research during the Phase 1 of the GEAS strongly suggests that patterns of gender norms structuring male and female behaviors reinforce gender unequal power dynamics during early adolescence. This process operates across numerous cultures and contributes towards maintaining patriarchal systems. The evidence suggests that such normative beliefs predispose to youth to subsequent gender-based violence, early school leaving, depression, and a host of negative sexual health outcomes, yet research lacks the measurement and longitudinal data to establish the temporality and strength of these associations. The GEAS is set to answer these questions using a longitudinal design and validated measures to explore these processes over time and across cultures.

Since initiating Phase 1 of the Global Early Adolescent Study nearly 4 years ago, awareness has grown globally that there is a need to focus on this age group both to understand the processes by which gender norms are reinforced and for the development of interventions that shift toward more gender equitable relationships.

The focus of the GEAS is on urban poor youth, a rapidly increasing population.

**III. Study Design**

**Overview of study design and methods.** The GEAS in Kinshasa will involve a quasi-experimental longitudinal (pretest-midline-posttest) design to compare changes in perceptions of gender norms and subsequent effects on adolescent health outcomes between a group of early adolescents receiving Save the Children’s GUG intervention and a non-intervention control group. The control group is an essential feature of the study and will allow the exploration of developmental changes in adolescent health in the absence of an intervention during this critical period of human development.

The selection of intervention and control groups, which will include a sample of both in-school youth (ISY) and out-of-school youth (OOSY), will be based on geography, school selection (the ISY samples), and self-selection of adolescents participating in the intervention activities, described in detail in Section IV, “Participants” below. All participants will complete a baseline survey (before the intervention begins) and will be surveyed every 6 months over a period of 5 years. The survey will be divided into 2 segments, each collected 6 months apart in order to collect a complete wave of data every year. Additionally, this approach will minimize respondent burden and minimize loss to follow-up due to frequency of contact. Starting with the second wave, the survey instrument will be modified to include questions appropriate for older adolescents, and at that time a supplemental IRB application/amendment will be submitted. Following the same procedure as in the Phase 1 pilot, data collection will be conducted by trained interviewers using mobile tablets with the capacity for face-to-face interview also known as CAPI (computer-assisted personal interview), computer-assisted self-interview (CASI), and/or ACASI (audio computer-assisted self-interview) survey administration. When CAPI is the primary administration method, CASI/ACASI will be used for potentially uncomfortable questions, such as those about adverse childhood experiences and sexual and reproductive health, in order to increase privacy and improve data quality. We will continue to use the secure survey design and data collection software (SurveyCTO) used during the Phase 1 pilot test, which greatly improves data security over other methods.

**IV. Participants**

The survey will take place beginning with 10-14-year-olds and their parents or caretakers (at baseline) in two urban poor communes in Kinshasa: Kimbanseke and Masina. What follows is a detailed description of study site selection, followed by descriptions of selection of parent and adolescent participants in both the intervention and control arms of the study.

*Study sites*

The survey will take place in two urban poor communes of the city of Kinshasa: Kimbanseke (population 946,000) and Masina (population 485,000), home to large populations of urban poor dwellers. Each of these communes is divided into neighborhoods (quartiers); Masina has a total of 21 quartiers, Kimbanseke has 46.

A mapping exercise conducted by Save the Children in May 2016 identified a total of 1,266 primary and secondary schools serving nearly 325,000 children located in the two communes. There are three main types of schools in Kinshasa: 1) public schools without religious affiliation, 2) public schools with religious affiliation, and 3) private/ religious schools. While each type of school attracts the lowest income populations, there is income diversity among the three types of schools, and more poorer children attend public schools compared with private. In consultation with KSPH researchers, Save the Children identified intervention and control neighborhoods in each commune based on equal representation of public schools and geographical divisions allowing a separation between intervention and control neighborhoods so as to minimize contamination.

*In-school sample*

Because a portion of the GUG intervention in-class and after-school activities, the selection of in-school participants will use a multistage cluster sampling approach to select at the first stage schools (clusters) based on geographical location and school type, and, subsequently, at the second stage, select adolescents from participating schools.

We will perform a random selection of schools in both the intervention zone and the control zone after stratification by commune and by school type, with unequal probabilities of selection based on school size. The school type strata are defined as follows:

1. Public non-religious schools
2. Public religiously-affiliated schools
3. Private religious schools

Table 1. Number of schools and enrollment

|  | **Masina** | | **Kimbanseke** | |
| --- | --- | --- | --- | --- |
|  | *Intervention*  *N=* | *Control*  *N=* | *Intervention*  *N=* | *Control*  *N=* |
| **Public, non-religious** | 9 | 5 | 16 | 1 |
| **Public, religious** | 20 | 14 | 24 | 41 |
| **Private** | 37 | 51 | 26 | 42 |

*In-school adolescent participants*

For both the intervention and control arms of the study, students will be invited to participate until 25 from each school assent (and whom have obtained parental consent). We will apply this predetermined cluster size to select the same number of students (n=25) per school in the intervention and control areas. Students in the control schools will be selected at random (from a list of all students between 10 and 14 years in the school), while those in the intervention area are self-selected based on their choice to participate in the intervention.

A total of 40 schools per study arm, equally distributed between Masina and Kimbanseke, will be selected. Altogether, 1,000 students will be enrolled in each school arm (intervention and control). Sample size calculation is detailed in the section, “Sample size and justification.”

*Parents of in-school adolescents*

All parents of in-school participants who consent their children to participate will be asked to participate in a baseline survey collecting sociodemographic information about themselves and household information.

*Out-of-school sample*

*Out-of-school adolescent participants*

Out-of-school participants will be recruited with the assistance of community-based organizations (CBOs) and religious organizations that work with OOSY in these communes. Specifically, KSPH will work with CBOs that serve out of school youth in each quartier and group (control or intervention) to create a list of eligible youth from which participants will be recruited. Youth whom have been out of school for 2 or more years will be prioritized due to the decreased likelihood that they will re-enroll in school during the study compared with youth who have been out of school for less time. A total of 400 out-of-school adolescents will be recruited in each arm of the GEAS cohort.

*Parents of out of-school adolescents*

All parents of out of-school participants will be asked to participate in a baseline survey collecting sociodemographic information about themselves and household information.

*Inclusion and exclusion criteria*

At the beginning of the study, youth aged 10-14 will be enrolled. In Kinshasa, it is not uncommon for a youth to live with a family other than their own either in hopes of broadening horizons or due to family dissolution or orphan status; and thus, youth living in foster families will be eligible to participate. However, where parents are available their consent will be sought. Where participants are orphaned, foster parent or guardian consent will be obtained. Homeless youth will not be eligible to participate due to the likelihood of difficulty with follow-up as well as involvement in intervention programming.

Table 2. Inclusion and exclusion criteria

|  | **Inclusion Criteria** | **Exclusion Criteria** |
| --- | --- | --- |
| **In-school youth** | Males and females between the ages of 10-14 years of age | Not between the ages of 10-14 |
|  | Lives within the geographic boundaries of Kimbanseke or Masina | Does not live within the geographic boundaries of Kimbanseke or Masina |
|  | Lives at home with a family (biological, adoptive, or foster) | Is homeless or lives on the street |
|  | Attends a school selected for the study | Attends a school not selected for the study, or does not attend school |
|  | Able to assent | Unable to assent |
|  | Has obtained informed consent from a parent or guardian to participate in the study | Has not obtained informed consent from a parent or guardian to participate in the study |
| **Parent/guardian of in-school youth** | Parent or guardian of an eligible in-school adolescent who has assented to participate | Not a parent or guardian of a participating in-school adolescent |
|  | Has provided informed consent to participate | Has not provided informed consent to participate |
| **Out-of-school youth** | Males and females between the ages of 10-14 years of age | Not between the ages of 10-14 |
|  | Lives within the geographic boundaries of Kimbanseke or Masina | Does not live within the geographic boundaries of Kimbanseke or Masina |
|  | Lives at home with a family (biological, adoptive, or foster) | Is homeless or lives on the street |
|  | Has not been enrolled in school for 6 or more months | Has not been enrolled in school for fewer than 6 months or is enrolled in school |
|  | Able to assent | Unable to assent |
|  | Has obtained informed consent from a parent or guardian to participate in the study | Has not obtained informed consent from a parent or guardian to participate in the study |
| **Parent/guardian of out-of-school youth** | Parent or guardian of an eligible out-of-school adolescent who has assented to participate | Not a parent or guardian of a participating out-of-school adolescent |
|  | Has provided informed consent to participate | Has not provided informed consent to participate |

**Sample size and justification**

In each arm of the study 1,400 youth will be recruited for a total of 2,800 participants. The intervention arm will include 1,000 ISY and 400 OOSY, and the control arm will also include 1,000 ISY and 400 OOSY.

The sample size calculation is based on the frequency of intervention endpoints, the effect size of the intervention, the cluster design and related design effect, as well as expected cohort attrition rate all calculated in the school sample.

We assume a cluster size of 25, an intra-class correlation of 0.15 and 20% attrition per cluster.

The power calculation for contraception is calculated among the girl sample (cluster of 10 girls per school accounting for the 20% attrition). Baseline contraceptive prevalence in Kinshasa among 15-17 year old adolescents is 13%, based on recent PMA2020 data collected in 2016. A cluster sample of 500 adolescents per group (40 clusters) will detect a doubling of contraceptive prevalence in the intervention arm with a power of 79%. The pregnancy rate of 5% based on PMA2020 estimate is too low to be able to detect a difference between intervention and control groups in this study.

Calculations were done using the Stata clustersampsi command for the two primary outcomes:

- Contraception: clustersampsi, binomial samplesize p1(0.13) p2(0.26) k(80) rho(0.2) alpha(0.05) beta(0.8)
- GBV: clustersampsi, binomial samplesize p1(0.10) p2(0.05) k(40) rho(0.02) alpha(0.05) beta(0.8)

All other indicators including attitudinal and knowledge measures are based on continuous scores. Calculations based on preliminary results from the gender norms scales from the pilot GEAS in Kinshasa indicate that a sample size of 800 adolescents (40 clusters of 20 adolescents each, after attrition) is sufficient to detect a 10% shift in the gender scores between the intervention and control groups.

Table 3. Sample size calculation

| **Endpoints** | *Control* | ***Effect size*** | *Intra-class correlation* | ***Power*** | ***N clusters /arm*** | ***Minimum average students/ cluster*** | ***N participants / arm*** |
| --- | --- | --- | --- | --- | --- | --- | --- |
| **Attitudes** |  |  |  |  |  |  |  |
| Stereotypical gender norms (continuous score 13 to 62) | *Scale 1 Mean=3.99*  *SD=0.59*  *Scale 2*  *Mean=3.55*  *SD=0.69* | *Scale 1 detectable different =0.14*  *Scale 2 detectable different =0.17* | *0.15* | *80%* | *40* | *20* | *800* |
| Discussed contraception | *6%* | *RR=2.5* | *0.20* | *76%* | *40* | *20* | *800* |
| **Behavioral** |  |  |  |  |  |  |  |
| Proportion using modern contraception | *13%* | *RR=2* | *0.2* | *79%* | *40* | *11* | *440 girls* |
| Pregnancy | *5%* | *RR=0.5* |  | *27%* | *40* | *20* |  |
| Exposure to gender based violence in the last 6 Months (dichotomous) | *10%* | *RR=0.5* | *0.2* | *0.98* | *40* | *20* | *560* |

**V. Study Procedures**

The role of Hopkins researchers in the present application is primarily development, design, oversight and coordination of the research.

*Training of interviewers*

During Phase 1 a 1-day training-of-trainers session was held in June 2015 that included the site-PI (Prof. Kayembe) and field coordinator (Dr. Mafuta) that focused on the implementation of data collection platform using tablets equipped with ODK software. The KSPH team is well versed to mobile technology platform data collection, as they have pioneered this type of platform through the PMA2020 project in Kinshasa over the last 3 years. In addition, Prof. Kayembe and Dr. Mafuta have completed the CITI Hopkins training for human subjects research and have active certificates.

KSPH successfully adapted and used GEAS training materials provided by Hopkins to train 20 interviewers over 3 days for the pilot conducted in February 2016. With the help of the Hopkins team, KSPH will adapt the training material for Phase 2 of the GEAS. The 5-day training will include the following:

- Research instruments
- Adolescence from a developmental perspective
- Consent and assent process: principles and role playing
- Identifying, managing and reporting case of child abuse
- Confidentiality and privacy
- The art of interviewing
- Data collection and transmission using ODK software on tablets

1. Recruitment Process:

**In-school participants**

As described in Section IV, “Participants,” above, our intervention partners at Save the Children recently conducted a mapping exercise with guidance from KSPH researchers, the results of which have aided in our determination of recruitment sites.

Prior to study recruitment, the KSPH research team will inform the ministry of education, the ministry of health, and the national council for adolescent health “PNSA” (Plan National pour la Santé des Adolescents) about the schools and community organizations participating in the GEAS evaluation. The ministry of education will provide a signed letter for school directors acknowledging that the school has been selected to participate in the GEAS study.

- Control participants

Trained research interviewers hired by KSPH will visit each selected school in the control area to explain the research to them, and obtain their approval for their students to participate in the study. Working with the school directors, the KSPH team will establish a list of eligible students (10-14 years of age) attending the school in order to draw a random sample of 25 adolescents per school with replacement to participate in the longitudinal study. The school director will then organize a short meeting at the end of class with the eligible students to indicate that they have been selected for a study on gender and health in their community and that their parents will be informed about the study and invited for an information session at the school. The director will indicate to the students that their teachers will inform them about the time and place of data collection. Formal permission from school directors and community organizations (after-school programs, scout organizations, sports clubs for youth) will be obtained if data collection takes place in their facilities.

Next, the school-parent committee (PTSA) will send a letter to parents of selected students informing them that their child has been selected for participation in the study and indicating that interviewers from KSPH will be hosting an information session with a given date and time at the school. The parent information session will describe the study goals and procedures, and the risks and benefits of participating. In addition, they will be explicitly informed of their child’s confidentiality and the procedures in place in the event of disclosure of abuse. Parents will be asked at the end of the session if they consent for their child to participate and if they themselves consent to participate in the baseline questionnaire. If parents give consent to both, they will be asked to complete the short (15 minute) questionnaire, either by themselves (CASI) if they can read and navigate a tablet on their own, or with the help of an interviewer (CAPI).

After parents have provided consent for their children’s participation, adolescents will be informed and reminded by their teachers regarding the time and place of data collection and whether it will take place in the school facilities or in a communal area (church or community building) near the school, depending on the availability school facilities. All adolescents from the same school will be convened at the same time. Before data collection, the adolescents will be collectively informed about the study goals, procedures, and the consent procedure including the protocol for child abuse disclosure. At the end of the information session, children will have an individual conversation with his/her interviewer in order to assent to participate. After assent has been given, data collection will proceed.

- Intervention participants

Save the Children employs locally based intervention experts whom will take charge of recruitment of adolescents participating in the intervention by approaching schools within our target communes of Masina and Kimbanseke. Save the Children will establish a list of children participating in the intervention, and, of these, 25 will be randomly selected for participation in the study (if the intervention includes more than 25 students). Save the Children will facilitate the introduction of the KSPH team who will visit the school director of each selected school in the intervention arm to explain the GEAS research and obtain approval for their students to participate in the longitudinal study. School directors will provide the KSPH team with a list of all adolescents aged 10-14 years in the school in order to assess the potential selection bias of voluntary participation in the intervention (criteria for being in the intervention arm). Formal permission from school directors and community organizations (after-school programs, scout organizations, sports clubs for youth) will be obtained if data collection takes place in their facilities.

The process for recruitment of in-school intervention participants follows the same as that for in-school control participants, outlined above. Specifically, the school director will organize a short meeting at the end of class with the eligible students to indicate that they have been selected for a study on gender and health in their community and that their parents will be informed about the study and invited for an information session at the school. The director will indicate to the students that their teachers will inform them about the time and place of data collection.

Next, the school-parent committee (PTSA) will send a letter to parents of selected students informing that their child has been selected for participation in the study and indicating that interviewers from KSPH will be hosting an information session with a given date and time at the school. The parent information session will describe the study goals and procedures, and the risks and benefits of participating. In addition, they will be explicitly informed of their child’s confidentiality and the procedures in place in the event of disclosure of abuse. Parents will be asked at the end of the session if they consent for their child to participate and if they themselves consent to participate in the baseline questionnaire. If parents give consent to both, they will be asked to complete the short (15 minute) questionnaire, either by themselves (CASI) if they can read and navigate a tablet on their own, or with the help of an interviewer (CAPI).

After parents have provided consent for their children’s participation, adolescents will be informed and reminded by their teachers regarding the time and place of data collection and whether it will take place in the school facilities or in a communal area (church or community building) near the school, depending on the availability school facilities. All adolescents from the same school will be convened at the same time. Before data collection, the adolescents will be collectively informed about the study goals, procedures, and the consent procedure including the protocol for child abuse disclosure. At the end of the information session, children will have an individual conversation with his/her interviewer in order to assent to participate. After assent has been given, data collection will proceed.

**Out-of-school participants**

- Control participants

KSPH will call upon standing relationships with community relays whom assisted in recruitment of participants in Phase 1 of the GEAS, along with staff from CBOs assisting with the recruitment of OOSY for the intervention, to create a list of eligible OOSY from which KSPH will recruit participants. As previously noted, youth who have been out of school for 2 or more years will be prioritized, then separating girls and boys and then sampling by age 200 adolescents from Masina and 200 from Kimbanseke.

After the sampling, KSPH will work with alongside RECOPE (Reseau Comunitaire de Protection de l'Enfant, child protection organization) members, who will facilitate house calls for KSPH researchers to meet with the parents of eligible adolescents. At those meetings, parents will be informed of the study goals and procedures and the risks and benefits of participating. In addition, they will be explicitly informed of their child’s confidentiality and of the procedures in place in the event of disclosure of abuse. At the end of the meeting, parents will be asked if they consent for their child to participate and if they themselves consent to participate in the baseline questionnaire. If parents give consent to both, they will be asked to complete the short (15 minute) questionnaire, either by themselves (CASI) if they can read and navigate a tablet on their own, or with the help of an interviewer (CAPI).

After parents have provided consent for their children’s participation, an appointment will be made for the adolescents will be informed and reminded by RECOPE members and other engaged community leaders regarding the time and place of data collection and its location (in a communal area such as a church or community building), which will be near their homes. A group of adolescents from the quartier will be convened, and the adolescents will be collectively informed about the study goals, procedures, and the consent procedure including the protocol for child abuse disclosure. At the end of the information session, children will have an individual conversation with his/her interviewer in order to assent to participate. After assent has been given, data collection will proceed.

Participants will be given as much privacy as possible, and data collection will occur in unobtrusive times/settings (i.e. participants will not miss class or be singled out), but it cannot be guaranteed that their involvement in the study will be private. Adolescent participants and their parents will be made aware of this fact at the time of consent/assent, as well as of the fact that they are free to leave the study at any time without penalty.

- Intervention participants

Save the Children will work with CBOs that serve OOSY in each quartier or section of the commune to recruit youth for involvement in the intervention and will create a list of eligible OOSY from which KSPH will recruit participants. As previously noted, youth who have been out of school for 2 or more years will be prioritized. These organizations will facilitate the distribution of letters informing parents of eligible adolescents about the study goals. RECOPE members will facilitate meetings between parents of eligible youth and researchers from KSPH. At those meetings, parents will be informed of the study goals and procedures and the risks and benefits of participating. In addition, they will be explicitly informed of their child’s confidentiality and the procedures in place in the event of disclosure of abuse. Parents will be asked at the end of the session if they consent for their child to participate and if they themselves consent to participate in the baseline questionnaire. If parents give consent to both, they will be asked to complete the short (15 minute) questionnaire, either by themselves (CASI) if they can read and navigate a tablet on their own, or with the help of an interviewer (CAPI).

After parents have provided consent for their children’s participation, adolescents will be informed and reminded by RECOPE members and other engaged community leaders regarding the time and place of data collection and its location (in a communal area such as a church or community building), which will be near their homes. A group of adolescents from the quartier will be convened, and the adolescents will be collectively informed about the study goals, procedures, and the consent procedure including the protocol for child abuse disclosure. At the end of the information session, children will have an individual conversation with his/her interviewer in order to assent to participate. After assent has been given, data collection will proceed.

Intervention activities will be available and open to youth enrolled in the study and also whom are not. That is, participants enrolled in the intervention group will be encouraged to participate, but they will do so alongside peers who may not be enrolled for any reason.

Regarding participation in the survey itself, participants will be given as much privacy as possible, and data collection will occur in unobtrusive times/settings (i.e. participants will not miss class or be singled out), but it cannot be guaranteed that their involvement in the study will be private. Adolescent participants and their parents will be made aware of this fact at the time of consent/assent, as well as of the fact that they are free to leave the study at any time without penalty.

1. Consent Process:

After parents have been notified of the study as described in Section A above, KSPH researchers will be responsible for obtaining parental consent and youth assent. These researchers are trained in research ethics and best practices, and will have been CITI certified in advance of recruitment, consent, and baseline data collection.

With the assistance of school directors and PTSAs, KSPH researchers will organize informational meetings for parents of eligible ISY at which consent for participation will be sought. With the assistance of youth-serving CBOs familiar with community members and other community representatives, KSPH data collectors will visit the homes of OOSY to inform parents of eligible adolescents about the study goals, procedures, risks and benefits and the opportunity to participate. Both parents and adolescents will be aware before the time of consent of whether the adolescent participant would be in the intervention or control arm of the study.

The researcher will obtain written informed consent from the parent for the adolescent’s participation and their own participation; or, in the event that they are unable to sign, they may make their mark. The parent will be provided a copy of the informed consent form to keep with contact information of the study staff for reference. The researcher or community liaison will serve as a witness to the consent process and will counter-sign the forms to indicate that they properly obtained consent from the parent. The same process will be conducted to obtain assent from the adolescent prior to baseline data collection.

Given the longitudinal nature of the study and the fact that it is confidential but not anonymous, special procedures will be undertaken so as to assure that data are secure and that participants can be assured of absolute confidentiality (see Section VI below for procedures). Additionally, since sensitive data will be collected including data indicating potential exposure to gender-based violence and abuse, participants will be advised in advance that reporting of certain events may result in a report to authorized child protection services.

Thus, during the consent process we will be explicit with both potential participants and their parents about what kind of information will be collected. Specifically, participants will be assured that information will be confidential and will not be shared except in cases of exposure to sexual or physical abuse that requires notification to authorities. In such cases data collection will cease immediately, and, with the permission of the adolescent participant (when the event involves those other than parents and immediate family), parents will be informed and the data collector will notify his or her filed coordinator who in turn notifies the site-PI (Prof. P. Kayembe), who will then prepare the report to the appropriate organization and/or agency. At that point the Hopkins study PI (R. Blum) will also be notified and in turn he will notify the JHBSPH IRB in accordance with JHU guidelines. When the events involve a parent or close family member, notification will go to a pre-selected organization tasked with protection of children and minors, who will pursue the investigation and the remainder of the reporting process will remain unchanged.

**Parent consent for adolescent participation and own participation:**

Parents will receive a letter containing information about the study from their child’s school (for ISY) or from a youth-serving CBO (for OOSY). Next, for ISY participants, interviewers from KSPH will host a parent information session at the school that will describe the study goals and procedures and the risks and benefits of participating. In addition, they will be explicitly informed of their child’s confidentiality and the procedures in place in the event of disclosure of abuse. Specifically, parents will be told that their child’s ongoing exposure to physical and/or sexual abuse will require further investigation and potential disclosure to legal and/or child protection services. They will also be informed that participation is voluntary, and their child can end participation in the study at any time with no penalty.

In order for a child to participate, his or her parent must agree to participate in a brief baseline survey module for the collection of sociodemographic data about themselves and their family. The purpose of this is to increase accuracy in reporting and decrease burden on the adolescent participant. The researcher obtaining consent will describe this portion of the study at the time of obtaining consent for adolescent participation and will describe the risks and benefits of participation.

If parents agree for their child to participate and to participate themselves, the researcher will obtain written informed consent from the parent; or, in the event that they are unable to sign, they may make their mark. The parent will be provided a copy of the informed consent form to keep with contact information of the study staff including the field coordinator, the site-PI, and the JHBSPH study coordinator (Lydia Animosa) for reference. The researcher or community liaison will serve as a witness to the consent process and will counter-sign the forms to indicate that they properly obtained consent from the parent.

**Adolescent assent:** After obtaining parental consent, KSPH researchers will visit participating schools (for ISY) or community centers (OOSY) to collectively explain the purpose of the study to adolescents whose parent provided consent for their participation and will describe the risks and benefits of being in the study, what type of information will be collected, and which types will be confidential and which, if reported, will not (e.g., participant report of physical/sexual abuse). They will also be informed that they are free to choose if they want to participate and that they can discontinue participation at any time with no penalty. Adolescents will be provided an assent form that contains the same information provided to parent including possible risks and benefits of the study, a description of the kind of information asked, and which kinds of information are confidential and which are not. Specifically, adolescent assent will occur in a private conversation with an interviewer who will tell the adolescent will be told, “If you indicate that you have been hurt or harmed in any way, we will need to stop the interview and I will need to let my supervisor know so that we can be sure you are not in ongoing danger.” If the adolescent agrees to participate, the researcher will obtain written informed consent; or, in the event that they are unable to sign, they may make their mark. The adolescent will be provided a copy of the assent form to keep with contact information of the study staff including the field coordinator, the site-PI, and the JHBSPH study coordinator (Lydia Animosa) for reference. The researcher or community liaison will serve as a witness to the assent process and will counter-sign the forms to indicate that they properly obtained assent from the adolescent.

2. Identify the countries where the research will take place, and the languages that will be used for the consent process.

| **Country** | **Consent Document(s)**  **(adult consent, parental permission, youth assent, etc.)** | **Languages** |
| --- | --- | --- |
| DRC | Adolescent assent | French & Lingala |
| DRC | Parental consent for child participation & own participation | French & Lingala |

C. Study Implementation:

Answer the following:

1. Describe the procedures that participants will undergo. If complex, insert a table below to help the reviewer navigate.

Participants in the intervention arm will take part in an intervention program (GUG), which was found to produce positive outcomes in Uganda and Nepal and has been adapted for use in Kinshasa by Save the Children. Save the Children will hire and train staff from youth-centered CBOs to implement out-of-school components and will train teachers on how to use the in-school components. Parents of youth in the intervention arm will be invited to take part in one component. Participants will be engaged in the intervention will be in place for one school year.

Participants in both the intervention and control arms will take part in a baseline survey and will be surveyed again every 6 months over a period of 4 years.

Parents in both study arms will take part in a baseline questionnaire.

2. Describe the number and type of study visits and/or contacts between the study team and the participant, how long they will last, and where/how they will take place.

Follow-up surveys will be given every 6 months over a period of 4 years. While the baseline survey may take up to 1.5 hours to complete, follow-up surveys will last not more than 45 minutes. In-school adolescents will be surveyed at school or a nearby community center, and out-of-school adolescents will be surveyed at a local community center. The survey will primarily be interviewer-facilitated, but CASI/ACASI will be used for certain sections for increased privacy, as noted above.

All parents of participating youth will complete a short questionnaire with an interviewer at the time of consent; these interviews will be conducted in either French or Lingala as the participant prefers and last not more than 15 minutes.

Our partners at KSPH work closely with community liaisons who know participants, their families, and their locations. Parental contact information will be securely stored and used to contact parents of participants who have moved. If necessary and possible, the survey may be conducted by phone. Otherwise, the participant will be among those lost-to-follow up.

3. Describe the expected duration of the study from the perspective of the individual participant and duration overall.

Five years overall, with the participant involved for 4 years of data collection.

4. Provide a brief data analysis plan and a description of variables to be derived.

Initially at baseline descriptive analyses will be done to test for comparability of intervention and comparison groups. Subsequently bivariate statistics (Chi-Square and Student’s t-test) will be run exploring the relationships between independent variables and key outcomes. Finally multivariate analyses will be performed to evaluate associations between gender norms, empowerment and the primary outcomes of interest sexual behaviors and outcomes, gender based violence and mental health. Over time, tests of differences will be run exploring change in gender equitable attitudes, gender norms and how these changes predict changes in prevalence of gender based violence and contraceptive utilization between intervention and control groups.

5. Describe whether you are collecting or storing personal identifiers, and if yes, why you need them, and when and how you plan to dispose of them. Signatures on consent forms are considered to be identifiers.

Participants will be assigned a unique identifier, which will be recorded with their responses during electronic data collection. A separate document containing participant’s photograph, identification number, name, age, and location will be kept in a locked file cabinet at KSPH for data collectors to contact participants for follow-up. These will be retained with consent and assent forms in a dedicated and locked cabinet that will only accessed by the site PI and the study coordinator. All electronic data will be encrypted. All documents with contact information will be destroyed immediately following the last wave of data collection and only coded data without any identifiers will be retained.

6. Answer the following **if they are relevant to your study design**

**N/A**

a. If the study has different arms, explain the process for assigning participants (intervention/control, case/control), including the sequence and timing of the assignment.

b. If human biospecimens (blood, urine, saliva, etc.) will be collected, provide details about who will collect the specimen, the volume (ml) and frequency of collection, how the specimen will be used, stored, identified, and disposed of when the study is over. If specimens will be collected for use in future research (beyond this study), complete the Biospecimen Repository section below.

c. If genetic/genomic analyses are planned, address whether the data will be contributed to a GWAS or other large dataset. Address returning unanticipated incidental genetic findings to study participants.

d. If clinical or laboratory work will be performed at JHU/JHH, provide the JH Biosafety Registration Number.

e. If you will perform investigational or standard diagnostic laboratory tests using human samples or data, clarify whether the tests are validated and/or the lab is certified (for example is CLIA certified in the U.S.). Explain the failure rate and under what circumstances you will repeat a test. For all human testing (biomedical, psychological, educational, etc.), clarify your plans for reporting test results to participants and/or to their families or clinicians. Address returning unanticipated incidental findings to study participants.

f. If your study involves medical, pharmaceutical or other therapeutic intervention, provide the following information:

- Will the study staff be blind to participant intervention status?
- Will participants receive standard care or have current therapy stopped?
- Will you use a placebo or non-treatment group, and is that justifiable?
- Explain when you may remove a participant from the study.
- What happens to participants on study intervention when the study ends?
- Describe the process for referring participants to care outside the study, if needed.

**VI. Data Custody, Security, and Confidentiality Protections**

A. Data Storage

| 1. Hard Copies of Data Collection Forms. | |
| --- | --- |
| X | This activity will not involve receiving and/or accessing hard copies of data |
|  | Data collection forms RECORD NO PERSONAL IDENTIFIERS connecting study participants, and there are no codes providing a link. Data are anonymous. |
|  | Data collection forms INCLUDE IDENTIFIERS. The forms are locked in a secure cabinet or room with limited access by authorized individuals. Forms will be kept in study team’s possession during transport and will not be left unattended in a vehicle. When possible, de-identified copies will be used for coding and analysis. |
| X | Data collection forms ARE CODED with study participants’ random study ID numbers. Codes/links between study IDs and identifiers are stored securely in a separate place (locked storage cabinet or secure electronic database.) |
|  | Other: |
| 2. Electronic Data | |
| X | The data do not contain personally identifiable information |
| X | These data are stored on a secure server protected by limited access and strong password systems. Data are coded when possible. Portable electronic devices will not contain identifiable information unless encrypted. |
|  | Other: |
| 3. Other Identifiable Data Storage, Retention, and Destruction (Audiotapes, videotapes, photographs, etc.) will be retained and stored securely (locked in cabinet or room) until: | |
|  | Transcription is complete, then will be destroyed. |
|  | Analysis is complete, then will be destroyed. |
|  | Study is complete and file is closed. |
|  | Indefinitely. Provide justification for indefinite retention: |
| 4. Existing Biospecimens to be used in this study: | |
|  | HAVE NO PERSONAL IDENTIFIERS. |
|  | INCLUDE IDENTIFIERS AND ARE CODED; the PI will not have access to the link or code connecting the identifiers to the specimens. |
|  | INCLUDE IDENTIFIERS, and the PI has access to those identifiers or to the link/code connecting specimens to individuals. The identifiers and/or code will be stored securely until the study is complete. |

B. Certificate of Confidentiality

Will the study data stored in the United States be protected by a Certificate of Confidentiality? If yes, explain who will apply for and maintain the Certificate. (<http://grants.nih.gov/grants/policy/coc/appl_extramural.htm>)

No.

C. Data Security and Sharing

Study data will be collected using encrypted mobile tablets, and completed surveys will be uploaded to a secure server immediately upon completion using mobile hotspots or SIM cards. Servers are maintained by SurveyCTO, a consulting firm that manages large scale survey data studies which is under contract with the GEAS. No data will be stored on the tablets. During the course of the study no identification information will be shared except with data collectors for the explicit purpose of contacting participants for the subsequent wave of data collection. Once coded, electronic data will be shared with the Hopkins study team for purposes of data analysis and report generation. Once the three waves of data collection are complete and data are linked then all electronic data will be de-identified and thus anonymous; reporting will be in the aggregate.

**VII. Risks of the Study**

A. Describe the risks, discomforts, and inconveniences associated with the study and its procedures, including physical, psychological, emotional, social, legal, or economic risks, and the risk of a breach of confidentiality. These risks should be described in the consent documents.

B. Describe the anticipated frequency and severity of the harms associated with the risks identified above; for example, if you are performing “x” test/assessment, or dispensing “y” drug, how often do you expect an “anticipated” adverse reaction to occur in a study participant, and how severe do you expect that reaction to be?

C. Describe steps to be taken to minimize risks. Include a description of your efforts to arrange for care or referral for participants who may need it.

D. Describe the research burden for participants, including time, inconvenience, out-of pocket costs, etc.

E. Describe how participant privacy will be protected during data collection if sensitive questions are included in interviews.

This study involves a low level of risk since this is survey research; however, some of the questions may cause discomfort or a desire for self-disclosure of adverse experiences or exposures. To assure that any issues that arise are addressed in a timely fashion we will collaborate with both Save the Children and Georgetown University/IRH, both of whom have ongoing relationships with youth-serving and child protection agencies in Kinshasa. The protection of vulnerable youth is detailed below (p. 17).

As noted above, some information collected as part of the GEAS is personal, and participants will be informed that this information is confidential and that they have the right to refuse answering questions or sections of the survey without prejudice. Participants will be asked questions about a range of topics including family, school, sexual attitudes and behaviors, mental health, and adverse childhood experiences any of which may make them uncomfortable. It should be noted that the modules on adverse childhood experiences and on sexual and reproductive health will be completed using CASI/ACASI rather than by interviewer so that the data are directly entered by the adolescent into the tablet. As noted previously, data collectors will undergo a 5-day training prior to data collection to learn among other things how to recognize if participants are feeling significant distress. Data collectors will be informed that if an adolescent participant experiences signs of distress, they will immediately remind the participant that they do not need to answer any questions that make them feel uncomfortable. If the respondent decides to stop, the data collector will notify the field coordinator who will notify the respondent’s parent/guardian of the situation. If he/she asks to continue the data collector will do so; however, if discomfort is expressed a second time the data collector will cease data collection and will notify the field coordinator immediately. Additionally, the young person and parent/guardian will be provided resources for help if needed.

In all cases, data collectors will be required to record if a participant experiences distress and inform the field coordinator who will advise the site PI. Where there is suspicion or evidence of physical or sexual abuse the data collector will be instructed to stop the interview, inform the subject of his/her concern and notify the field coordinator and site PI. If the site PI determines that the situation warrants follow up he will contact the designated child protection agency and concurrently notify the Hopkins PI who in turn will notify the JHBSPH IRB detailing the steps being taken to protect the child. Where the abuse may involve a parent or family member the report will go directly to the field coordinator/PI however if it does not, the parent will also be informed. Additionally, a detailed child protection protocol specific to Kinshasa is in place.

1. Describe any potential direct benefits the study offers to participants (“payment” for participation is not a direct personal benefit).

Those in the intervention arm of the study will benefit from an evidence-based program that has been developed and implemented by Save the Children. Previous research suggests that the intervention is effective in increasing gender equitability and shifting gender norm attitudes that benefit participants. We hypothesize that those who will be participants will also be improved contraceptive users and less likely to be perpetrators or victims of gender-based violence.

There is no direct benefit to participants in the control group beyond what has been previously reported as the benefits research subjects feel in being able to share their experiences, exposures and perspectives.

Participants in both groups will be provided a meal and a t-shirt with the study logo.

1. Describe potential societal benefits likely to derive from the research, including value of knowledge learned.

The evidence is that gender-based violence is endemic in the DRC and the direct hypothesized benefit is that the intervention will reduce GBV by 20% and double effective contraception. Additionally, we hypothesize that there will be secondary benefits of more gender equitable relationships between boys and girls in the intervention group, improved mental health outcomes and an increase in healthy sexuality and school retention.

**IX. Payment:**

1. Describe the form, amount, and schedule of payment to participants. Reimbursement for travel or other expenses is not “payment,” and if the study will reimburse, explain.

While there are no monetary payments to be provided to either parents or adolescents, at the times of data collection in schools and at centers, food will be prepared and made available for both adolescent participants and parents. Additionally, all participants in the intervention and control arms will be provided a t-shirt with the study logo.

1. Include the possible total remuneration and any consequences for not completing all phases of the research.

N/A

**X. Study Management**

A. Oversight Plan:

1. Describe how the study will be managed.

The study will be under the oversight of faculty at the Johns Hopkins Bloomberg School of Public Health. Specifically, Robert Blum and Caroline Moreau will serve as the study directors with Lydia Animosa as the Hopkins based project coordinator (for issues of reporting untoward events and other correspondence with the IRB, Robert Blum will be the point of contact). Dr. Moreau is francophone and will have responsibility for training of the entire research team and for on-going contact with the field coordinator, Eric Mafuta. As this is a sub-contract with the Georgetown University Institute for Reproductive Health and since the implementing partner, Save the Children, is also a subcontractor, bi-weekly teleconferences assure coordination across these three groups (Robert Blum and Lydia Animosa represent JHBSPH on those calls). The Kinshasa School of Public Health serves as a sub-contractor under JHBSPH for the research; and there are regular coordinating calls between these two groups (Caroline Moreau represents JHBSPH on those calls).

2. What are the qualifications of study personnel managing the project?

Robert Blum, MD, PhD, MPH is the William H. Gates Sr. Professor and Chair of the Department of Population, Family and Reproductive Health. He currently is the PI of the Global Early Adolescent Study (Phase 1) and the impact evaluation of the UNICEF-IKEA Adolescent Project in Afghanistan. He has nearly 40 years experience of research in adolescent health.

Caroline Moreau, MD, PhD is the William Robertson Associate Professor in the Department of Population, Family and Reproductive Health. She is the co-PI of the Global Early Adolescent Study (Phase 1) and has led a number of national sexual and reproductive health surveys in France and is a technical advisor for PMA2020—a monitoring and evaluation program using mobile technology to track family planning indicators in a number of francophone settings including Kinshasa.

Lydia Animosa, MSPH is the study coordinator of the Global Early Adolescent Study (Phase 1) and has served in that capacity since June 2015.

- How will personnel involved with the data collection and analysis be trained in human subjects research protections? (Use the JHSPH Ethics Field Training Guide on our website.)

See Section V above, which outlines the components of the 5-day training and describes human subject research training and certification.

- If the PI will not personally be on-site throughout the data collection process, provide details about PI site visits, the supervision over consent and data collection, and the communication plan between the PI and study team.

The on-site study team will be under the direction of Patrick Kayembe, MD, PhD Professor, Kinshasa School of Public Health and field coordinator Eric Mafuta, MD, PhD. Dr. Moreau will have monthly calls with the Kinshasa research team and in addition she will be in regular email communications. She will be present and will supervise the training of data collectors and either she or Blum will be on site in Kinshasa at least once every 6 months to meet with the research team. Annual refresher trainings will be held.

B. Recordkeeping:

Describe how you plan to ensure that the study team follows the protocol and properly records and stores study data collection forms, IRB regulatory correspondence, and other study documentation. For assistance, contact [housecall@jhsph.edu](mailto:housecall@jhsph.edu).

As has been noted previously, both identification information and consent/assent forms will be stored at KSPH under locked cabinet and direct control of the site PI. No other hard copy data will be retained. The electronic data collection system allows for both quality checks of data and algorithms that allow for oversight of data collectors to assure that data are not fabricated.

C. Safety Monitoring

1. Describe how participant safety will be monitored as the study progresses, by whom, and how often. Will there be a medical monitor on site? If yes, who will serve in that role?

As noted in the study protocol, the GEAS is based on self-report of experiences and perceptions. First line of participant safety monitoring will be the responsibility of the data collector who will be trained to have a high index of suspicion for discomfort and distress. The data collector will be backstopped in turn by the field coordinator and the site PI. They will have access to a counselor from a child protection agency for issues, concerns and consultation. As the study PI, Dr. Blum will have overall responsibility for study participant safety.

2. If a Data Safety Monitoring Board (DSMB), or equivalent will be established, describe the following:

a. The DSMB membership, affiliation and expertise.

b. The charge or charter to the DSMB.

c. Plans for providing DSMB reports to the IRB.

N/A

3. Describe plans for interim analysis and stopping rules, if any.

N/A

D**.** Reporting unanticipated problems/adverse events (AE’s) to the IRB (***all studies must complete this section***):

Describe your plan for reporting to the IRB and (if applicable) to the sponsor. Include your plan for government-mandated reporting of abuse or illegal activity.

As noted previously, the protocol of the study requires that if the data collector suspects that the participant is distressed then the data collector will ask the study participant if the observation is correct. If no, then the data collection will continue. If yes, then the participant will be asked if she/he wants to continue. If no, then the data collection will cease, the participant will be provided information on where she/he might seek help and the data collector will notify the field coordinator. If the data collection continues and if in the opinion of the data collector distress continues then the data collection will cease and the same procedure as noted above will be followed. If there is suspected abuse then the protocol will be followed as described in Section VII.

Whenever a report of suspected child abuse is made, the site PI will notify the study PI (R. Blum) who in turn will notify the JHBSPH IRB as well as the University Counsel both of the event and subsequently of the disposition.

NOTE: The IRB does not require submission for all AEs, only those that are **unanticipated, pose risk of harm to participants or others, and are related to the study**.

E. Other IRBs/Ethics Review Boards:

If other IRBs will review the research, provide the name and contact information for each IRB/ethics review board and its Federal Wide Assurance, if it has one (available on OHRP’s website at <http://www.hhs.gov/ohrp/assurances>).

This study will be reviewed and approved by the Kinshasa School of Public Health Institutional Review Board.

F. Collaborations with non-JHSPH Institutions:

For studies that involve collaboration with non-JHSPH institutions, complete the chart below by describing the collaboration and the roles and responsibilities of each partner, including the JHSPH investigator. This information helps us determine what IRB oversight is required for each party. Complete the chart for all multi-collaborator studies.

**Insert Name of Institutions in Partner column(s); add additional columns if necessary.**

|  | **JHBSPH** | **IRH** | **KSPH** |
| --- | --- | --- | --- |
| **Primary grant recipient** |  | X |  |
| **Oversight, coordination, and leadership** | X |  |  |
| **On site coordination and data collection** |  |  | X |

**For the following, indicate “P” for “Primary”, “S” for “Secondary” as appropriate to role and level of responsibility.) Add additional items if useful.**

|  |  | **JHBSPH** | **IRH** | **KSPH** |
| --- | --- | --- | --- | --- |
| 1 | Human subjects research ethics training for data collectors | P |  | S |
| 2 | Day to day management and supervision of data collection | S |  | P |
| 3 | Reporting unanticipated problems to the JHSPH IRB/Sponsor | P |  | S |
| 4 | Hiring/supervising people obtaining informed consent and/or collecting data | S |  | P |
| 5 | Execution of plan for data security/protection of participant data confidentiality, as described in Sect. 5. | S |  | P |
| 6 | Biospecimen processing, storage, management, access, and/or making decisions about future use | N/A |  | N/A |

**COMPLETE THE FOLLOWING SECTIONS WHEN RELEVANT TO YOUR STUDY:**

**XI. Secondary Data Analysis of Existing Data:** N/A

A. Study Design

1. Describe your study design and methods. The study design must relate to your stated aims/objectives.

2. Provide an estimated sample size and an explanation for that number.

3. Provide a brief data analysis plan and a description of variables to be derived.

B. Participants

1. Describe the subjects who provided the original data and the population from which they were drawn.

2. Describe whether the data contain personal identifiers of the individuals from whom the data originated. If yes, explain why you need them, and when and how you plan to dispose of them.

3. If you are receiving, accessing, or using data from a U.S. health care provider, the need for HIPAA review is likely. If you plan to bring identifiable health information from a foreign country to a U.S. covered entity (e.g., lab at the Hopkins SOM), HIPAA may be triggered. If either of these conditions is met, check “yes” to the HIPAA question in the PHIRST application.

4. If you plan to analyze human specimens or genetic/genomic data, provide details about the source of those specimens and whether they were collected using an informed consent document. If yes, explain whether your proposed use is “consistent with” the scope of the original consent, if it potentially introduces new analyses beyond the scope of the original consent, and/or if it introduces new sensitive topics (HIV/STDs, mental health, addiction) or cultural/community issues that may be controversial.

5. Explain whether (and how) you plan to return results to the participants either individually or as a group.

C. Data Management: N/A

Describe any additional plans beyond those described in Section VI that you have for storing and sharing the study data and/or materials, and how responsibility for the data will be managed.

The use of clinical data from Johns Hopkins Hospital and its affiliates requires a security review by Johns Hopkins Medicine. If you seek to access a dataset of 500 clinical records or larger, complete the *JHM Data Security Checklist* on the IRB Website.

**XII. Oversight plan for student-initiated studies:** N/A

A. For student-initiated studies, explain how the PI will monitor the student’s adherence to the IRB-approved research plan, such as communication frequency and form, training, reporting requirements, and anticipated time frame for the research. Describe who will have direct oversight of the student for international studies if the PI will not personally be located at the study site, with that person’s qualifications.

B. What is the data custody plan for student-initiated research? (Note: Students may not take identifiable information with them when they leave the institution.)

**XIII. Creation of a biospecimen repository:** N/A

Explain the source of the biospecimens, if not described above, what kinds of specimens will be retained over time. Clarify whether the specimens will be obtained specifically for repository purposes, or will be obtained as part of the core study and then retained in a repository.

- 1. Describe where the biospecimens will be stored and who will be responsible for them.
  2. Describe how long the biospecimens will be stored, and what will happen at the end of that period.
  3. Explain whether the biospecimens will be shared with other investigators, inside and outside of JHU, how the decision to share will be made, and by whom. Include the policy on commercial use and secondary distribution. Also explain how downstream use of the specimen will be managed, and what will happen to left-over specimens.
  4. Describe whether future research using the biospecimens will include specimen derivation and processing (cell lines, DNA/RNA, etc.), genomic analyses, or any other work which could increase risk to participants. Explain what additional protections will be provided to participants.
  5. If future research could yield unanticipated incidental findings (e.g., an unexpected finding with potential health importance that is not one of the aims of the study) for a participant, do you intend to disclose those findings to the study participant? Please explain your position.
  6. Explain whether the specimens will be identifiable, and if so, how they will be coded, who will have access to the code, and whether the biospecimens will be shared in linked (identifiable) form.
  7. Explain whether the repository will have Certificate of Confidentiality protections.
  8. Explain whether a participant will be able to withdraw consent to use a biospecimen, and how the repository will handle a consent withdrawal request.
  9. Describe data and/or specimen use agreements that will be required of users. Provide a copy of any usage agreement that you plan to execute with investigators who obtain biospecimens from you.

**XIV. Data Coordinating Center:**

Complete if JHSPH serves as the Data Coordinating Center.

1. How will the study procedures be developed?

The PI (Robert Blum) and co-PI (Caroline Moreau) will meet (at least) once a month through teleconference with the study on-site team members to assure adherence to the research plan. Monthly meetings will be held through an online conferencing system and regular correspondence will be maintained using email. All current study team members are trained in human subject research protections and additional research assistants who will be hired to assist in data collection and analysis will begin their employment by completing the online human subjects training. All study team members will participate in the data collection. In addition, the current study team members will conduct training for all additional hires on collecting quantitative data. We will also be hiring students who have prior experience collecting data in the community.

1. How will the study documents that require IRB approval at each local site be developed? Will there be some sort of steering or equivalent committee that will provide central review and approval of study documents, or will template consent forms, recruitment materials, data collection forms, etc. be developed by and provided to the local sites by the coordinating center without external review?

The present IRB application will be submitted in French to the Kinshasa School of Public Health IRB. If in their judgment, amendments is necessary, an addendum will be submitted to the JHBSPH IRB.

1. Will each local clinical site have its own IRB with an FWA? State whether the coordinating center will collect IRB approvals and renewals from the clinical centers or not; if not, explain why not.

N/A

1. How will the coordinating center provide each local site with the most recent version of the protocol and other study documents? What will be the process for requesting that these updates be approved by local clinical center IRBs?

There is only a single site (Kinshasa) with multiple intervention and comparison sites. All will be under the supervision and data collection of the Kinshasa School of Public Health. There will be one IRB approval for the in-country partner.

1. What is the plan for collecting data, managing the data, and protecting the data at the coordinating center?

At the Johns Hopkins Coordinating Center, a Data Quality Monitor will oversee all data collected at the collaborating site to assure that all data are collected according to protocol.

Data are recorded using tablets eliminating data coding and entry, thereby obviating human error. Data will be uploaded daily from data collectors to a central data repository at the Johns Hopkins Bloomberg School of Public health where all data files will be maintained in a secure server and dedicated computer system. Computer checks for gross inconsistencies will alert investigators to potential irregularities. Data will be kept on a dedicated, password protected computer accessible only by the core Hopkins study team and their designees.

1. What is the process for reporting and evaluating protocol events and deviations from the local sites? Who has overall responsibility for overseeing subject safety: the investigators at the recruitment site, Coordinating Center, the Steering Committee, or a data and safety monitoring board (DSMB)? Is there a DSMB that will evaluate these reports and provide summaries of safety information to all the reviewing IRBs, including the coordinating center IRB? Please note that if there is a DSMB for the overall study, then the coordinating center PI does not have to report to the coordinating center IRB each individual adverse event/problem event that is submitted by the local site PIs.

While the site PI has in-country responsibility for assuring adherence to all IRB and study protocols, the overall PI on this study, Robert Blum, will have final responsibility to assure that adherence is maintained.

1. Who is responsible for compliance with the study protocol and procedures and how will the compliance of the local sites be monitored and reviewed? How will issues with compliance be remedied?

The overall PI (Robert Blum) will have final responsibility for adherence with study protocols. This will be accomplished by translation and back translation of instruments. Discussions with data collectors and routine conference calls with site PI and coordinator.

**XV. Drug Products, Vitamins, Food and Dietary Supplements: N/A**

Complete this section if your study involves a drug, botanical, food, dietary supplement or other product that will be applied, inhaled, ingested or otherwise absorbed by the study participants. If you will be administering drugs, please upload the product information.

N/A

A. List the name(s) of the study product(s), and the manufacturer/source of each product.

| **Name of study product** | **Manufacturer/Source** |
| --- | --- |
|  |  |
|  |  |
|  |  |

B. List each study product by name and indicate its approved/not approved status.

| **Approved by the FDA and Commercially Available** | **Approved by Another Gov’t Entity (provide name)** | **Cleared for Use at Local Study Site** |
| --- | --- | --- |
|  |  |  |
|  |  |  |
|  |  |  |

C. If your study product has an Investigational New Drug (IND) application through the U.S. Food and Drug Administration, provide the IND number and attach the Investigators Brochure and the Drug Data Sheet available on the IRB website.

D. If your study product is a marketed drug, provide the package inserts or other product information. If the study product WILL NOT be used for its approved indication, dose, population, and route of administration, provide a detailed rationale justifying the off label use of the study product.

E. If the study product is not an FDA approved drug, and is being used without an IND (e.g., dietary supplements, botanicals, etc.), provide safety information (as applicable) and a certificate of analysis.

F. Explain who will be responsible for drug management and supply, labeling, dispensing, documentation and recordkeeping,

G. What drug monitoring and/or regulatory oversight will be provided as part of the study?

**XVI. Investigational Medical Devices**

Complete this section if your study will involve an investigational medical device (diagnostic, non-significant risk, significant risk).

N/A

A. List the name(s) of the study product(s), the manufacturer/source of each product, and whether or not it is powered (electric, battery). Provide product information. If it is electric, upload documentation of clinical engineering approval.

| **Name of study product** | **Manufacturer/Source** | **Powered?** |
| --- | --- | --- |
|  |  |  |
|  |  |  |
|  |  |  |

B. List each study product by name and indicate it’s approved/not approved status.

| **Approved by the FDA and Commercially Available** | **Approved by Another Gov’t Entity (provide name and approval information)** | **Not Approved** |
| --- | --- | --- |
|  |  |  |
|  |  |  |
|  |  |  |

C. If the investigational device is a Significant Risk Device, provide the IDE number given by the FDA, or if not under FDA jurisdiction, explain why it is appropriate to use this device in this study.

D. If you believe the investigational device is not IDE exempt under 21CFR 812.2(c), but is a “Non-Significant Risk” device considered to have an approved IDE application, provide information from the manufacturer supporting that position.

E. If your investigational device is Exempt from the FDA IDE regulations, explain which section of the code applies to your device and why it meets the criteria provided. If it is a diagnostic device, provide pre-clinical information about the sensitivity and specificity of the test and the anticipated failure rate. If you plan to provide the results to participants or their physicians, justify doing so.

**APPENDICES**

1. Adolescent survey instrument
2. Parent questionnaire
3. Informational letter
4. Parent consent form for the adolescent child’s participation *and* for her/his own participation
5. Adolescent assent form
6. Data collector training schedule
7. Child protection protocol
