# Supplementary material for: Assessing the relationship between agency and peer violence among adolescents aged 10 to 14 years in Kinshasa, Democratic Republic of Congo and Blantyre, Malawi: A cross-sectional study
Source: PLoS Med. 2021 Dec 13;18(12):e1003552. doi: 10.1371/journal.pmed.1003552 (PMC8716028; doi:10.1371/journal.pmed.1003552)
Supplement: S2 IRB — IRB, Institutional Review Board. (DOCX) [file pmed.1003552.s003.docx]

**JHSPH IRB Research Plan for Secondary Analysis of Existing Data**

**PI Name: Robert Blum**

**Study Title: Global Early Adolescent Study: Secondary Data Analysis**

**IRB No.: 8549**

**PI Version No. / Date: Version 4, April 4, 2019**

1. **I. Aims of the Study:** Describe the aims/objectives of the research and/or the project’s research questions or hypotheses. The Global Early Adolescent Study (GEAS) is the first international study exploring how gender norms evolve across adolescence and inform a spectrum of adolescent health outcomes, including sexual and mental health. The first phase, consisting of formative research and the face validity and pilot testing of instruments among early adolescents 10-14 years of age across 15 countries, has recently been completed. IRB oversight for all instrument development was provided for the first phase under IRB #5684.

The present application is for the second phase of the study, which is a longitudinal exploration of gender norms, their co-construction of gender cross-culturally, and their relation to health outcomes including sexual and reproductive health, healthy sexuality, mental health, and gender-based violence. The specific objectives are the following:

1. Understand how gender norms evolve over time and what factors at the individual, family, community and societal levels influence this process;
2. Evaluate how gender norms in early adolescence predict health outcomes in middle- to late-adolescence for boys and for girls, including sexual health, gender-based violence, and mental health;
3. Examine how empowerment may act as a mediator in this process as it relates to gender norms and informs behaviors and health seeking behaviors; and
4. Compare how this process differs across cultures and;
5. To assess the impact of gender transformative interventions on gender norms and, in turn, key adolescent health outcomes

This application will be used to analyze GEAS data from Shanghai (China), Cuenca (Ecuador), Belgium, Capetown (South Africa), Blantyre (Malawi) Santiago (Chile), New Orleans (USA) and three cities in Indonesia; Lampung, Denpasar and Semarang. **The PI and research team from JHSPH will not have any direct contact with human subjects in any of the research sites.**

**II. Background and Rationale:** Explain why this study is being done. Summarize briefly what is already known about the issue and reference previously published research, if relevant.

While its expressions may vary, gender inequality is pervasive and persistent across time and place. There is growing recognition that gender norms significantly shape sexual behaviors and contribute to negative sexual and reproductive health outcomes among adolescents including sexual coercion, sexually transmitted infections, and early pregnancies. Qualitative research from Phase 1 of the GEAS strongly suggests that patterns of gender norms structuring male and female behaviors reinforce gender unequal power dynamics during early adolescence. This process operates across numerous cultures and contributes to the maintenance patriarchal systems. The evidence suggests that such normative beliefs predispose to youth to subsequent gender-based violence, early school leaving, depression, and a host of negative sexual health outcomes, yet research lacks the measurement and longitudinal data to establish the temporality and strength of these associations. The GEAS is set to answer these questions using a longitudinal design and validated measures to explore these processes over time and across cultures.

Since initiating Phase 1 of the Global Early Adolescent Study nearly 4 years ago, awareness has grown globally that there is a need to focus on this age group both to understand the processes by which gender norms are reinforced and for the development of interventions that promote gender equitable relationships and norms. The study designed for Phase 2 is well suited to understand how these processes unfold and vary across cultures.

**III. Study Design:**

A. Provide an overview of your study design and methods. The study design must relate to your stated aims/objectives. Explain what existing data you plan to use and where it comes from.

The present study involves two types of study designs:

1. Observational Research: The GEAS is designed as a longitudinal survey study to compare changes in the perceptions of gender norms and subsequent effects on adolescent health outcomes in a critical period of human development. This study will analyze all data from Cuenca, Ecuador; Shanghai, China; several Flemish cities in Belgium; Capetown, South Africa; Blantyre, Malawi; Santiago, Chile, and data from the control groups of two sites: New Orleans and Indonesia. In each country, approximately 1400-1500 adolescents aged 10-14 are selected from the schools they are attending. In Belgium, this sample will be recruited from urban areas of Flemish Belgium.

*Analysis* will involve longitudinal cross-site analyses of observational data, involving only adolescents in the control group for the intervention sites. We anticipate using latent growth curve analysis to study the individual trajectories of gender norms perceptions, agency, and key health and well-being outcomes (sexual and reproductive health, violence, and mental health). Explanatory analysis will be used to assess the shapes of the curves of these dimensions over time, and we will perform cluster analysis to locate clusters of individuals that have different growth curves. Bivariate analysis will be used to identify the individual, and other contextual factors that affect key variable growth curves. The bivariate analysis will be expanded to conditional growth models to assess the independent effects of latent time-varying predictors (gender norms, empowerment, and knowledge), as well as time-invariant covariates (e.g. certain socio-demographics) on gender-based violence, romantic relationships and sexual behaviors. Multiple-group analysis will be conducted to assess whether the effects vary for males and females.

1. **Intervention Evaluations:** The second type of study design will be a quasi-experimental longitudinal design that will compare adolescents in two arms: intervention and control. Both interventions involve comprehensive sexuality education programs, though the curriculum differs between the two sites. The research will be conducted in three waves:
   1. Wave 1 aims to assess indicators at baseline
   2. Wave 2 aims to compare changes in perceptions of gender norms and health and behavioral outcomes between intervention and control groups approximately one year after implementation of each intervention
   3. Wave 3 aims to compare changes in perceptions of gender norms and health and behavioral outcomes between intervention and control groups approximately two years after implementation of each intervention.

Analysis for the intervention evaluation will include cross-sectional assessments indicators in matched samples across waves, and difference-in-difference analyses to assess intervention impact at individual-level between members of intervention and control groups

B. Provide a sample size and an explanation as to how you arrived at that number.

In each observational research site, approximately 1400-1500 adolescents aged between 10 and 14 years will be recruited for the study. Approximately 50% of the sample will be girls. With an attrition rate of 25%, we expect that about 1000 adolescents will be followed for five years. In Blantyre, approximately 2000 adolescents will be recruited as the study team expects a slightly higher rate of attrition. In-school sampling will be conducted at each site.

In Indonesia, the anticipated sample size of 4000 was calculated based on the prevalence of key indicators (gender norms and gender based violence), powered to detected differences in these key indicators between control and intervention over time. This calculation resulted in a sample of 3312 (1,656 in each arm), and with an assumption of a 20% attrition rate resulted in final planned sample of 4000 adolescents. Ultimately, data in Indonesia was collected from 4,542 adolescent/parent dyads.

In New Orleans, the sample size calculation is based off of the Institute of Women and Ethnic Studies’ Emotional Wellness Screener (EWS) data from previous sex education programming which has found depression symptomatology in 21% of youth served in New Orleans schools. Based on EWS data, which shows an average school-based cluster size of 45 and an intra-class correlation of 0.018, we estimate a design effect of 1.8. We allow for 40% attrition in each arm. It is estimated that the study will require a total enrollment of 1,684 students, 842 per study arm, in order to detect a ten percentage point difference in depression symptomology between study arms with a 90% power.

**Parental Surveys**: In Santiago and Indonesia, socio-economic status and household data is collected from a parent or guardian for each adolescent enrolled. A sample of parents in Shanghai (10%, 150) and New Orleans (20%, 337) will be in enrolled to obtain additional household SES information.

**IV. Participants:**

A. Describe the subjects who provided the original data and the population from which they were drawn.

The general sampling structure involves a two-stage process with the selection of schools and the selection of students within participating schools. School characteristics, including size, grade structure, enrollment selection, types (public/private; religious/ non-religious) and school distribution in communities vary greatly by site. Therefore, a common sampling protocol is not pertinent for this study. While we seek to include a diverse sample of the adolescent population living in each urban poor site, representativeness is not a prerequisite for this study because we do not aim to compare prevalence, but rather to compare social processes linking gender norms and health outcomes across diverse cultural settings.

In general for the observational research sites, all schools including standard 6^th^ grade will be listed in each site and schools from that list will be selected based on geography, school size and school type when pertinent. In Cuenca and Blantyre, a random sample of schools stratified by school characteristics will be conducted, while in Shanghai, three schools will be purposively selected based on the number of students from each age category 10 to 14 years old. In Capetown, three study communities will be purposively sampled based on ease of access and representativeness of Capetown population. Within the selected communities, approximately 10 schools will then be randomly selected to achieve desired sample size. Both primary and secondary schools will be selected to include adolescents in the 10-14 year old age group. In Belgium, an exhaustive sampling approach is employed. Adolescent participants will be sampled from secondary schools 1) with an OKI index between 1.2-4 (4 indicating most proportion of vulnerable students in that school) and 2) that enroll students from age 12 to age 18. Eligible schools are located in Flemish cities that are 1) within proximity for the study team in Ghent and 2) are Flemish-speaking. Therefore, excluded Belgian provinces will be Limburg and Brussels.

In Shanghai, Belgium, Capetown, and Santiago all students aged 10-14 years and/or in grades 6 – 8 will be eligible from the selected schools and invited to participate in the study. In Cuenca, a random selection of eligible students aged 10 to 14 years will be selected, after stratification by sex, either as a simple draw from a list of all students or after random selection of classes (most likely standard 6) in large schools. In Blantyre, adolescents will be sampled based on random number sampling to select 250 boys and 250 girls from each school register of Standard 6 students. To allow for some potential participants declining to participate in Blantyre, substitution with up to 100 alternate participants is planned, with names pre-selected as part of the random sample.

In each of the three cities in Indonesia, three intervention and three control schools are selected, based on where the intervention is being administered and matched control schools. Within each of the 18 intervention schools (9 intervention, 9 control), approximately 184 adolescents enrolled in 7^th^ grade were selected, for a total of approximately 4000 adolescents overall in Indonesia (1,656 in each study group, plus an expected 20% attrition). A parent or guardian of each enrolled adolescents was surveyed to collect socio-demographic and household composition data.

In New Orleans, schools are selected from the network of charter schools with which IWES has existing partnerships that have agreed to participate in the study. Block randomization, stratified by school performance, is used to assign schools to either study arm. All students ages 11-14 who are in a participating grade or class will be invited to participate. Parents of approximately 20% of the adolescent sample will be randomly sampled, stratified by school, and both sex and grade of the adolescent, with an anticipated parental sample of 337 parents. The anticipated sample sizes in New Orleans is 1,684 adolescents and 337 parents.

B. If you plan to analyze human specimens or genetic/genomic data, provide details about the source of those specimens and whether they were collected using an informed consent document. If yes, explain whether your proposed use is “consistent with” the scope of the original consent, if it potentially introduces new analyses beyond the scope of the original consent, and/or if it introduces new sensitive topics (HIV/STDs, mental health, addiction) or cultural/community issues that may be controversial.

Not applicable

1. Explain whether (and how) you plan to return results to the participants either individually or as a group.

Each site will produce a country-level report on their data, the template for which is standardized across sites. Sites participating in the GEAS have the ability to hold their own dissemination events, and to participate in GEAS project-wide dissemination at large.

**NOTE**:

## **V. Data Security and Confidentiality Protections:**

## **Personally Identifiable Information (PII):**

Please identify the Personally Identifiable Information (PII) that you may be obtaining and using in your study:

| Name, signature, initials, or other identifiable code |  |
| --- | --- |
| Geographic identifier: address, GPS location, etc. |  |
| Dates: birth, death, clinical service, discharge, etc. |  |
| Contact information: phone numbers, email address, etc. |  |
| ID: Social Security Number, driver’s license number, etc. |  |
| Health record identifiers: medical record, insurance plan number, etc. |  |
| Account numbers |  |
| Device identifiers: e.g., implants |  |
| Internet identifiers: IP address, social media accounts |  |
| Biometric identifiers, including finger and voice prints |  |
| Audio recordings |  |
| Video or full face photographic images |  |
| Genomic/genetic data |  |
| Any other unique identifying number, characteristic, or code (note: this does not mean the unique code assigned by the investigator to code the data) |  |
| Other: Click here to enter text. |  |

## **Data Collection:**

Collection of data for a research study can take on many forms. It can be as simple as gathering the data with pen and paper or developing an on-line adaptive survey that changes based on the participant’s answers. Regardless of the method, PII collection for the purposes of identifying the participants will most likely be collected. Once collected, the raw data should go through a de-identification process to further protect PII.

In what form will you access, receive, and/or store PII?

### **Hard Copy/Paper:** Yes No

If yes, please answer the following:

1. If applicable, how will the data be stored securely during transfer?

1. Will the data be secured in a locked cabinet or room? Yes  No
2. If study IDs/Codes are used, will they be stored separately from the study data? Yes  No
3. Will the hard copy/paper be destroyed after data abstraction and cleaning are complete?
4. Yes  No

### **Electronic:** Yes No

If yes, please answer the following:

1. Will the data be collected/stored on a portable device (laptop, mobile phone, tablet, PDA) protected by encryption? Yes  No
2. Will study participants use personally owned devices or study-provided devices?

Personally owned  Study provided

1. Is the application/website used for data collection being developed in-house (Hopkins) or by a 3^rd^ party vendor?

In-house  3^rd^ party

If 3^rd^ party, provide the name of vendor and URL: [SurveyCTO](https://www.surveycto.com/index.html)

Identify Mobile Ecosystem (check all that apply) Apple  Google  Website

1. Will the data be stored on a local secure server (@JHSPH/on-site) or in the Cloud/Web?

Secure Server  Cloud/ Web

1. Will it be encrypted? Yes  No
2. Will you be backing up your data? Yes  No

### **Audio Recording:** Yes No

If yes, please answer the following:

1. Will you store the audio recording securely in a locked cabinet/room until transcription is complete?

Yes  No

1. Will you use a transcription service that requires strong security protections to PII? If the PII comes from JHH/JHHS, you must use an approved vendor.

Yes  No

1. Will the audio recording be destroyed after transcription? Yes  No

If no, why not?

1. **Photograph/Video:** Yes  No

If yes, please answer the following:

1. Will the photographs/videos be stored securely in a locked cabinet or room? Yes  No
2. Will the photograph/video be destroyed? Yes  No

If yes, when?

### **PII De-Identification of Data Used for this Study:**

When will you destroy the PII and/or the code linking the PII with the study ID? Not applicable as we will only be getting de-identified data from the sites.

## **Data Storage and Analysis:**

One of the keys to protecting PII is the proper use of tools to share and conduct your analysis. JH and JHSPH offers several options for you to consider. Please select the system that you plan to use to protect your study data by clicking the box. Consult JHSPH IT for assistance if needed.

**JH Virtual Desktop**: ICTR provides a virtual Windows desktop (SAFE Desktop). It includes productivity software such as Microsoft Word and Excel, as well as statistical software, including SAS, Stata, R, R Studio, and Python. 100 GB of storage space is provided.

**JHSPH SharePoint and File Shares**: These systems provide a managed and secure platform for your research project. They also provide a built-in encrypted backup solution.

**JHSPH RedCAP or HPCC**: These are departmentally managed applications. RedCAP is an application designed for collaborative research projects. HPCC can provide the high capacity computing required for very large data sets.

**JHBox**: Johns Hopkins Box (JHBox) is a secure cloud-based file sharing and file storage service which enables people to collaborate and share information and may be accessed through any device: desktop, laptop, phone, or tablet.

**Independent Departmental Servers and Systems**: These servers are typically managed by departmental or research team IT staff.

**Other**: Please provide details regarding any other systems being utilized.

1. **Other Data Security Measures:**

Please review the following questions. This additional information will be utilized to assist in the development of a comprehensive Data Security plan. This would include the systems used to analyze the data, data security contacts and additional requirements.

1. During the analysis phase, do you plan to use computer systems that are not managed by JHSPH or JH? Yes  No

If yes, please explain:

1. Do you have a designated person on your research team other than the PI who is the technical contact for a Data Security plan? Yes  No

If yes, please provide a contact name: Mark Emerson and Linnea Zimmerman

1. Does your sponsor have other specific data security requirements for the study data? Yes  No

If possible, please explain:

Please add any other information that you believe is relevant to data security.

1. **Certificate of Confidentiality:** Does the study’s funder provide Certificate of Confidentiality protections? (See: https://humansubjects.nih.gov/coc/index)

Yes  No

1. Will you use clinical data of 500 records or more from Johns Hopkins Hospital and its affiliates?

Yes  No

If yes, please complete the JHM Data Security Checklist available on the JHSPH IRB website: [www.jhsph.edu/irb](http://www.jhsph.edu/irb) and upload a copy of the checklist to the “Miscellaneous” section.

**VI. Risks of the Study:**

Describe all relevant informational risks associated with a breach of confidentiality, including psychological, emotional, social, legal, or economic risks. With secondary data analysis, risks related to the original data collection do not need to be described.

If confidentiality is breached, the main risks would be that information about adolescents’ responses regarding sexual attitudes and behaviors, mental health, and adverse childhood experiences may be revealed.

**VII. Direct Personal and Social Benefits:**

Describe potential societal benefits likely to derive from the research, including value of knowledge learned.

We hypothesize that the findings generated from this research will enrich our understanding of how Inequitable gender norms may surface during early adolescence and their subsequent influence on health outcomes in later adolescence.

**VIII.** **Other IRBs/Ethics Review Boards:**

If other IRBs will review the research, provide the name and contact information for each IRB/ethics review board and its Federal Wide Assurance, if it has one (available on OHRP’s website at <http://www.hhs.gov/ohrp/assurances>).

| **Site** | **Collaborating Institution** | **Name of Local IRB** |
| --- | --- | --- |
| Cuenca | University of Cuenca | Comité de Bioetica, Universidad San Francisco de |
| Shanghai | Shanghai Institute of Planned Parenthood Research (SIPPR) | The Medical Ethical Committee of the Shanghai Institute of Planned Parenthood Research (SIPPR) |
| Belgium | University of Ghent | Universitair Ziekenhuis Ghent Commissie Voor Medische Ethiek |
| Capetown | University of the Western Cape | Biomedical Science Research Ethics Committee of the University of the Western Cape |
| Blantyre | University of Malawi College of Medicine | College of Medicine Research Ethics Committee |
| Santiago | University of Santiago, Chile | Comité de ética Institutcional, Universidad de Santiago de Chile |
| Indonesia | Universitas Gadja Mada | Medical and Health Research Ethics Committee (MHREC) Faculty of Medicine Gadja Mada University - Dr. Sardjito General Hospital Ethics Committee |
| New Orleans | Shanghai Institute of Planned Parenthood Research (SIPPR) | Institute of Women and Ethnic Studies Community Institutional Review Board |

**IX**. **Collaborations with non-JHSPH Institutions:**

For studies that involve collaboration with non-JHSPH institutions, complete the chart below by describing the collaboration and the roles and responsibilities of each partner, including the JHSPH investigator. This information helps us determine what IRB oversight is required for each party. Complete the chart for all multi-collaborator studies.

**Insert Name of Institutions in Partner column(s); add additional columns if necessary.**

|  | JHSPH | Partner 1  Shanghai Institute of Planned Parenthood | Partner 2  University of Cuenca | Partner 3  University of Ghent | Partner 4  University of the Western Cape | Partner 5 University of Malawi College of Medicine | Partner 6  University of Chile, Santiago | Partner 7  Rutgers, NE  Universitas Gadja Mada | Partner 8  Institute of Women and Ethnic Studies |
| --- | --- | --- | --- | --- | --- | --- | --- | --- | --- |
| Primary Grant Recipient |  | Dr. Lou Chaohua, Ph.D | Jose Ignacio Ortiz Segarra, Ph.D | Dr. Kristien Michielson, Ph.D | Diane Cooper, Ph.D | Dr. Williams Stones, MD, FRCOG | Dr. Matilde Maddaleno, MD | Miranda van Reeujwick  Rutgers NE and WPF is responsible for the implementation of the SETARA intervention. | Denese Shervington, MD, MPH |
| Collaborator | Robert Blum, Ph.D; Caroline Moreau, Ph.D; Kristin Mmari, Dr.PH | Dr. Zuo Xiayun, Ph.D | Jara Rodriguez and Gabriela Guerra | Sara de Meyer, MS | Suraya Mohamed, Ph.D  Lucia Knight, Ph.D  Hanani Tabana, Ph.D, MPH  Ntobeko Nywagi | Dr Getrude Chapotera MBBS, MPH, Ph.D  Mrs Effie Chipeta Ph.D  Dr Bernadette O’Hare, MB.BCh BAO, DCH, MRCP, DTM&H, MPH, FRCPCH, MD | D. Victoria Espinosa; Dra. Amaya Pavez; Dr. Jairo Vanegas; D. Lorena Ramirez; Dr. Jorge Puga; D. Francisca Davalos | Siswanto Agus Wilopo, Universitas Gadja Mada | Lisa Richardson, PhD |
