# Supplementary material for: Assessing the relationship between agency and peer violence among adolescents aged 10 to 14 years in Kinshasa, Democratic Republic of Congo and Blantyre, Malawi: A cross-sectional study
Source: PLoS Med. 2021 Dec 13;18(12):e1003552. doi: 10.1371/journal.pmed.1003552 (PMC8716028; doi:10.1371/journal.pmed.1003552)
Supplement: S1 Table — GEAS, Global Early Adolescent Study. (DOCX) [file pmed.1003552.s004.docx]

*S1_Table:* GEAS questions and measures

| **Questions** | **Response Options** |
| --- | --- |
| **Violence victimization and perpetration** | Yes, by a girl or girls |
| During the last 6 months, have you ever been slapped, hit or otherwise physically hurt by a boy or girl in a way that you did not want? | Yes, by a boy or boys |
| During the last 6 months, have you slapped, hit or otherwise physically hurt another boy or girl in a way that they did not want? | Yes, by both boys and girls |
|  | No |
|  | Don’t know |
| **Freedom of Movement Score: Can you tell me how often you are allowed to do the following alone (without an adult present)?** |  |
| Go to after-school activities (like sports clubs) | Often |
| Go to a party with boys and girls | Sometimes |
| Meet with friends after school | Rarely |
| Go to community center/movies/youth center | Never |
| Visit a friend of the opposite sex | Refuse |
| **Voice Score: How often are the following statements true for you?** | Don't Know |
| My parents or guardians ask for my opinion on things |  |
| My parents or guardians listen when I share my opinion |  |
| My friends ask my advice when they have a problem |  |
| If I see something wrong in school or the neighborhood I feel I can tell someone and they will listen |  |
| I can speak up in class when I have a comment or question |  |
| I can speak up when I see someone else being hurt |  |
| I can ask adults for help when I need it |  |
| **Decision Score : How often are you able to make the following decisions on your own, without an adult?** |  |
| What clothes to wear when you are not in school/working |  |
| What to do in your free time |  |
| What to eat when you are not at home |  |
| Who you can have as friends |  |
| **Covariates** |  |
| **Age** |  |
| How old are you? | 10 to 14 |
| **Gender** | Boy |
| Are you a..? | Girl |
| **School status** | Yes |
| Are you currently enrolled in school? | No |
|  | It is currently holiday break or vacation; otherwise I would be going to school |
|  | Refuse to answer |
| **Level of education** | Different response options based on site |
| What grade or class are you in? |  |
| **Wealth index**  *Which of the following do you have in your home that is in working order?*  Electricity  Indoor running water  Toilet (in home)  Shower/bath or both  Bicycle  Motor bike/motor scooter  Car or truck  Cell/mobile phone  Television/TV  Radio  Satellite dish/DSTV  DVD player  Refrigerator  Clothes washing machine  Dishwasher  Microwave | Yes  No  Don’t know |
| **Perceptions of gender stereotypical traits** |  |
| Boys should be raised tough so they can overcome any difficulty in life. Do you agree or disagree? | Agree a lot |
| Girls should avoid raising their voice to be lady like. Do you agree or disagree? | Agree a little |
| Boys should always defend themselves even if it means fighting. Do you agree or disagree? | Neither agree, nor disagree |
| Girls are expected to be humble. Do you agree or disagree? | Disagree a little |
| Girls need their parents protection more than boys. Do you agree or disagree? | Disagree a lot |
| Boys who behave like girls are considered weak. Do you agree or disagree? | Refuse to answer |
| It's important for boys to show they are tough even if they are nervous inside. Do you agree or disagree? |  |
| **Adverse childhood experiences** |  |
| IXB1. Now we would like to ask whether as a child you ever experienced any of these things. You may not want to tell us, and that is OK, but the reason we are asking is that it will help us better understand who you are and what you have experienced. | Often |
| Have you ever been scared or felt really bad because grown-ups called you names, said mean things to you, or said they didn’t want you? | Sometimes |
| Have you ever been scared that your parents or other adults were going to hurt you badly (so that you were injured or killed)? | Never |
| Have you ever felt like you are not loved or cared about? | Don’t know |
| Have you ever felt like you have no one that protects you? | Refuse to answer |
| Has there ever been a time of your life when you were totally on your own and had to take care of yourself for more than a short time? |  |
| Have your parents/guardians ever drunk too much alcohol or used drugs so they came home and were really abusive to you or your family? |  |
| Has there ever been a time when your family did not have enough food because they had no money? |  |
| Have you ever seen your mom being hit, beaten or threatened? |  |
| Have you ever seen your mother or father so sad that they couldn’t take care of you? |  |
| Have any of your parents ever been in prison/jail? |  |
| Has your family ever been forced to leave your home/house? |  |
| Has an adult ever touched you in your private parts except when being bathed? |  |
| Has an adult ever attempted or forced you to have sexual intercourse? |  |
| **Household composition** |  |
| Who lives in your home? | Mother |
|  | Father |
|  | Brothers |
|  | Sisters |
|  | Grandparents |
|  | Other relatives |
|  | Other |
|  | Don’t know |
|  | Refuse to answer |
| **Parent closeness** |  |
| Do you feel close to your main caregiver? (By close, we mean that you can talk to that person and tell them about personal and important things) | A lot |
|  | Somewhat |
|  | Not much |
|  | Not at all |
|  | Don’t know |
|  | Refuse to answer |
| **Parental monitoring and awareness** |  |
| IIC1. To what extent are these things true about your main caregiver? |  |
| a. Knows who my friends are by name | Very true |
| b. Knows my grades/how I am doing in school | Somewhat true |
| c. Usually knows where I am | Not very true |
|  | Not true at all |
|  | Don’t know |
|  | Refuse to answer |
| **Friend composition** |  |
| How many close friends (BOYS and/or GIRLS) do you have? (By close friends, I mean those with whom you can talk about feelings and share secrets.) |  |
| Male friends (record number of male friends) | 0 |
| Female friends (record number of female friends) | 1 |
|  | 2 |
|  | 3 |
|  | 4 |
|  | 5 |
|  | 6 or more |
|  | Don’t know |
|  | Refuse to answer |
| **Time spent with friends** |  |
| During a normal week, how often do you spend time hanging out (socializing) with your closest friends outside of school? | Very often (nearly everyday) |
|  | Often (3 – 4 times a week) |
|  | Not very often (1 or 2 times a week) |
|  | Never (no times per week) |
|  | Refuse to answer |
| **Social cohesion** |  |
| VA2. How likely is it that an adult in your neighborhood would do something like intervene if children or teenagers were… | Very likely  Somewhat likely  Not very likely  Not likely at all  Don’t know  Refuse to answer |
