# Supplementary material for: Assessing the relationship between agency and peer violence among adolescents aged 10 to 14 years in Kinshasa, Democratic Republic of Congo and Blantyre, Malawi: A cross-sectional study
Source: PLoS Med. 2021 Dec 13;18(12):e1003552. doi: 10.1371/journal.pmed.1003552 (PMC8716028; doi:10.1371/journal.pmed.1003552)
Supplement: S2 Table — (DOCX) [file pmed.1003552.s005.docx]

*S2_Table:* Differences in associations between agency and peer violence by site among adolescent boys and girls: results from pooled multivariate multinomial regression model across sites, including interaction terms by site

|  |  | *Boys (n=1872 )* |  | *Girls (n=1881)* | | |  |
| --- | --- | --- | --- | --- | --- | --- | --- |
|  | *victimization* | *Perpetration* | *victimization & perpetration* | *victimization* | *Perpetration* | *victimization & perpetration* |  |
| **Study site** |  |  |  |  |  |  |  |
| *Kinshasa, DRC* | ref | ref | ref | ref | ref | ref |  |
| *Blantyre, Malawi* | 2.1 (1.0 to 4.6) (p=0.06) | 1.3 (0.5 to 3.4) (p=0.64) | 2.2 (1.0 to 4.9) (p=0.05) | 1.2 (0.6 to 2.6) (p=0.59) | 0.8 (0.3 to 1.8) (p=0.54) | 1.7 (0.7 to 3.7) (p=0.22) |  |
| **Freedom of movement** |  |  |  |  |  |  |  |
| *Tertile 1* | ref | ref | ref | ref | ref | ref |  |
| *Tertile 2* | 2.0 (1.3 to 3.0) (p=0.002) | 1.5 (0.9 to 2.3) (p=0.095) | 1.6 (1.1 to 2.4) (p=0.008) | 0.8 (0.4 to 1.3) (p=0.34) | 0.8 (0.5 to 1.2) (p=0.24) | 1.1 (0.7 to 1.8) (p=0.74) |  |
| *Tertile 3* | 1.9 (1.1 to 3.3) (p=0.02) | 2.5 (1.5 to 4.2) (p<0.001) | 1.8 (1.2 to 2.8)(p=0.007) | 1.3 (0.6 to 2.7) (p=0.52) | 0.4 (0.2 to 1.0) (p=0.052) | 0.8 (0.4 to 1.8) (p=0.62) |  |
| *Freedom of movement*site (Kinshasa reference)* | | P=0.12 |  |  | P=0.004 |  |  |
| *#Malawi tertile 2* | 0.7 (0.3 to 1.4) (p=0.28) | 1.9 (0.7 to 5.0) (p=0.21) | 1.7 (0.8 to 3.5) (p=0.17) | 2.5 (1.2 to 5.3) (p=0.014) | 1.2 (0.5 to 3.2) (p=0.72) | 1.2 (0.5 to 2.5) (p=0.72) |  |
| *#Malawi tertile 3* | 0.6 (0.3 to 1.3) (p=0.17) | 1.1 (0.4 to 3.2) (p=0.81) | 2.1 (1.0 to 4.6) (p=0.054) | 1.1 (0.4 to 3.1) (p=0.83) | 6.6 (1.8 to 23.7) (p=0.004) | 3.0 (1.1 to 8.7) (p=0.04) |  |
| **Voice** |  |  |  |  |  |  |  |
| *Tertile 1* | ref | ref | ref | ref | ref | ref |  |
| *Tertile 2* | 1.1 (0.7 to 1.8) (p=0.56) | 0.8 (0.5 to 1.2 (p=0.27) | 1.3 (0.9 to 1.9) (p=0.12) | 0.7 (0.4 to 1.1) (p=0.15) | 1.0 (0.7 to 1.5) (p=0.95) | 0.6 (0.4 to 1.0) (p=0.050) |  |
| *Tertile 3* | 1.1 (0.6 to 1.9) (p=0.83) | 1.0 (0.6 to 1.7) (p=0.97) | 0.9 (0.5 to 1.4) (p=0.58) | 0.7 (0.4 to 1.4) (p=0.34) | 0.8 (0.5 to 1.5) (p=0.54) | 0.8 (0.4 to 1.4) (p=0.43) |  |
| *Voice *site (Kinshasa reference)* | | P=0.12 |  |  | P=0.84 |  |  |
| *#Malawi tertile 2* | 1.1 (0.5 to 2.6) (p=0.76) | 1.0 (0.4 to 2.8) (p=0.99) | 0.4 (0.2 to 0.8) (p=0.008) | 1.6 (0.7 to 3.4) (p=0.26) | 0.9 (0.3 to 2.6) (p=0.90) | 1.6 (0.7 to 3.7) (p=0.29) |  |
| *#Malawi tertile 3* | 1.1 (0.4 to 2.7) (p=0.85) | 0.6 (0.2 to 1.7) (p=0.33) | 0.6 (0.2 to 1.3) (p=0.19) | 1.0 (0.4 to 2.4) (p=0.96) | 1.0 (0.3 to 3.0) (p=0.99) | 1.4 (0.6 to 3.5) (p=0.46) |  |
| **Decision-making** |  |  |  |  |  |  |  |
| *Tertile 1* | ref | ref | ref | ref | ref | ref |  |
| *Tertile 2* | 0.7 (0.5 to 1.1) (p=0.17) | 1.2 (0.8 to 1.9) (p=0.37) | 1.9 (1.3 to 2.8) (p=0.001) | 1.1 (0.7 to 1.8) (p=0.69) | 0.8 (0.5 to 1.3) (p=0.46) | 1.3 (0.8 to 2.2) (p=0.24) |  |
| *Tertile 3* | 0.8 (0.5 to 1.3) (p=0.29) | 1.0 (0.6 to 1.7) (p=0.98) | 2.5 (1.6 to 3.8) (p<0.001) | 1.4 (0.9 to 2.3) (p=0.15) | 1.6 (1.04 to 2.4) (p=0.03) | 2.1 (1.3 to 3.3) (p=0.002) |  |
| *Decision making *site (Kinshasa reference)* | | P=046 |  |  | P=0.36 |  |  |
| *#Malawi tertile 2* | 1.9 (0.9 to 4.0) (p=0.08) | 0.8 (0.3 to 2.0) (p=0.62) | 0.8 (0.4 to 1.6) (p=0.49) | 1.5 (0.7 to 3.4) (p=0.26) | 0.9 (0.3 to 2.4) (p=0.86) | 0.7 (0.3 to 1.6) (p=0.44) |  |
| *#Malawi tertile 3* | 1.6 (0.7 to 3.7) (p=0.23) | 0.6 (0.2 to 1.8) (p=0.39) | 0.7 (0.3 to 1.6) (p=0.47) | 1.3 (0.6 to 3.0) (p=0.48) | 0.5 (0.2 to 1.4) (p=0.20) | 0.5 (0.2 to 1.1) (p=0.07) |  |

Models adjusted for age, education, Adverse Childhood Experiences, household composition, parental closeness, parental monitoring and awareness, friend composition and social cohesion.

#: Test for interaction
