# Supplementary material for: Assessing the relationship between agency and peer violence among adolescents aged 10 to 14 years in Kinshasa, Democratic Republic of Congo and Blantyre, Malawi: A cross-sectional study
Source: PLoS Med. 2021 Dec 13;18(12):e1003552. doi: 10.1371/journal.pmed.1003552 (PMC8716028; doi:10.1371/journal.pmed.1003552)
Supplement: S3 Table — (DOCX) [file pmed.1003552.s006.docx]

*S3_Table:* Overall Model with Interaction between Site and Agency Variables and excluding Out of School Participants

|  |  | *Boys (n=1478 )* |  | *Girls (n=1551)* | | |  |
| --- | --- | --- | --- | --- | --- | --- | --- |
|  | *victimization* | *Perpetration* | *victimization & perpetration* | *victimization* | *Perpetration* | *victimization & perpetration* |  |
| **Study site** |  |  |  |  |  |  |  |
| *Kinshasa, DRC* | ref | ref | ref | ref | ref | ref |  |
| *Blantyre, Malawi* | 1.5 (0.7 to 3.5) (p=0.31) | 1.2 (0.4 to 3.4) (p=0.73) | 3.2 (1.4 to 7.6) (p=0.008) | 1.1 (0.5 to 2.4) (p=0.84) | 0.8 (0.3 to 2.1) (p=0.68) | 1.6 (0.7 to 3.8) (p=0.29) |  |
| **Freedom of movement** |  |  |  |  |  |  |  |
| *Tertile 1* | ref | ref | ref | ref | ref | ref |  |
| *Tertile 2* | 1.8 (1.1 to 3.2) (p=0.03) | 1.4 (0.8 to 2.4) (p=0.26) | 1.9 (1.2 to 2.9) (p=0.005) | 0.7 (0.3 to 1.5) (p=0.38) | 0.8 (0.5 to 1.4) (p=0.42) | 0.8 (0.5 to 1.6) (p=0.60) |  |
| *Tertile 3* | 2.3 (1.2 to 4.4) (p=0.008) | 2.3 (1.3 to 4.3) (p=0.007) | 1.6 (0.9 to 2.6) (p=0.081) | 1.2 (0.5 to 3.2 (p=0.67) | 0.4 (0.2 to 1.2) (p=0.10) | 0.5 (0.1 to 1.4) (p=0.17) |  |
| *Freedom of movement*site (Kinshasa reference)* | | P=0.055 |  |  | P=0.009 |  |  |
| *#Malawi tertile 2* | 0.7 (0.3 to 1.6) (p=0.45) | 2.0 (0.7 to 5.7) (p=0.19) | 1.5 (0.7 to 3.2) (p=0.34) | 2.7 (1.1 to 6.5) (p=0.03) | 1.1 (0.4 to 3.2) (p=0.79) | 1.4 (0.6 to 3.4) (p=0.42) |  |
| *#Malawi tertile 3* | 0.5 (0.2 to 1.1) (p=0.09) | 1.2 (0.4 to 3.7) (p=0.69) | 2.4 (1.1 to 5.5) (p=0.03) | 1.2 (0.3 to 3.9) (p=0.81) | 6.4 (1.7 to 24.7) (p=0.007) | 5.1 (1.4 to 19.3) (p=0.02) |  |
| **Voice** |  |  |  |  |  |  |  |
| *Tertile 1* | ref | ref | ref | ref | ref | ref |  |
| *Tertile 2* | 0.9 (0.5 to 1.6) (p=0.73) | 0.6 (0.4 to 1.1) (p=0.10) | 1.5 (1.0 to 2.3) (p=0.078) | 0.6 (0.3 to 1.1) (p=0.09) | 1.1 (0.7 to 1.7) (p=0.79) | 0.6 (0.4 to 1.1) (p=0.08) |  |
| *Tertile 3* | 0.9 (0.5 to 1.8) (p=0.80) | 0.9 (0.5 to 1.8) (p=0.87) | 1.1 (0.6 to 1.9) (p=0.77) | 0.4 (0.2 to 0.9) (p=0.046) | 0.9 (0.5 to 1.7) (p=0.67) | 0.7 (0.4 to 1.6) (p=0.43) |  |
| *Voice *site (Kinshasa reference)* | | P=0.06 |  |  | P=0.74 |  |  |
| *#Malawi tertile 2* | 1.4 (0.6 to 3.3) (p=0.45) | 1.2 (0.4 to 3.5) (p=0.74) | 0.3 (0.1 to 0.7) (p=0.006) | 1.8 (0.8 to 4.2) (p=0.15) | 0.9 (0.3 to 2.5) (p=0.82) | 1.7 (0.7 to 4.2) (p=0.26) |  |
| *#Malawi tertile 3* | 1.2 (0.5 to 3.3) (p=0.67) | 0.6 (0.2 to 1.9) (p=0.36) | 0.5 (0.2 to 1.1) (p=0.09) | 1.8 (0.6 to 5.5) (p=0.29) | 1.0 (0.3 to 3.0) (p=0.94) | 1.6 (0.6 to 4.3) (p=0.37) |  |
| **Decision-making** |  |  |  |  |  |  |  |
| *Tertile 1* | ref | ref | ref | ref | ref | ref |  |
| *Tertile 2* | 0.5 (0.3 to 0.8) (p=0.01) | 1.1 (0.6 to 1.9) (p=0.80) | 1.9 (1.2 to 3.1) (p=0.006) | 1.3 (0.7 to 2.3) (p=0.43) | 0.8 (0.5 to 1.4) (p=0.53) | 1.7 (0.9 to 3.0) (p=0.10) |  |
| *Tertile 3* | 0.7 (0.4 to 1.3) (p=0.24) | 1.1 (0.6 to 2.1) (p=0.77) | 2.6 (1.6 to 4.3) (p<0.001) | 1.1 (0.6 to 2.1) (p=0.70) | 1.8 (1.1 to 2.9) (p=0.011) | 2.4 (1.3 to 4.3) (p=0.003) |  |
| *Decision making *site (Kinshasa reference)* | | P=0.12 |  |  | P=0.21 |  |  |
| *#Malawi tertile 2* | 3.1 (1.3 to 7.1) (p=0.008) | 0.9 (0.3 to 2.5) (p=0.89) | 0.8 (0.3 to 1.7) (p=0.51) | 1.3 (0.6 to 3.0) (p=0.53) | 0.9 (0.3 to 2.6) (p=0.89) | 0.6 (0.2 to 1.4) (p=0.23) |  |
| *#Malawi tertile 3* | 1.9 (0.8 to 4.6) (p=0.17) | 0.6 (0.2 to 1.8) (p=0.34) | 0.7 (0.3 to 1.7) (p=0.45) | 1.6 (0.7 to 4.0) (p=0.29) | 0.5 (0.2 to 1.3) (p=0.14) | 0.4 (0.2 to 0.9) (p=0.05) |  |

Models adjusted for age, education, Adverse Childhood Experiences, household composition, parental closeness, parental monitoring and awareness, friend composition and social cohesion.

#: Test for interaction
