# Supplementary material for: Assessing the relationship between agency and peer violence among adolescents aged 10 to 14 years in Kinshasa, Democratic Republic of Congo and Blantyre, Malawi: A cross-sectional study
Source: PLoS Med. 2021 Dec 13;18(12):e1003552. doi: 10.1371/journal.pmed.1003552 (PMC8716028; doi:10.1371/journal.pmed.1003552)
Supplement: S4 Table — (DOCX) [file pmed.1003552.s007.docx]

*S4_Table:* Factors related to peer violence among boys and girls in Kinshasa: results from multivariate multinomial regression model

|  |  | *Boys (n=1274 )* |  | *Girls (n=1266)* | | |  |
| --- | --- | --- | --- | --- | --- | --- | --- |
|  | *victimization* | *Perpetration* | *victimization & perpetration* | *victimization* | *Perpetration* | *victimization & perpetration* |  |
| **Freedom of movement** |  |  |  |  |  |  |  |
| *Tertile 1* | ref | ref | ref | ref | ref | ref |  |
| *Tertile 2* | 1.5 (0.9 - 2.5)  (p=0.17) | 0.9 (0.5 -1.6)  (p=0.74) | 1.1 (0.7 - 1.7)  (p=0.73) | 1.1 (0.7-1.8)  (p=0.63) | 1.3 (0.9 - 2.0)  (p=0.20) | 1.1 (0.7 - 1.8)  (p=0.58) |  |
| *Tertile 3* | 2.8 (1.7- 4.6)  (p<0.001) | 1.7 (1.1 - 2.6)  (p=0.03) | 1.9 (1.2 - 2.8)  (p=0.003) | 1.0 (0.6 - 1.8)  (p=0.88) | 0.9 (0.5 - 1.4)  (p=0.54) | 1.1 (0.7 - 1.8)  (p=0.74) |  |
| **Voice** |  |  |  |  |  |  |  |
| *Tertile 1* | ref | ref | ref | ref | ref | ref |  |
| *Tertile 2* | 1.2 (0.8- 2.0)  (p=0.42) | 1.1 (0.7 - 1.8)  (p=0.67) | 1.9 (1.2 - 2.9)  (p=0.003) | 0.8 (0.5 - 1.3)  (p=0.35) | 1.2 (0.8 - 1.9)  (p=0.38) | 0.8 (0.5 - 1.3)  (p=0.32) |  |
| *Tertile 3* | 1.2 (0.7 - 2.0)  (p=0.43) | 1.2 (0.7 - 1.9)  (p=0.52) | 1.8 (1.2 - 2.8)  (p=0.009) | 0.6 (0.3 - 1.0)  (p=0.052) | 0.8 (0.5 - 1.3)  (p=0.30) | 0.6 (0.4 - 1.0)  (p=0.07) |  |
| **Decision-making** |  |  |  |  |  |  |  |
| *Tertile 1* | ref | ref | ref | ref | ref | ref |  |
| *Tertile 2* | 0.6 (0.4 - 0.9)  (p=0.007) | 1.3 (0.8 - 2.0)  (p=0.30) | 2.1 (1.4 - 3.3)  (p=0.001) | 1.1 (0.7 - 1.7)  (p=0.81) | 0.7 (0.5 - 1.1)  (p=0.17) | 0.9 (0.5 - 1.4)  (p=0.61) |  |
| *Tertile 3* | 0.6 (0.4 - 1.)  (p=0.07) | 1.1 (0.6 - 1.9)  (p=0.70) | 3.0 (1.8 - 4.8)  (p<0.001) | 1.5 (0.9 - 2.6)  (p=0.14) | 1.3 (0.9 - 2.1)  (p=0.18) | 1.7 (1.02 - 2.7)  (p=0.04) |  |
| **Age (years)** |  |  |  |  |  |  |  |
| *10_12* | ref | ref | ref | ref | ref | ref |  |
| *13 - 14* | 0.6 (0.4 - 0.9)  (p=0.03) | 1.1 (0.7 - 1.6)  (p=0.71) | 0.6 (0.4 - 0.8)  (p=0.001) | 0.6 (0.4 - 0.97)  (p=0.04) | 1.0 (0.7 - 1.4)  (p=0.93) | 0.8 (0.5 - 1.2)  (p=0.23) |  |
| **Education** |  |  |  |  |  |  |  |
| *Out of school* | ref | ref | ref | ref | ref | ref |  |
| *Lower than age expected grade* | 0.5 (0.2 - 0.9)  (p=0.03) | 0.7 (0.3 - 1.3)  (p=0.21) | 1.0 (0.5 - 1.7)  (p=0.92) | 1.0 (0.5 - 2.0)  (p=0.94) | 1.1 (0.6 - 2.0)  (p=0.82) | 1.2 (0.7 - 2.4)  (p=0.50) |  |
| *Age expected grade or higher* | 0.7 (0.4 - 1.1)  (p=0.14) | 0.9 (0.5 - 1.4)  (p=0.55) | 1.4 (0.9 - 2.2)  (p=0.13) | 0.5 (0.3 - 0.9)  (p=0.02) | 0.8 (0.5 - 1.4)  (p=0.50) | 0.7 (0.4 - 1.3)  (p=0.28) |  |
| **Wealth asset** |  |  |  |  |  |  |  |
| *1st (poorest)* | ref | ref | ref | ref | ref | ref |  |
| *2nd* | 0.6 (0.3 - 0.99)  (p=0.048) | 1.0 (0.6 - 1.7)  (p=0.94) | 0.5 (0.3 - 0.8)  (p=0.005) | 0.8 (0.4 - 1.6)  (p=0.56) | 0.9 (0.5 - 1.5)  (p=0.63) | 0.9 (0.5 - 1.6)  (p=0.73) |  |
| *3rd* | 0.9 (0.5 - 1.7)  (p=0.78) | 0.9 (0.5 - 1.6)  (p=0.68) | 0.8 (0.4 - 1.3)  (p=0.28) | 1.0 (0.5 - 1.9)  (p=1.0) | 1.0 (0.5 - 1.7)  (p=0.88) | 1.2 (0.6 - 2.1)  (p=0.64) |  |
| *4th* | 0.8 (0.4 - 1.5)  (p=0.49) | 0.8 (0.4 - 1.5)  (p=0.47) | 0.4 (0.3 - 0.8)  (p=0.004) | 1.4 (0.7 - 2.6)  (p=0.33) | 1.1 (0.6 - 2.0)  (p=0.67) | 0.7 (0.3 - 1.3)  (p=0.25) |  |
| *5th (richest)* | 0.6 (0.3 - 1.2)  (p=0.13) | 0.5 (0.3 - 1.1)  (p=0.07) | 0.6 (0.4 - 1.0)  (p=0.06) | 1.5 (0.7 - 2.9)  (p=0.29) | 1.1 (0.6 - 2.0)  (p=0.84) | 1.0 (0.5 - 2.0)  (p=1.0) |  |
| **Adverse Childhood Experiences** |  |  |  |  |  |  |  |
| *No ACEs* | ref | ref | ref | ref | ref | ref |  |
| *History of 1 ACEs* | 1.3 (0.7 - 2.5)  (p=0.36) | 2.5 (1.3 - 4.7)  (p=0.006) | 1.8 (1.0 - 3.1)  (p=0.06) | 2.4 (1.1 - 5.0)  (p=0.02) | 1.9 (1.1 - 3.3)  (p=0.03) | 2.1 (1.1 - 4.2)  (p=0.03) |  |
| *History of 2 ACEs* | 1.8 (0.99 - 3.4)  (p=0.054) | 2.5 (1.3 - 4.7)  (p=0.007) | 1.9 (1.1 - 3.5)  (p=0.03) | 3.4 (1.6 - 7.1)  (p=0.001) | 2.7 (1.5 - 4.7)  (p=0.001) | 2.0 (1.0 - 4.1)  (p=0.06) |  |
| *History of 3 ACEs* | 2.4 (1.2 - 4.7)  (p=0.009) | 3.0 (1.5 - 6.1)  (p=0.002) | 3.7 (2.0 - 6.8)  (p<0.001) | 4.4 (2.0 - 9.6)  (p<0.001) | 2.9 (1.5 - 5.4)  (p=0.001) | 3.3 (1.6 - 6.9)  (p=0.002) |  |
| *History of 4 or more ACEs* | 1.9 (1.01 - 3.6)  (p=0.048) | 1.6 (0.8 - 3.3)  (p=0.18) | 4.4 (2.5 - 7.7)  (p<0.001) | 8.6 (4.2 - 17.4)  (p<0.001) | 3.6 (2.0 - 6.4)  (p<0.001) | 7.2 (3.8 - 13.6)  (p<0.001) |  |
| **Household Composition** |  |  |  |  |  |  |  |
| *Dual parent* | ref | ref | ref | ref | ref | ref |  |
| *Single parent* | 0.8 (0.5 - 1.2)  (p=0.26) | 1.1 (0.7 - 1.7)  (p=0.64) | 1.1 (0.8 - 1.6)  (p=0.57) | 0.9 (0.6 - 1.4)  (p=0.65) | 0.9 (0.6 - 1.3)  (p=0.53) | 1.1 (0.7 - 1.7)  (p=0.71) |  |
| *Grandparent/other* | 0.9 (0.5 - 1.6)  (p=0.73) | 1.5 (0.9 - 2.5)  (p=0.16) | 1.4 (0.9 - 2.2)  (p=0.17) | 0.9 (0.5 - 1.7)  (p=0.79) | 0.9 (0.5 - 1.5)  (p=0.59) | 0.8 (0.4 - 1.5)  (p=0.46) |  |
| **Parent closeness** |  |  |  |  |  |  |  |
| *No* | ref | ref | ref | ref | ref | ref |  |
| *Yes* | 1.6 (1.04 - 2.4)  (p=0.03) | 1.1 (0.8 - 1.7)  (p=0.56) | 1.2 (0.9 - 1.7)  (p=0.28) | 1.3 (0.8 - 2.0)  (p=0.30) | 0.5 (0.4 - 0.8)  (p=0.001) | 0.5 (0.3 - 0.8)  (p=0.002) |  |
| **Parental monitoring and awareness** |  |  |  |  |  |  |  |
| *No* | ref | ref | ref | ref | ref | ref |  |
| *Yes* | 0.8 (0.5 - 1.2)  (p=0.24) | 0.7 (0.5 - 0.99)  (p=0.046) | 0.6 (0.4 - 0.8)  (p=0.001) | 1.1 (0.7 - 1.7)  (p=0.69) | 0.8 (0.5 - 1.2)  (p=0.23) | 0.9 (0.6 - 1.4)  (p=0.75) |  |
| **Friend composition** |  |  |  |  |  |  |  |
| *Same gender friends* | ref | ref | ref | ref | ref | ref |  |
| *Any opposite gender friends* | 0.7 (0.5 - 1.0)  (p=0.09) | 0.8 (0.5 - 1.1)  (p=0.15) | 0.8 (0.6 - 1.1)  (p=0.24) | 1.0 (0.7 - 1.6)  (p=0.94) | 1.1 (0.8 - 1.6)  (p=0.62) | 0.9 (0.6 - 1.4)  (p=0.73) |  |
| **Time spent with friends** |  |  |  |  |  |  |  |
| *Not everyday* | ref | ref | ref | ref | ref | ref |  |
| *Everyday* | 1.2 (0.8 - 1.7)  (p=0.48) | 1.1 (0.7 - 1.6)  (p=0.68) | 1.1 (0.8 - 1.5)  (p=0.76) | 1.2 (0.8 - 1.9)  (p=0.29) | 0.8 (0.5 - 1.2)  (p=0.31) | 1.1 (0.7 - 1.6)  (p=0.68) |  |
| **Social cohesion** |  |  |  |  |  |  |  |
| *Low* | ref | ref | ref | ref | ref | ref |  |
| *High* | 0.8 (0.6 - 1.3)  (p=0.51) | 0.6 (0.4 - 0.9)  (p=0.02) | 0.8 (0.6 - 1.2)  (p=0.25) | 1.0 (0.7 - 1.6)  (p=0.87) | 2.0 (1.3 - 3.0)  (p=0.001) | 2.1 (1.3 - 3.3)  (p=0.003) |  |
| **Gender Stereotypical Traits** |  |  |  |  |  |  |  |
| *Tertile 1* | Data not available^a^ | | | | | |  |
| *Tertile 2* | ref | ref | ref | ref | ref | ref |  |
| *Tertile 3* | 0.9 (0.6 - 1.3)  (p=0.46) | 1.1 (0.8 - 1.6)  (p=0.59) | 1.1 (0.8 - 1.5) (p=0.56) | 1.2 (0.8 - 1.9)  (p=0.31) | 1.4 (1.0 - 2.0)  (p=0.09) | 1.7 (1.1 - 2.6)  (p=0.01) |  |

ACEs: Adverse Childhood Experiences

a: Data not available because of lack of variation in the scores.
