# Supplementary material for: Assessing the relationship between agency and peer violence among adolescents aged 10 to 14 years in Kinshasa, Democratic Republic of Congo and Blantyre, Malawi: A cross-sectional study
Source: PLoS Med. 2021 Dec 13;18(12):e1003552. doi: 10.1371/journal.pmed.1003552 (PMC8716028; doi:10.1371/journal.pmed.1003552)
Supplement: S5 Table — (DOCX) [file pmed.1003552.s008.docx]

*S5_Table:* Factors related to peer violence among boys and girls in Malawi: results from multivariate multinomial regression model

| *Factors related to peer violence* |  | *Boys (n=598 )* |  | *Girls (n=615)* | | |  |  |
| --- | --- | --- | --- | --- | --- | --- | --- | --- |
|  | *victimization* | *Perpetration* | *victimization & perpetration* | *victimization* | *Perpetration* | *victimization & perpetration* |  |  |
| **Freedom of movement** |  |  |  |  |  |  |  |  |
| *Tertile 1* | ref | ref | ref | ref | ref | ref |  |  |
| *Tertile 2* | 2.1 (1.1 - 3.9)  (p=0.02) | 7.2 (2.0 - 26.4)  (p=0.003) | 2.4 (1.1 - 5.5)  (p=0.03) | 1.1 (0.6 - 1.8)  (p=0.89) | 0.9 (0.4 - 2.0)  (p=0.77) | 1.1 (0.6 - 2.0)  (p=0.84) |  |  |
| *Tertile 3* | 1.3 (0.7 - 2.5)  (p=0.45) | 5.9 (1.6 - 21.9)  (p=0.009) | 3.8 (1.7 - 8.3)  (p=0.001) | 1.9 (0.9 - 3.8)  (p=0.08) | 2.5 (0.9 - 6.8)  (p=0.08) | 2.4 (1.1 - 5.1)  (p=0.03) |  |  |
| **Voice** |  |  |  |  |  |  |  |  |
| *Tertile 1* | ref | ref | ref | ref | ref | ref |  |  |
| *Tertile 2* | 1.3 (0.7 - 2.3)  (p=0.45) | 0.7 (0.3 - 1.7)  (p=0.44) | 0.7 (0.4 - 1.4)  (p=0.37) | 1.9 (1.1 - 3.1)  (p=0.02) | 1.8 (0.8 - 4.5)  (p=0.18) | 2.0 (1.01 - 3.9)  (p=0.048) |  |  |
| *Tertile 3* | 1.0 (0.5 - 1.9)  (p=1.0) | 0.4 (0.1 - 1.2)  (p=0.09) | 0.7 (0.4 - 1.4)  (p=0.29) | 0.8 (0.4 - 1.5)  (p=0.47) | 1.0 (0.4 - 2.7)  (p=1.0) | 1.3 (0.7 - 2.7)  (p=0.40) |  |  |
| **Decision-making** |  |  |  |  |  |  |  |  |
| *Tertile 1* | ref | ref | ref | ref | ref | ref |  |  |
| *Tertile 2* | 1.5 (0.8 - 2.7)  (p=0.20) | 0.9 (0.4 - 2.1)  (p=0.83) | 1.2 (0.7 - 2.4)  (p=0.51) | 1.8 (1.0 - 3.2)  (p=0.06) | 0.8 (0.3 - 1.8)  (p=0.52) | 0.8 (0.4 - 1.6)  (p=0.59) |  |  |
| *Tertile 3* | 1.4 (0.7 - 2.6)  (p=0.36) | 0.6 (0.2 - 1.7)  (p=0.36) | 1.7 (0.8 - 3.3)  (p=0.14) | 1.7 (0.9 - 3.3)  (p=0.10) | 0.8 (0.3 - 2.1)  (p=0.68) | 1.0 (0.5 - 2.0)  (p=0.94) |  |  |
| **Age (years)** |  |  |  |  |  |  |  |  |
| *10_12* | ref | ref | ref | ref | ref | ref |  |  |
| *13 - 14* | 0.4 (0.2 - 0.7) (p=0.001) | 0.5 (0.2 - 1.1) (p=0.07) | 0.8 (0.4 - 1.4) (p=0.44) | 0.8 (0.4 - 1.5)  (p=0.45) | 0.8 (0.3 - 1.8)  (p=0.54) | 0.6 (0.3 - 1.2)  (p=0.14) |  |  |
| **Education** |  |  |  |  |  |  |  |  |
| *Lower than age expected grade* | ref | ref | ref | ref | ref | ref |  |  |
| *Age expected grade or higher* | 0.5 (0.3 - 0.97) (p=0.04) | 0.6 (0.3 - 1.5)  (p=0.27) | 0.8 (0.4 - 1.5)  (p=0.52) | 1.4 (0.8 - 2.5)  (p=0.29) | 0.8 (0.3 - 2.1)  (p=0.69) | 0.8 (0.4 - 1.7)  (p=0.63) |  |  |
| **Adverse Childhood Experiences** |  |  |  |  |  |  |  |  |
| *No ACEs* | ref | ref | ref | ref | ref | ref |  |  |
| *History of 1 ACEs* | 1.4 (0.7 - 2.8)  (p=0.32) | 0.8 (0.2 - 3.0)  (p=0.79) | 2.5 (1.01 - 6.3)  (p=0.046) | 1.3 (0.7 - 2.6)  (p=0.46) | 0.9 (0.3 - 2.5)  (p=0.79) | 1.5 (0.6 - 3.9)  (p=0.44) |  |  |
| *History of 2 ACEs* | 1.9 (0.9 - 3.9)  (p=0.08) | 4.1 (1.4 - 12.1) (p=0.01) | 2.6 (1.09 - 7.1)  (p=0.052) | 1.8 (0.9 - 3.6)  (p=0.13) | 1.2 (0.4 - 3.7)  (p=0.78) | 4.7 (2.0 - 11.3)  (p<0.001) |  |  |
| *History of 3 ACEs* | 2.5 (1.1 - 5.7) (p=0.03) | 3.5 (1.03 - 12.2)  (p=0.04) | 9.2 (3.5 - 24.0)  (p<0.001) | 2.5 (1.1 - 5.4)  (p=0.03) | 3.6 (1.3 - 10.1)  (p=0.01) | 4.8 (1.8 - 12.9)  (p=0.002) |  |  |
| *History of 4 or more ACEs* | 3.5 (1.8 - 7.0) (p<0.001) | 3.8 (1.3 - 11.1)  (p=0.01) | 11.0 (4.7 - 26.1)  (p<0.001) | 5.3 (2.6 - 11.1)  (p<0.001) | 2.9 (1.0 - 8.8)  (p=0.053) | 16.8 (7.0 - 40.2)  (p<0.001) |  |  |
| **Household Composition** |  |  |  |  |  |  |  |  |
| *Dual parent* | ref | ref | ref | ref | ref | ref |  |  |
| *Single parent* | 1.2 (0.7 - 1.9)  (p=0.47) | 1.4 (0.7 - 2.8)  (p=0.38) | 1.3 (0.8 - 2.1)  (p=0.34) | 1.0 (0.6 - 1.7)  (p=0.95) | 0.7 (0.3 - 1.4)  (p=0.33) | 1.0 (0.5 - 1.7)  (p=0.94) |  |  |
| *Grandparent/other* | 1.0 (0.4 - 2.3)  (p=1.0) | 1.1 (0.3 - 3.8)  (p=0.93) | 0.4 (0.1 - 1.1)  (p=0.08) | 1.2 (0.5 - 3.0)  (p=0.72) | 0.3 (0.04 - 2.4)  (p=0.26) | 1.3 (0.5 - 3.5)  (p=0.64) |  |  |
| **Parent closeness** |  |  |  |  |  |  |  |  |
| *No* | ref | ref | ref | ref | ref | ref |  |  |
| *Yes* | 1.0 (0.6 - 1.6) (p=0.9) | 1.6 (0.7 - 3.6)  (p=0.27) | 1.0 (0.6 - 1.8)  (p=0.87) | 0.5 (0.3 - 0.9)  (p=0.03) | 0.9 (0.4 - 2.2)  (p=0.85) | 0.7 (0.4 - 1.4)  (p=0.33) |  |  |
| **Parental monitoring and awareness** |  |  |  |  |  |  |  |  |
| *No* | Data not available^a^ | | ref | ref | ref | ref |  | |
| *Yes* |  |  | 0.9 (0.1 - 5.3)  (p=0.90) | 0.8 (0.1 - 4.7)  (p=0.76) | 0.4 (0.03 - 4.7)  (p=0.48) | 0.5 (0.1 - 3.6)  (p=0.51) |  |  |
| **Friend composition** |  |  |  |  |  |  |  |  |
| *Same gender friends* | ref | ref | ref | ref | ref | ref |  |  |
| *Any opposite gender friends* | 1.2 (0.7 - 2.1) (p=0.44) | 3.9 (1.3 - 12.1) (p=0.02) | 1.6 (0.8 - 3.0)  (p=0.16) | 1.6 (0.9 - 2.8)  (p=0.11) | 1.6 (0.7 - 3.8)  (p=0.28) | 2.1 (1.04 - 4.3)  (p=0.04) |  |  |
| **Social cohesion** |  |  |  |  |  |  |  |  |
| *Low* | ref | ref | ref | ref | ref | ref |  |  |
| *High* | 1.7 (1.03 - 2.8) (p=0.04) | 1.5 (0.7 - 3.3)  (p=0.25) | 3.2 (1.8 - 5.7)  (p<0.001) | 1.6 (0.9 - 2.8)  (p=0.09) | 1.5 (0.6 - 3.6)  (p=0.31) | 1.4 (0.8 - 2.5)  (p=0.30) |  |  |
| **Gender Stereotypical Traits** |  |  |  |  |  |  |  |  |
| *Tertile 1* | ref | ref | ref | ref | ref | ref |  |  |
| *Tertile 2* | 0.9 (0.5 - 1.5)  (p=0.6) | 1.0 (0.5 - 2.3)  (p=0.93) | 0.9 (0.5 - 1.6)  (p=0.61) | 0.6 (0.4 - 1.1)  (p=0.11) | 1.0 (0.4 - 2.3)  (p=0.98) | 0.7 (0.4 - 1.4)  (p=0.36) |  |  |
| *Tertile 3* | 0.8 (0.4 - 1.5)  (p=0.4) | 0.8 (0.3 - 2.1)  (p=0.72) | 1.2 (0.6 - 2.2)  (p=0.66) | 1.4 (0.8 - 2.5)  (p=0.29) | 1.1 (0.4 - 2.8)  (p=0.88) | 0.9 (0.4 - 1.8)  (p=0.71) |  |  |

ACEs: Adverse Childhood Experiences

a: Data not available because the sample size was too small to detect change.
