# Supplementary material for: Assessing the relationship between agency and peer violence among adolescents aged 10 to 14 years in Kinshasa, Democratic Republic of Congo and Blantyre, Malawi: A cross-sectional study
Source: PLoS Med. 2021 Dec 13;18(12):e1003552. doi: 10.1371/journal.pmed.1003552 (PMC8716028; doi:10.1371/journal.pmed.1003552)
Supplement: S1 Codebook — (PDF) [file pmed.1003552.s010.pdf]

```
      name:  <unnamed>
       log:  /Users/akramaiya/Dropbox/Personal Work/GEAS/GEAS Empowerment Working Group/Empowerment and Violence Perpetration & Victimization/PLoS Medicine
> /Revise and Resubmit - 3/codebook.smcl
  log type:  smcl
 opened on:  19 Nov 2021, 16:41:57

1 . codebook

ia1age                                                                                                                      IA1. How old are you?

                  type:  numeric (byte)

                 range:  [10,14]                      units:  1
         unique values:  5                        missing .:  0/4,536

            tabulation:  Freq.  Value
                           770  10
                           988  11
                         1,116  12
                           981  13
                           681  14

ia2gender                                                                                                                    IA2. Are you a ….?

                  type:  numeric (byte)
                 label:  ia2gender

                 range:  [0,1]                        units:  1
         unique values:  2                        missing .:  0/4,536

            tabulation:  Freq.   Numeric  Label
                         2,218         0  Boy
                         2,318         1  Girl

va2a                                                                                                                      Damaging property

                  type:  numeric (int)
                 label:  va2a

                 range:  [1,4]                        units:  1
         unique values:  4                        missing .:  357/4,536

            tabulation:  Freq.   Numeric  Label
                           254         1  Not likely at all
                           604         2  Not very likely
                           795         3  Somewhat likely
                         2,526         4  Very likely
                           357         .

va2b                                                                                                    Spraying paint on walls (Graffiti)

                  type:  numeric (int)
                 label:  va2b

                 range:  [1,4]                        units:  1
         unique values:  4                        missing .:  275/4,536

            tabulation:  Freq.   Numeric  Label
                           259         1  Not likely at all
                           448         2  Not very likely
                           814         3  Somewhat likely
                         2,740         4  Very likely
                           275         .

va2c                                                                                                   Bullying or threatening another person

                  type:  numeric (int)
                 label:  va2c

                 range:  [1,4]                        units:  1
         unique values:  4                        missing .:  224/4,536

            tabulation:  Freq.   Numeric  Label
                           274         1  Not likely at all
                           488         2  Not very likely
                           883         3  Somewhat likely
                         2,667         4  Very likely
                           224         .

va2d                                                                                                          Fighting with another person

                  type:  numeric (int)
                 label:  va2d

                 range:  [1,4]                        units:  1
         unique values:  4                        missing .:  151/4,536

            tabulation:  Freq.   Numeric  Label
                           245         1  Not likely at all
                           525         2  Not very likely
                           799         3  Somewhat likely
                         2,816         4  Very likely
                           151         .

ixb1a                                         Have you ever been scared or felt really bad because grown-ups called you names,

                  type:  numeric (int)
                 label:  ixb1a

                 range:  [1,999]                      units:  1
         unique values:  4                        missing .:  29/4,536

            tabulation:  Freq.   Numeric  Label
                         2,672         1  Never
                         1,222         2  Sometimes
                           501         3  Often
                           112       999  Don't know
                            29         .

ixb1b                                         Have you ever been scared that your parents or other adults were going to hurt y

                  type:  numeric (int)
                 label:  ixb1b

                 range:  [1,999]                      units:  1
         unique values:  4                        missing .:  36/4,536

            tabulation:  Freq.   Numeric  Label
                         3,369         1  Never
                           777         2  Sometimes
                           212         3  Often
                           142       999  Don't know
                            36         .

ixb1c                                                                    Have you ever felt like you are not loved or cared about?

                  type:  numeric (int)
                 label:  ixb1c

                 range:  [1,999]                      units:  1
         unique values:  4                        missing .:  32/4,536

            tabulation:  Freq.   Numeric  Label
                         3,636         1  Never
                           511         2  Sometimes
```

```
                      220         3  Often
                      137       999  Don't know
                       32         .
```

---

ixb1d — **Have you ever felt like you have no one that protects you?**

```
              type:  numeric (int)
             label:  ixb1d

             range:  [1,999]                     units:  1
     unique values:  4                       missing .:  39/4,536

        tabulation:  Freq.   Numeric  Label
                     3,648         1  Never
                       436         2  Sometimes
                       268         3  Often
                       145       999  Don't know
                        39         .
```

---

ixb1e — **Has there ever been a time of your life when you were totally on your own and ha**

```
              type:  numeric (int)
             label:  ixb1e

             range:  [1,999]                     units:  1
     unique values:  4                       missing .:  31/4,536

        tabulation:  Freq.   Numeric  Label
                     3,764         1  Never
                       469         2  Sometimes
                       174         3  Often
                        98       999  Don't know
                        31         .
```

---

ixb1f — **Have your parents/guardians ever drunk too much alcohol or used drugs so they ca**

```
              type:  numeric (int)
             label:  ixb1f

             range:  [1,999]                     units:  1
     unique values:  4                       missing .:  32/4,536

        tabulation:  Freq.   Numeric  Label
                     4,048         1  Never
                       238         2  Sometimes
                        99         3  Often
                       119       999  Don't know
                        32         .
```

---

ixb1g — **Has there ever been a time when your family did not have enough food because the**

```
              type:  numeric (int)
             label:  ixb1g

             range:  [1,999]                     units:  1
     unique values:  4                       missing .:  23/4,536

        tabulation:  Freq.   Numeric  Label
                     2,579         1  Never
                     1,433         2  Sometimes
                       412         3  Often
                        89       999  Don't know
                        23         .
```

---

ixb1h — **Have you ever seen your mom being hit, beaten or threatened?**

```
              type:  numeric (int)
             label:  ixb1h

             range:  [1,999]                     units:  1
     unique values:  4                       missing .:  31/4,536

        tabulation:  Freq.   Numeric  Label
                     3,501         1  Never
                       717         2  Sometimes
                       171         3  Often
                       116       999  Don't know
                        31         .
```

---

ixb1i — **Have you ever seen your mother or father so sad that they couldn't take care of**

```
              type:  numeric (int)
             label:  ixb1i

             range:  [1,999]                     units:  1
     unique values:  4                       missing .:  34/4,536

        tabulation:  Freq.   Numeric  Label
                     3,663         1  Never
                       566         2  Sometimes
                       159         3  Often
                       114       999  Don't know
                        34         .
```

---

ixb1j — **Have any of your parents ever been in prison/jail?**

```
              type:  numeric (int)
             label:  ixb1j

             range:  [1,999]                     units:  1
     unique values:  4                       missing .:  26/4,536

        tabulation:  Freq.   Numeric  Label
                     3,884         1  Never
                       443         2  Sometimes
                        55         3  Often
                       128       999  Don't know
                        26         .
```

---

ixb1k — **Has your family ever been forced to leave your home/house?**

```
              type:  numeric (int)
             label:  ixb1k

             range:  [1,999]                     units:  1
     unique values:  4                       missing .:  34/4,536

        tabulation:  Freq.   Numeric  Label
                     3,895         1  Never
                       378         2  Sometimes
                       104         3  Often
                       125       999  Don't know
                        34         .
```

---

ixb1l — **Has an adult ever touched you in your private parts except when being bathed?**

```
              type:  numeric (int)
             label:  ixb1l

             range:  [1,999]                     units:  1
     unique values:  4                       missing .:  40/4,536

        tabulation:  Freq.   Numeric  Label
                     4,198         1  Never
                       182         2  Sometimes
                        52         3  Often
```

Freq.	Numeric	Label
64	999	Don't know
40	.	

---

ixblm — **Has an adult ever attempted or forced you to have sexual intercourse?**

type: numeric (**int**)
label: **ixblm**

range: [**1**,**999**] units: **1**
unique values: **4** missing .: **31**/**4,536**

tabulation:

Freq.	Numeric	Label
4,267	1	Never
146	2	Sometimes
35	3	Often
57	999	Don't know
31	.	

---

ixc10 — **IXC10. During the last 6 months, have you ever been slapped, hit or otherwise ph**

type: numeric (**int**)
label: **ixc10**

range: [**1**,**999**] units: **1**
unique values: **5** missing .: **17**/**4,536**

tabulation:

Freq.	Numeric	Label
3,197	1	No
213	2	Yes, by both boys and girls
563	3	Yes, by a boy or boys
500	4	Yes, by a girl or girls
46	999	Don't know
17	.	

---

ixc12 — **IXC12.During the last 6 months, have you slapped, hit or otherwise physcially hu**

type: numeric (**int**)
label: **ixc12**

range: [**1**,**999**] units: **1**
unique values: **5** missing .: **18**/**4,536**

tabulation:

Freq.	Numeric	Label
3,334	1	No
229	2	Yes, both boys and girls
456	3	Yes, a boy or boys
436	4	Yes, a girl or girls
63	999	Don't know
18	.	

---

meanscore_fom — **Freedom of Movement**

type: numeric (**float**)

range: [**1**,**4**] units: **1.000e-07**
unique values: **31** missing .: **28**/**4,536**

mean: **1.71627**
std. dev: **.760325**

percentiles:

10%	25%	50%	75%	90%
1	1	1.6	2.2	2.8

---

meanscore_voice — **Voice**

type: numeric (**float**)

range: [**1**,**4**] units: **1.000e-07**
unique values: **55** missing .: **20**/**4,536**

mean: **2.61619**
std. dev: **.746032**

percentiles:

10%	25%	50%	75%	90%
1.6	2	2.66667	3.14286	3.57143

---

meanscore_decision — **Decision-making**

type: numeric (**float**)

range: [**1**,**4**] units: **1.000e-07**
unique values: **19** missing .: **33**/**4,536**

mean: **2.76721**
std. dev: **.869571**

percentiles:

10%	25%	50%	75%	90%
1.5	2	3	3.5	4

---

education — **Age-for-grade Educational Attainment**

type: numeric (**float**)
label: **education**

range: [**0**,**2**] units: **1**
unique values: **3** missing .: **1**/**4,536**

tabulation:

Freq.	Numeric	Label
826	0	Out of school
1,517	1	Lower than age expected grade
2,192	2	Age expected grade or higher
1	.	

---

quintile — **Family Wealth Index**

type: numeric (**byte**)
label: **quintile**

range: [**1**,**5**] units: **1**
unique values: **5** missing .: **1,717**/**4,536**

tabulation:

Freq.	Numeric	Label
587	1	Bottom 20 percentile
594	2	20-40 percentile
530	3	40-60 percentile
563	4	60-80 percentile
545	5	Top 20 percentile
1,717	.	

---

parent_close — **Feels close to caregiver (a lot)**

type: numeric (**float**)
label: **parent_close**

range: [**0**,**1**] units: **1**
unique values: **2** missing .: **111**/**4,536**

tabulation:

Freq.	Numeric	Label
1,493	0	No
2,932	1	Yes, a lot
111	.	

---

time_friends — **Weekly Socialization with Friends**

```
                 type:  numeric (float)
                label:  time_friends

                range:  [0,3]                        units:  1
        unique values:  4                        missing .:  1,822/4,536

           tabulation:  Freq.   Numeric  Label
                           52         0  Never/less than 1 day
                          634         1  1-2 days
                          616         2  3-4 days
                        1,412         3  Nearly every day
                        1,822         .
```

---

friend_sex_combo — **Friend Sex Structure**

```
                 type:  numeric (float)
                label:  friend_sex_combo

                range:  [0,2]                        units:  1
        unique values:  3                        missing .:  171/4,536

           tabulation:  Freq.   Numeric  Label
                          172         0  No friends
                        1,913         1  Same-sex friends only
                        2,280         2  Any opposite-sex friends
                          171         .
```

---

ixb1ar — **Have you ever been scared or felt really bad because grown-ups called you names,**

```
                 type:  numeric (float)

                range:  [0,1]                        units:  1
        unique values:  2                        missing .:  141/4,536

           tabulation:  Freq.  Value
                        2,672  0
                        1,723  1
                          141  .
```

---

ixb1br — **Have you ever been scared that your parents or other adults were going to hurt y**

```
                 type:  numeric (float)

                range:  [0,1]                        units:  1
        unique values:  2                        missing .:  178/4,536

           tabulation:  Freq.  Value
                        3,369  0
                          989  1
                          178  .
```

---

ixb1cr — **Have you ever felt like you are not loved or cared about?**

```
                 type:  numeric (float)

                range:  [0,1]                        units:  1
        unique values:  2                        missing .:  169/4,536

           tabulation:  Freq.  Value
                        3,636  0
                          731  1
                          169  .
```

---

ixb1dr — **Have you ever felt like you have no one that protects you?**

```
                 type:  numeric (float)

                range:  [0,1]                        units:  1
        unique values:  2                        missing .:  184/4,536

           tabulation:  Freq.  Value
                        3,648  0
                          704  1
                          184  .
```

---

ixb1er — **Has there ever been a time of your life when you were totally on your own and ha**

```
                 type:  numeric (float)

                range:  [0,1]                        units:  1
        unique values:  2                        missing .:  129/4,536

           tabulation:  Freq.  Value
                        3,764  0
                          643  1
                          129  .
```

---

ixb1fr — **Have your parents/guardians ever drunk too much alcohol or used drugs so they ca**

```
                 type:  numeric (float)

                range:  [0,1]                        units:  1
        unique values:  2                        missing .:  151/4,536

           tabulation:  Freq.  Value
                        4,048  0
                          337  1
                          151  .
```

---

ixb1gr — **Has there ever been a time when your family did not have enough food because the**

```
                 type:  numeric (float)

                range:  [0,1]                        units:  1
        unique values:  2                        missing .:  112/4,536

           tabulation:  Freq.  Value
                        2,579  0
                        1,845  1
                          112  .
```

---

ixb1hr — **Have you ever seen your mom being hit, beaten or threatened?**

```
                 type:  numeric (float)

                range:  [0,1]                        units:  1
        unique values:  2                        missing .:  147/4,536

           tabulation:  Freq.  Value
                        3,501  0
                          888  1
                          147  .
```

---

ixb1ir — **Have you ever seen your mother or father so sad that they couldn't take care of**

```
                 type:  numeric (float)

                range:  [0,1]                        units:  1
        unique values:  2                        missing .:  148/4,536

           tabulation:  Freq.  Value
                        3,663  0
                          725  1
                          148  .
```

```
ixbljr                                              Have any of your parents ever been in prison/jail?

                  type:  numeric (float)

                 range:  [0,1]                        units:  1
         unique values:  2                        missing .:  154/4,536

            tabulation:  Freq.  Value
                         3,884  0
                           498  1
                           154  .

ixblkr                                   Has your family ever been forced to leave your home/house?

                  type:  numeric (float)

                 range:  [0,1]                        units:  1
         unique values:  2                        missing .:  159/4,536

            tabulation:  Freq.  Value
                         3,895  0
                           482  1
                           159  .

ixbllr                      Has an adult ever touched you in your private parts except when being bathed?

                  type:  numeric (float)

                 range:  [0,1]                        units:  1
         unique values:  2                        missing .:  104/4,536

            tabulation:  Freq.  Value
                         4,198  0
                           234  1
                           104  .

ixblmr                           Has an adult ever attempted or forced you to have sexual intercourse?

                  type:  numeric (float)

                 range:  [0,1]                        units:  1
         unique values:  2                        missing .:  88/4,536

            tabulation:  Freq.  Value
                         4,267  0
                           181  1
                            88  .

meanscore_sds                                                                        SDS mean score

                  type:  numeric (float)

                 range:  [1,5]                        units:  1.000e-07
         unique values:  43                       missing .:  5/4,536

                  mean:   4.12597
              std. dev:   1.05005

           percentiles:        10%       25%       50%       75%       90%
                               2.5   3.66667       4.5         5         5

meanscore_gst                                                                        GST mean score

                  type:  numeric (float)

                 range:  [1,5]                        units:  1.000e-07
         unique values:  59                       missing .:  7/4,536

                  mean:    4.3527
              std. dev:   .729619

           percentiles:        10%       25%       50%       75%       90%
                           3.28571         4       4.5         5         5

study_site2                                                                              Study site

                  type:  numeric (float)
                 label:  study_site2

                 range:  [1,2]                        units:  1
         unique values:  2                        missing .:  0/4,536

            tabulation:  Freq.   Numeric  Label
                         2,842         1  Kinshasa, DRC
                         1,694         2  Blantyre, Malawi

parent3                                              parental structure grandparent other together

                  type:  numeric (float)
                 label:  parent3

                 range:  [0,2]                        units:  1
         unique values:  3                        missing .:  35/4,536

            tabulation:  Freq.   Numeric  Label
                         2,207         0  both parents
                         1,747         1  single parent
                           547         2  grandparent/other
                            35         .

parent_awareness2                                                                parental awareness

                  type:  numeric (float)
                 label:  noyes

                 range:  [0,1]                        units:  1
         unique values:  2                        missing .:  0/4,536

            tabulation:  Freq.   Numeric  Label
                         1,581         0  no
                         2,955         1  yes

age_di                                                                               Age dichotomous

                  type:  numeric (float)
                 label:  age_di

                 range:  [0,1]                        units:  1
         unique values:  2                        missing .:  0/4,536

            tabulation:  Freq.   Numeric  Label
                         2,874         0  10 - 12 years
                         1,662         1  13 - 14 years

aces_cat2                                                                  ACES categorical variable

                  type:  numeric (float)
                 label:  aces_cat2

                 range:  [0,4]                        units:  1
         unique values:  5                        missing .:  102/4,536
```

```
           tabulation:  Freq.   Numeric  Label
                        1,104         0  No ACES
                          985         1  History of 1 ACES
                          806         2  History of 2 ACES
                          541         3  History of 3 ACES
                          998         4  History of 4 or more ACES
                          102         .
```

---

time_friends_everyday — **weekly socialization with friends - everyday**

```
                 type:  numeric (float)
                label:  noyes

                range:  [0,1]                        units:  1
        unique values:  2                        missing .:  1,822/4,536

           tabulation:  Freq.   Numeric  Label
                        1,302         0  no
                        1,412         1  yes
                        1,822         .
```

---

social_cohesion_overall2 — **Social cohesion overall index**

```
                 type:  numeric (float)
                label:  social_cohesion_overall2

                range:  [0,1]                        units:  1
        unique values:  2                        missing .:  199/4,536

           tabulation:  Freq.   Numeric  Label
                        1,205         0  low social cohesion
                        3,132         1  high social cohesion
                          199         .
```

---

voice_tertile_ki — **3 quantiles of meanscore_voice**

```
                 type:  numeric (byte)

                range:  [1,3]                        units:  1
        unique values:  3                        missing .:  1,695/4,536

           tabulation:  Freq.  Value
                        1,048  1
                          900  2
                          893  3
                        1,695  .
```

---

decision_tertile_ki — **3 quantiles of meanscore_decision**

```
                 type:  numeric (byte)

                range:  [1,3]                        units:  1
        unique values:  3                        missing .:  1,696/4,536

           tabulation:  Freq.  Value
                          977  1
                        1,145  2
                          718  3
                        1,696  .
```

---

fom_tertile_ki — **3 quantiles of meanscore_fom**

```
                 type:  numeric (byte)

                range:  [1,3]                        units:  1
        unique values:  3                        missing .:  1,695/4,536

           tabulation:  Freq.  Value
                        1,146  1
                          764  2
                          931  3
                        1,695  .
```

---

voice_tertile_bl — **3 quantiles of meanscore_voice**

```
                 type:  numeric (byte)

                range:  [1,3]                        units:  1
        unique values:  3                        missing .:  2,861/4,536

           tabulation:  Freq.  Value
                          599  1
                          534  2
                          542  3
                        2,861  .
```

---

decision_tertile_bl — **3 quantiles of meanscore_decision**

```
                 type:  numeric (byte)

                range:  [1,3]                        units:  1
        unique values:  3                        missing .:  2,873/4,536

           tabulation:  Freq.  Value
                          576  1
                          575  2
                          512  3
                        2,873  .
```

---

fom_tertile_bl — **3 quantiles of meanscore_fom**

```
                 type:  numeric (byte)

                range:  [1,3]                        units:  1
        unique values:  3                        missing .:  2,869/4,536

           tabulation:  Freq.  Value
                          586  1
                          593  2
                          488  3
                        2,869  .
```

---

friend_sex_combo2 — **Friend sex combo - excluding no friends**

```
                 type:  numeric (float)
                label:  friend_sex_combo

                range:  [1,2]                        units:  1
        unique values:  2                        missing .:  343/4,536

           tabulation:  Freq.   Numeric  Label
                        1,913         1  Same-sex friends only
                        2,280         2  Any opposite-sex friends
                          343         .
```

---

ixc10_12 — **violence victimization and perpetration variable**

```
                 type:  numeric (float)
                label:  ixc10_12

                range:  [0,3]                        units:  1
        unique values:  4                        missing .:  116/4,536
```

```
            tabulation:  Freq.   Numeric  Label
                         2,726         0  no victim/perpet
                           581         1  victim only
                           432         2  perpetr only
                           681         3  victim & perpetr
                           116         .
```

```
sample_complete2                                               final analytical sample

                  type:  numeric (float)

                 range:  [0,1]                        units:  1
         unique values:  2                        missing .:  0/4,536

            tabulation:  Freq.  Value
                           783  0
                         3,753  1
```

```
gst_tertile_ki                                              3 quantiles of meanscore_gst

                  type:  numeric (byte)

                 range:  [1,2]                        units:  1
         unique values:  2                        missing .:  1,695/4,536

            tabulation:  Freq.  Value
                         1,270  1
                         1,571  2
                         1,695  .
```

```
gst_tertile_bl                                              3 quantiles of meanscore_gst

                  type:  numeric (byte)

                 range:  [1,3]                        units:  1
         unique values:  3                        missing .:  2,848/4,536

            tabulation:  Freq.  Value
                           606  1
                           658  2
                           424  3
                         2,848  .

2 . save "/Users/akramaiya/OneDrive - Johns Hopkins/Data/Empowerment and Violence - DRC & Malawi/KI_MA_combined_PLoSMed.dta", replace
  file /Users/akramaiya/OneDrive - Johns Hopkins/Data/Empowerment and Violence - DRC & Malawi/KI_MA_combined_PLoSMed.dta saved

3 . log close
        name:  <unnamed>
         log:  /Users/akramaiya/Dropbox/Personal Work/GEAS/GEAS Empowerment Working Group/Empowerment and Violence Perpetration & Victimization/PLoS Medicine
  > /Revise and Resubmit - 3/codebook.smcl
    log type:  smcl
   closed on:  19 Nov 2021, 16:42:07
```
