# Supplementary material for: Assessing the relationship between agency and peer violence among adolescents aged 10 to 14 years in Kinshasa, Democratic Republic of Congo and Blantyre, Malawi: A cross-sectional study
Source: PLoS Med. 2021 Dec 13;18(12):e1003552. doi: 10.1371/journal.pmed.1003552 (PMC8716028; doi:10.1371/journal.pmed.1003552)
Supplement: Attachment — Submitted filename: PLOS Medicine peer review.docx [file pmed.1003552.s011.docx]

**Review of *Assessing the Relationship Between Agency and Peer-violence among Very Young Adolescents in Two African Settings: A Cross-Sectional Study***

Overall, this is an interesting and competently written paper focusing on an important and often neglected age cohort – very young adolescents – in two urban populations in SSA. It makes the important argument that sex differences in the perpetration or victimisation of peer violence vary across context and to support interventions more precise understanding and operationalisation of individual adolescent empowerment is required.

In some ways, however, the article raises more questions than it answers and either additional analysis should be undertaken to address these or the limitations section expanded.

First, it would be good if the article could more clearly state whether peer violence is referring to violence experiences with young people only of the same sex or among girls and boys, and if so what proportion. For example, is the analysis of girls’ experience of peer violence capturing violence by girls against girls or by boys or both and what is the balance of this experience. This matters in terms of the sorts of sensitisation and monitoring required.

Second, in what spaces is the violence occurring. The paper makes the argument that greater freedom of movement permitted of boys means that they are more exposed to peer violence experiences – but where is the violence happening? At school or in the community? And do we see differences in the Kinshasa sample based on school enrolment status?

Third, there are number of areas where the reader wants to know why. With only survey data –rather than mixed methods data (although my understanding is that GEAS does also collect qualitative data) – it is difficult to get purchase on for example why were sex differences in the perpetration or victimisation alone negligible? Why did girls with greater voice also find themselves still at risk of victimisation by peers –was this due to inter-personal communication skills (e.g. limited negotiation skills, gender norms that de-value girls’ voices, participation in programming that focused on young people’s empowerment but did not foster change in the wider enabling environment), gender-based harassment and violence, and so forth? If it is not possible to draw on qualitative data to triangulate the findings for this paper, then it would be good to add this to the limitations section.

Fourth, given the sensitivity of asking about experiences of violence, it would be important for the authors to reflect more on possible limitations of different survey data collection methods (i.e. with trained enumerators in Kinshasa vs self-reported in Blantyre). Could this potentially explain some of the level of difference across country?

Fifth, given that in Kinshasa there are both in-school and out-of-school adolescents, to what extent does this explain differences across country? Could the data be disaggregated and bar graphs added in to show the findings for these different samples? If not, it needs to be explicitly discussed in the limitations. Also the descriptive statistics show that there is a difference among those who at-age-for-grade and those who are over-age-for-grade - can the data be disaggregated to explore possible differences in findings among these sub-groups?

Finally, the discussion on implications for programming are quite general and there is a need for more nuanced discussion on how the proposed recommendations could be realistically achieved in such contexts. For example, “Second, while empowering young people is a critical component of positive youth development, promoting individual agency may also have unforeseen consequences, which should be anticipated and addressed to reduce violence experiences”. As a reader I would like to know how this could be achieved and whether there are any examples of promising practice in this regard. Similarly, for this recommendation “Finally, the site differences in associations according to peer, family and neighborhood-context stress the need to ground interventions in the social context of young people’s lives.” - what does this mean in practice – more tailored case management?
