# Supplementary material for: Assessing the relationship between agency and peer violence among adolescents aged 10 to 14 years in Kinshasa, Democratic Republic of Congo and Blantyre, Malawi: A cross-sectional study
Source: PLoS Med. 2021 Dec 13;18(12):e1003552. doi: 10.1371/journal.pmed.1003552 (PMC8716028; doi:10.1371/journal.pmed.1003552)
Supplement: Attachment — Submitted filename: Response to Reviewers.docx [file pmed.1003552.s012.docx]

**Editor comments:**

1. Data availability statement: Thank you for noting the specific restrictions for access of your data.  Please revise your statement, to include appropriate information allowing interested parties to access your study data (a dataset reference or accession number, a web link, and/or contact email addresses).  Please note that the authors of the study (Eric Mafuta and William Stones) may not serve as contact points for providing data access.-

Response: The data are now available as supplement file 7.

2. When submitting your revised manuscript, please provide line numbers throughout the document.

Response: The revised document has continuous line numbers.

3. Abstract: Please structure your abstract using the PLOS Medicine headings (Background, Methods and Findings, Conclusions; please combine methods and results into a single section “Methods and Findings”).

Response: We have restructured the abstract per PLOS Medicine guidelines. Methods and Findings section has been combined in the abstract (Page 2 - 3)

4. Abstract: Background: Please indicate Blantyre, Malawi

Response: Thank you. We added Malawi after Blantyre (Line 36).

5. Abstract: Methods and Findings: Please quantify the main results (with 95% CIs and p values).

Response: We have added the 95% CI and p-values in the Findings:

“Adolescents reporting the greatest (tertile 3) freedom of movement (with the exception of girls in Kinshasa) were more likely to experience an perpetrator-victim overlap (Boys Kinshasa: 1.9 (1.2 – 1.8, p<0.05), Boys Blantyre: 3.8 (1.7 – 8.3, p<0.01), Girls Blantyre: 2.4 (1.1 – 5.1, p<0.05)). Highest decision making power (Kinshasa boys: 3.0 (1.8 – 4.8, p<0.001); Kinshasa girls: 1.7 (1.02 – 2.7, p<0.05) was associated with the perpetrator-victim overlap. Higher voice among boys in Kinshasa (T2: 1.9 (1.2 – 2.9, p<0.01); T3: 1.8 (1.2 – 2.8, p<0.01)) and girls in Blantyre (T2: 2.0 (1.01 – 3.9, p<0.05)) was also related to the perpetrator-victim overlap, but associations differed for victimization only by sex and site. In Kinshasa, boys with more decision-making power were less likely to be victimized (T2: 0.6 (0.4 – 0.9, p<0.01)) but not girls. Girls with more voice in Blantyre were more likely to be victimized (T2: 1.9 (1.1 – 3.1, p<0.05)) but this relationship was not true for girls in Kinshasa. ” (Page 3)

6. Abstract: Methods and Findings: In the last sentence of the Abstract Methods and Findings section, please describe the main limitation(s) of the study's methodology.

Response: Thank you. We have added the following limitation “However, due to the cross-sectional nature of the study, causality between agency and peer-violence cannot be established.” (Line 63-65)

7. Abstract: Conclusions: In the first sentence please address the specific implications of your study based on the results; the phrase "In this study, we observed ..." may be useful (Line 66-69).

Response: We have amended the conclusions based on the reviewer recommendation.

In this study we observed that peer-violence experiences were gendered in early adolescence in relation to adolescent agency. This stresses the importance of preventing interpersonal violence and including boys in programs designed to empower young people and to promote gender equality. (Line 66-69)

8. Author summary: At this stage, we ask that you include a short, non-technical Author Summary of your research to make findings accessible to a wide audience that includes both scientists and non-scientists. The Author Summary should immediately follow the Abstract in your revised manuscript. This text is subject to editorial change and should be distinct from the scientific abstract. Please see our author guidelines for more information: [https://nam02.safelinks.protection.outlook.com/?url=https%3A%2F%2Fjournals.plos.org%2Fplosmedicine%2Fs%2Frevising-your-manuscript%23loc-author-summary&amp;data=04%7C01%7Caramaiy1%40jhu.edu%7C7c9ef925b7674f02b8b608d8f5dc6de9%7C9fa4f438b1e6473b803f86f8aedf0dec%7C0%7C0%7C637529674376783959%7CUnknown%7CTWFpbGZsb3d8eyJWIjoiMC4wLjAwMDAiLCJQIjoiV2luMzIiLCJBTiI6Ik1haWwiLCJXVCI6Mn0%3D%7C1000&amp;sdata=gkQYIayu%2FEQdqGn2kFnxWQTdAfCgQ3x%2Bbc0jW%2BEufpE%3D&amp;reserved=0](https://nam02.safelinks.protection.outlook.com/?url=https%3A%2F%2Fjournals.plos.org%2Fplosmedicine%2Fs%2Frevising-your-manuscript%23loc-author-summary&data=04%7C01%7Caramaiy1%40jhu.edu%7C08bf522425304f1cbe5b08d8f5defbec%7C9fa4f438b1e6473b803f86f8aedf0dec%7C0%7C0%7C637529685343048557%7CUnknown%7CTWFpbGZsb3d8eyJWIjoiMC4wLjAwMDAiLCJQIjoiV2luMzIiLCJBTiI6Ik1haWwiLCJXVCI6Mn0%3D%7C1000&sdata=KF2J2sFPwmw8dprELFxdqgk8VSMbcMe3f7u3w%2FWG4Gk%3D&reserved=0)

Response: We have included an author summary after the abstract (Page 4 – 5 )

9. Throughout the text: Please use square brackets for in-text citations, like this [1].

Response: We have changed the references to square brackets.

10. Methods: Reporting Checklist: Please ensure that the study is reported according to the STROBE guideline, and include the completed STROBE checklist as Supporting Information.  When completing the checklist, please use section and paragraph numbers, rather than page numbers.  Please add the following statement, or similar, to the Methods: "This study is reported as per the Strengthening the Reporting of Observational Studies in Epidemiology (STROBE) guideline (S1 Checklist)."

Response: We have included the STROBE checklist as Supplement file 1 and acknowledge the use of STROBE as guiding the study report “This study is reported as per the Strengthening the Reporting of Observational Studies in Epidemiology (STROBE) guideline (Supplement 1)”

(line 167-168)


11. Methods: Analysis Plan: Did your study have a prospective protocol or analysis plan? Please state this (either way) early in the Methods section.

a) If a prospective analysis plan (from your funding proposal, IRB or other ethics committee submission, study protocol, or other planning document written before analyzing the data) was used in designing the study, please include the relevant prospectively written document with your revised manuscript as a Supporting Information file to be published alongside your study, and cite it in the Methods section. A legend for this file should be included at the end of your manuscript.

b) If no such document exists, please make sure that the Methods section transparently describes when analyses were planned, and when/why any data-driven changes to analyses took place. - NA

c) In either case, changes in the analysis-- including those made in response to peer review comments-- should be identified as such in the Methods section of the paper, with rationale. – NA

Response: We have now specified that the study has a prospective protocol (line 170-171). Supplement 2 and 3 provide the IRB protocol for Kinshasa and Blantyre, respectively. We have also included this description at the end of the manuscript


12. Methods: Page 7: Please remove the trademark symbol from SurveyCTO®

Response: We have removed the trademark symbol (Line 201)

13. Methods: Page 7: Please provide more detail on whether the description here applies to both child assent and parental consent, or whether these differed

Response: We have specified that the oral consent applies to both parent and adolescent in Kinshasa and written consent applies to parent and adolescent in Blantyre (Line 203-204)

14. Methods: Please include the survey questions used to assess these three agency dimensions, and all covariates, as a supporting information file.

Response – We have added a line in the Measure section to indicate that “Supplement File 4 outlines the questions and responses used from the GEAS instrument to answer the research question in this manuscript. (line 207-208))

15. Methods: Page 9: Please clarify the sentence: “We first examined the incidence of violence perpetration and victimization in the last § months…”

Response: Thank you for noting this error. We corrected the error (“in the last 6 months”). (Line 253)

16. Results: Please quantify results presented in the text as much as possible.  On page 14, please present the risk ratio in the text along with the results from the multivariate analysis (95% CI and p values) for the findings discussed.  Please clarify where differences between sites or sexes were quantified, as opposed to where differences are apparent at the qualitative level.

Response: We have now added sample sizes and p-values to the univariate and bivariate tables (Table 1 and Table 2). Additionally, we describe p relative risk ratios, 95% CI and p-values. In the text the differences between sex within each site were quantified, however, significance testing of the differences between sites are not included in the tables, because the sampling and data collection modes are different in the two sites; contributing to the differences observed. Nevertheless, we have added the following sentence in the result section “While differences by site may reflect methodological differences limiting the interpretation of site interactions, we note significant differences in the associations between agency and violence by site for boys, which generally reflect greater positive associations between freedom of movement and violence and to a lesser extent between decision making and violence victimization in Blantyre compared to Kinshasa (interaction between site and freedom of movement: p=0.002; interaction between site and decision making: p=0.05) Fewer significant differences by site were noted for girls, with the exception of voice which was associated with reduced violence perpetration and victimization in Kinshasa but increase perpetration and victimization in Blantyre (test of interaction, p=0.05) (Line 341 - 350)

17. Discussion: Please slightly re-organize the Discussion as follows: a short, clear summary of the article's findings; what the study adds to existing research and where and why the results may differ from previous research; strengths and limitations of the study; implications and next steps for research, clinical practice, and/or public policy; one-paragraph conclusion.

Response: Thank you, we have now structured the discussion in the way you proposed (page 24 – 28)

18. Discussion and throughout: It is somewhat confusing to switch back and forth between “gender” and “sex” (even within a single sentence at the top of page 18).  I understand the point you make on page 9 that sex is the relevant term in your study as you did not collect data on gender identity, and that your survey contains gender-related measures.  Please make sure the two terms (which have different meaning) are used as consistently and clearly as possible throughout.

Response: Thank you for pointing this out. We have now tried to standardize the use of “sex” in the introduction and discussion.

19. Discussion: In the limitations, please fully discuss limitations of the study design, including selection bias brought up by one reviewer, as well as issues related to residual confounding.

Response: Thank you. We have now included a statement in the limitations which outlines selection bias as a limitation. (page 26-27)

“There is additionally the potential for residual confounding due to unmeasured confounders, particularly as we investigated individual components of adolescent agency but were unable to assess the role of collective empowerment, a critical component of violence prevention programs in preventing teen violence.” (line 433-436)

“Selection bias may also have impacted our findings, particularly if adolescents who are victims of violence or who have limited agency are less likely to be identified and enrolled in the study. If our study differentially selected participants who were less likely to experience violence, it is likely that our findings are conservative” .(line 439-442)

20. Table 1: Please provide the number for each percentage given (even though the total is given at the top of the columns).  Please define the abbreviation ACES, SD, in the legend

Response: We have added the sample size for each percentage provided and included p-values in Table 1. I have also spelled out ACES and SD (table 1 and line 298 – 301)

21. Figure 1: Please consider that this may be more clearly presented as a table.  Please also provide the number in addition to the percentages for each value.

Response: We have removed the figure and included this information in table 1, as suggested (table 1, page 17)

22. Table 2: Please provide the numbers for each percentage.  Please report the full statistics for each comparison, along with the 95% CIs and p values, rather than noting significance with asterisks.

Response: we have included the proportions, 95% CI, sample sizes and p-values in Table 2, as suggested (table 2, page 18 – 19)

23. Table 3: Please report the full p values for each comparison, rather than indicating significance levels with asterisks

Response: We have added p-values) (table 3, page 22-23)

24. References:   Please use the "Vancouver" style for reference formatting, and see our website for other reference guidelines [https://nam02.safelinks.protection.outlook.com/?url=https%3A%2F%2Fjournals.plos.org%2Fplosmedicine%2Fs%2Fsubmission-guidelines%23loc-references&amp;data=04%7C01%7Caramaiy1%40jhu.edu%7C7c9ef925b7674f02b8b608d8f5dc6de9%7C9fa4f438b1e6473b803f86f8aedf0dec%7C0%7C0%7C637529674376783959%7CUnknown%7CTWFpbGZsb3d8eyJWIjoiMC4wLjAwMDAiLCJQIjoiV2luMzIiLCJBTiI6Ik1haWwiLCJXVCI6Mn0%3D%7C1000&amp;sdata=vEAEe7yLkQ%2FPrCpE2vSp%2Bqiq9k6zKphH74%2FwRgzdxwk%3D&amp;reserved=0](https://nam02.safelinks.protection.outlook.com/?url=https%3A%2F%2Fjournals.plos.org%2Fplosmedicine%2Fs%2Fsubmission-guidelines%23loc-references&data=04%7C01%7Caramaiy1%40jhu.edu%7C08bf522425304f1cbe5b08d8f5defbec%7C9fa4f438b1e6473b803f86f8aedf0dec%7C0%7C0%7C637529685343058552%7CUnknown%7CTWFpbGZsb3d8eyJWIjoiMC4wLjAwMDAiLCJQIjoiV2luMzIiLCJBTiI6Ik1haWwiLCJXVCI6Mn0%3D%7C1000&sdata=MNJ5isAXZUGdSqmCQnLBSGloGGKC%2B2tijHHKDYZhQio%3D&reserved=0) –

Response: Thank you for clarifying the preferred reference style. We have now used Vancouver reference style with square brackets.

25. Appendix 1 and Appendix 2: Please provide the actual p values associated with the results presented, rather than levels of significance.  In the legend, please briefly explain why parts of the table are grayed out.  We suggest renaming these files as “S1_Table” and “S2_Table” respectively.

Response: The grayed out boxes are covariates which were not relevant in that site or were not collected. We have included this in the legend for S5_Table and S6_table. We have also included p-values in the Tables (page 38 – 41)

**Reviewer 1:**

This paper addresses the important topic of interpersonal violence among adolescents in Kinshasa and Blantyre. Overall, the study suffers from several limitations related to its cross sectional design, including the potential for reverse causation and selection bias. Moreover, the target population for the study is not explicitly stated, and the method used to sample, invite, and recruit participants was not clear. Therefore, it is difficult to understand to whom results are expected to apply. General and specific comments are provided below.

General comments:

1.      The paper does not describe how participants were selected for inclusion. For example, how were the 2016 in school and 826 out of school adolescents in Kinshasa selected? How many were invited to participate? What was the refusal rate?

Response: We have extended the paragraph describing sample selection (Participants from Kinshasa included 2,016 adolescents from 80 schools and 826 out of school adolescents, due to high levels of school dropout at an early age in Kinshasa. In-School and out of School adolescents were selected from two poor disadvantaged communes. In-school adolescents were selected from 80 public and catholic schools serving the poorest children in the 2 communes, half of whom participated in the Growing Up Great intervention, conducted in the year following baseline GEAS data collection. A total of 25 adolescents per school were selected, stratified by age and sex. Out of school were selected in the same communes, based on a listing of households including out of school adolescents, created by Community Based Organizations. The list was further refined to identify adolescents 10-14 who had left school more than two years before the survey and were not expecting to resume school in the following year, while continuing to reside in the neighbourhood. Adolescents were randomly selected from this list, after stratification by neighbourhood, sex and age. All participants who were approached completed the survey. In Blantyre, the sampling frame was 6th grade students from six schools. A total of 2,140 adolescents were approached and1,694 (79%) completed the survey. In Blantyre, data collection was suspended due to COVID-19 related school shutdown, hence a large number of participants were approached but not included in the final sample (21%) (page 9 – 10)

2.      Are there circumstances in which experiences of violence would affect participation in the survey? Certainly, this could be true in the unfortunate event that violence led to death. But violence could also affect school drop out, which could affect inclusion in the study in Malawi, and may affect recruitment in other ways. Moreover, agency (freedom of movement, voice, and decision-making) could also affect inclusion in the study. In settings where both exposure and outcome affect selection, the overall prevalence of reported outcomes as well as the associations between exposure and outcome could be distorted. This potential selection bias should be discussed.

Response: Thank you, we have now included a discussion of the potential impact of selection bias as a limitation in the discussion (page 26). Though we acknowledge that victims of violence may have a higher risk of mortality, mortality in early adolescence is considered a rare event. For this reason, we believe it is unlikely that a sufficient number of adolescents who would otherwise have been eligible for our study would have been killed to significantly affect our findings. However, we agree that adolescents who experience violence victimization may have been less likely to enroll in the study, in which case we believe our findings are likely to be conservative.

“There is additionally the potential for residual confounding due to unmeasured confounders and potential for selection bias, particularly if adolescents who are victims of violence or who have limited agency are less likely to be identified and enroll in the study. If our study differentially selected participants who were less likely to experience violence, it is likely that our findings are conservative. (line 439-441)

3.      The authors should comment on whether they believe that the difference in data collection techniques (ACASI vs in person interviews) could result in differences in results between the two countries.

Response: Thank you. We have included that in the limitation section (line 442 - 450).

Differences in data collection modalities between sites (in-person in Kinshasa and Audio-Computer Assisted Self Interview in Blantyre), may generate differences in the patterns of responses resulting from modality rather than true differences between sites. This is especially notable in Blantyre which had a larger number of missing dependent, independent, and adjustment covariates compared to Kinshasa, where social desirability bias may have impacted responses. As all participants within each site completed the survey using the same modality, it is not possible to disentangle the effect of the modality though we treat each site as an effect modifier in analyses to account for cross-site differences, including data collection modality.

4.      The Methods section should clearly state the parameters of interest that will be interpreted in the Results section.

Response: Thank you for this comment, however we are unclear on specifically what is being requested. We describe the definition and creation of the outcome variable in lines 209 - 219 and the independent variables of interest in lines 220 - 231. We provide additional information on covariates in lines 232-249. We have also now provide a supplemental table S4_Table describing the questions and their response options. If the reviewer is able to provide additional clarification on this request, we will modify this section accordingly.

5.      The paper acknowledges the limitations of working with cross sectional data in the Discussion section. However, more discussion is needed on the potential for reverse causation (i.e., in which violence affects the agency measures) and what that might mean for the interpretation of the results.

Response: We have included a sentence acknowledging the potential for reverse causality in the limitations section line 425 - 433:

“The cross-sectional design of this study limits our interpretation of the perpetrator-victim overlap, in particular determining the directionality of the associations described (reverse causality). Although we assume that participants with more agency are more prone to peer-violence, it could be that those who are engaging in peer-violence have more agency. While our results demonstrate that agency and peer-violence are associated, we anticipate that the longitudinal data currently collected from the GEAS will provide further insights on the dynamics of peer violence experiences (from victimization to perpetration and vice versa) as well as the predictive effects of young people’s agency on subsequent violence outcomes.

6.      The items used to construct measures of agency should be described either in the text or supplementary materials.

Response: Thank you. We have outlined the questions used to construct agency in the S4_Table.

| **Freedom of Movement Score: Can you tell me how often you are allowed to do the following alone (without an adult present)?** |  |
| --- | --- |
| Go to after-school activities (like sports clubs) | Often |
| Go to a party with boys and girls | Sometimes |
| Meet with friends after school | Rarely |
| Go to community center/movies/youth center | Never |
| Visit a friend of the opposite sex | Refuse |
| **Voice Score: How often are the following statements true for you?** | Don't Know |
| My parents or guardians ask for my opinion on things |  |
| My parents or guardians listen when I share my opinion |  |
| My friends ask my advice when they have a problem |  |
| If I see something wrong in school or the neighborhood I feel I can tell someone and they will listen |  |
| I can speak up in class when I have a comment or question |  |
| I can speak up when I see someone else being hurt |  |
| I can ask adults for help when I need it |  |
| **Decision Score : How often are you able to make the following decisions on your own, without an adult?** |  |
| What clothes to wear when you are not in school/working |  |
| What to do in your free time |  |
| What to eat when you are not at home |  |
| Who you can have as friends |  |

Specific Comments:
1.      Abstract: the first sentence of the methods states that the surveys were conducted in 2017 and 2020, yet the methods section states that data collection occurred between 2017 and 2020. I recommend providing more detail on specific dates of data collection in each site.

Response: Thank you we have clarified as below:

“This study draws on two cross-sectional surveys including 2,540 adolescents 10-14 years in Kinshasa in 2017 and 1,213 in Blantyre in 2020” (line 38)

2.      Page 9, last paragraph: the manuscript uses a symbol I don't understand here. What was the lookback period for incidence of violence?

Response: Thank you for highlighting this error. We have changed this to 6 months (line 253)

3.      Table 2: I appreciate that the paper shows absolute percentages experiencing each level of violence by tertile. However, because there are many categories, it is unclear what hypothesis the p-value is testing and what test was used to obtain the p-value. These points should be clarified if p-values are reported.

Response: The p-value being tested is the three tertiles of agency with four levels of the peer-violence experience. We have expanded on this in the methods section: “We then tested for differences in the distribution the the 4 categorical measure of peer-violence by tertile of each agency measure, using Chi-square tests.” (line 254-256)

4.      Page 13, first sentence: Table 2 appears to show absolute percentages of each tertile in each category of the outcome, not associations directly.

Response: Thank you. We have changed the first sentence to “ Table 2 shows the proportion of peer-violence experience according to an adolescent’s level of agency (in tertiles).” (line 303-304)

5.      Page 14, first sentence: it is unclear to me what the term "relative risk ratio" represents here. Presumably the results of Table 3 come from the multiviariable multinomial logistic regression model mentioned in the Methods. However, that method would provide odds ratios, rather than risk ratios. If the "relative risk ratios" are calculated using some other approach, it should be detailed in the Methods section.

Response: We used multinomial logistic regression rather than a binomial logistic regression. In the multinomial model, exp(β) is not a comparison of two groups (Y=1 versus Y=0). The stata manual calls it a “relative risk ratio”. Since we used Stata for the analysis, we are using RRR based on the manual’s suggestion (Stata Base Reference Manual n.d.). However, we understand the challenges of interpreting RRRs when the proportion of the category is not low.

6.      Moreover, to avoid confusion with "multivariate" models (typically used when more than one outcome is of interest), I would refer to the analysis in table 3 as the "adjusted analysis" rather than "multivariate analysis".

Response: Thank you, we have changed the multivariate analysis to adjusted analyses (line 323).

7.      Page 14, second paragraph: the paper appears to commit the Table 2 fallacy, in which a multivariable model was used to assess, and interpret, relationships between all variables included in the model and the outcome. However, using results from a single multivariable model, one should not interpret all of the resulting regression coefficients because many of the characteristics affect, and are affected by, each other. This means that the interpretation of some resulting regression coefficients (or odds ratios) would be as "total effects" and others would be "direct effects after holding mediators constant" This phenomenon is nicely described in the reference below. –

Daniel Westreich, Sander Greenland; The Table 2 Fallacy: Presenting and Interpreting Confounder and Modifier Coefficients, American Journal of Epidemiology, Volume 177, Issue 4, 15 February 2013, Pages 292-298, <https://doi.org/10.1093/aje/kws412>

Response: Thank you for the comment and the useful reference. As noted, we did one multivariable regression for each site and sex which included the dependent variable (peer-violence experiences), independent variables (freedom of movement, voice and decision making), individual factors, family factors, peer factors and community factors. Table 3 shows the adjusted effect of the agency variables on peer-violence. S5_Table and S6_Table show the RRR of the confounding variables.

8.      Table 3, in the methods section and/or the footnote of the table, please state what p-values are comparing and how they were obtained.

Response: The p values assess the difference in violence victimization versus non violence experience according to each agency tertile (medium or higher) relative to the lowest agency tertile. These associations are evaluated separately among Kinshasa boys, Kinshasa girls, Blantyre boys and Blantyre girls, and adjusting for all other covariates (individual, family, peer and community factors). We have outlined this in the methods section (line 256 - 259) and as a footnote in Table 3.

9.      Figure 1 was a bit difficult to read, I recommend larger text.

Response: Per editorial feedback, we have incorporated Figure 1 into table 1 (table 1) .

**Reviewer #2:**

Overall, this is an interesting and competently written paper focusing on an important and often neglected age cohort - very young adolescents - in two urban populations in SSA. It makes the important argument that sex differences in the perpetration or victimisation of peer violence vary across context and to support interventions more precise understanding and operationalisation of individual adolescent empowerment is required..

In some ways, however, the article raises more questions than it answers and either additional analysis should be undertaken to address these or the limitations section expanded.

First, it would be good if the article could more clearly state whether peer violence is referring to violence experiences with young people only of the same sex or among girls and boys, and if so what proportion. For example, is the analysis of girls' experience of peer violence capturing violence by girls against girls or by boys or both and what is the balance of this experience. This matters in terms of the sorts of sensitisation and monitoring required.

Response:. We appreciate the reviewer’s comments and agree that there is more nuance in violence victimization and perpetration than we have presented here. The Table below presents distributions of violence experiences by sex of protagonists and we have summarized this information in the results section.

“Peer violence mostly involved same sex peers in Kinshasa (60% to 64%, depending on victimization versus perpetration), while experiences were more diversified in Blantyre with 39% to 44% involving same sex peers, 29 to 45% involving opposite sex peers and 21 to 24% involving both sexes.” (Line 290 – 294)

Given the small sample sizes of iterative categories (site, sex, type of violence, sex of victim/perpetrator), we felt the paper was stronger when we were able to maintain sufficient sample size to detect differences.

We have noted the issue of sample size in the method section “While the GEAS study collected information about the sex of the protagonist, we did not include this information in the indicator due to small sample size concerns. However, we report the distribution of violence according to sex of respondent and protagonist in the result section.” (Line 216 - 219)

|  | Kinshasa, DRC | | Blantyre, Malawi | |
| --- | --- | --- | --- | --- |
| **Victimized in the last 6 months** | **Boys (n)** | **Girls (n)** | **Boys (n)** | **Girls (n)** |
| *No* | 71.0% (904) | 76.9% (973) | 69.7% (417) | 79.0% (486) |
| *Yes, by both boys and girls* | 6.7% (85) | 2.8% (35) | 6.5% (39) | 5.7% (35) |
| *Yes, by a boy or boys* | 17.4% (222) | 6.5% (81) | 11.7% (70) | 6.0% (37) |
| *Yes by a girl or girls* | 5.0% (63) | 14.0% (177) | 12.0% (72) | 9.3% (57) |
| **Perpetrated in the last 6 months** | **Boys (n)** | **Girls (n)** | **Boys (n)** | **Girls (n)** |
| *No* | 70.8% (903) | 80.3% (1016) | 57.2% (342) | 67.3% (414) |
| *Yes, by both boys and girls* | 5.7% (73) | 1.4% (18) | 6.9% (41) | 7.8% (48) |
| *Yes, by a boy or boys* | 18.8% (240) | 6.2% (79) | 16.1% (96) | 12.5% (77) |
| *Yes by a girl or girls* | 4.6% (58) | 12.1% (153) | 19.9% (119) | 12.4% (76) |

Second, in what spaces is the violence occurring. The paper makes the argument that greater freedom of movement permitted of boys means that they are more exposed to peer violence experiences - but where is the violence happening? At school or in the community? And do we see differences in the Kinshasa sample based on school enrolment status?

Response: We agree that this is a compelling question and important to understand in order to design appropriate responses. Unfortunately, we do not collect information about where violence is occurring. We suggest future work should specify the circumstances in which peer violence occurs to guide programmatic response in the discussion line 450 - 454.

“. Fourth, we have little information about the context in which peer violence occurs, which is not only programmatically relevant for violence prevention, but may also inform the relationship between agency and peer violence. Future qualitative work could shed more light on the ways agency and violence interconnect, according to social context..”

Third, there are number of areas where the reader wants to know why. With only survey data -rather than mixed methods data (although my understanding is that GEAS does also collect qualitative data) - it is difficult to get purchase on for example why were sex differences in the perpetration or victimisation alone negligible? Why did girls with greater voice also find themselves still at risk of victimisation by peers -was this due to inter-personal communication skills (e.g. limited  negotiation skills, gender norms that de-value girls' voices, participation in programming that focused on young people's empowerment but did not foster change in the wider enabling environment), gender-based harassment and violence, and so forth? If it is not possible to draw on qualitative data to triangulate the findings for this paper, then it would be good to add this to the limitations section.

Response: We agree that qualitative data collection would be a valuable source of data to further contextualize and explain our findings. Unfortunately, GEAS Phase 2, from which these data are drawn. did not include qualitative data collection. We have noted that future research should consider exploration of these findings via qualitative means (line 453-454).

Fourth, given the sensitivity of asking about experiences of violence, it would be important for the authors to reflect more on possible limitations of different survey data collection methods (i.e. with trained enumerators in Kinshasa vs self-reported in Blantyre). Could this potentially explain some of the level of difference across country?

Response: Thank you for the comment. Differences in interview mode may result in differential reporting of violence experiences, especially violence perpetration. On the one hand, there is more missing data in the self-administered Blantyre survey, while face to face interviews may lead to underreporting of violent behaviors due to social desirability bias. We partially account for differences in survey mode by stratifying the analysis by site, assuming survey mode contributes to differences in violence prevalence. We acknowledge this limitation in the discussion section (line 442 - 447)

Due to the difference in data collection modalities between sites (in-person in Kinshasa and Audio-Computer Assisted Self Interview in Blantyre), there could be differences in the patterns of responses resulting from modality rather than true differences between sites. This is especially notable in Blantyre which had a larger number of missing dependent, independent, and adjustment covariates compared to Kinshasa, where social desirability bias may have impacted responses. As all participants within each site completed the survey using the same modality, it is not possible to disentangle the effect of the modality though we treat each site as an effect modifier in analyses to account for cross-site differences, including data collection modality.. “Third, due to the difference in data collection modalities between sites (in-person in Kinshasa and Audio-Computer Assisted Self Interview in Blantyre), there could be differences in the results (this is especially notable with Blantyre having more missing data in comparison to Kinshasa; however in Kinshasa, face to face interviews may increase social desirability bias, leading to the underreporting of violence perpetration. We have treated the sites as effect modifiers, and therefore account for differences in data collection modalities in the analysis.”

Fifth, given that in Kinshasa there are both in-school and out-of-school adolescents, to what extent does this explain differences across country? Could the data be dis-aggregated and bar graphs added in to show the findings for these different samples? If not, it needs to be explicitly discussed in the limitations. Also the descriptive statistics show that there is a difference among those who at-age-for-grade and those who are over-age-for-grade  - can the data be dis-aggregated to explore possible differences in findings among these sub-groups?

Response: Thank you for this comment. We have examined the association between agency and violence among in school and out of school adolescents and tested for potential differences of effect between these two populations. We found no significant interactions and therefore combined the samples, to increase statistical power (line 271-273). In addition, differences between sites remained significant after excluding the out of school from the sample. We provide this information in the results (line 341-350)

Finally, the discussion on implications for programming are quite general and there is a need for more nuanced discussion on how the proposed recommendations could be realistically achieved in such contexts. For example, "Second, while empowering young people is a critical component of positive youth development, promoting individual agency may also have unforeseen consequences, which should be anticipated and addressed to reduce violence experiences". As a reader I would like to know how this could be achieved and whether there are any examples of promising practice in this regard. Similarly, for this recommendation "Finally, the site differences in associations according to peer, family and neighborhood-context stress the need to ground interventions in the social context of young people's lives."  - what does this mean in practice - more tailored case management?

Response: Thank you.We have changed this paragraph to include more specific recommendations (line 463-467):

First, programs that promote young people’s agency need to conjointly address gender norms and violence as normative expectations may lead those who gain power towards increased aggression as a means of assertion and self-protection. Second, the increase in violence perpetration and victimization among adolescents who have experienced ACES as well as the extent of a perpetration and victimization overlap reaffirms the importance of a trauma informed care to prevent teen violence. Third, strategies to prevent youth violence need to engage the broader social environment, starting with the family and extending to communities, to reduce violence exposure and perpetration. For instance, while many parents recognize the risk of violence for their sons, many fail to act considering sons need to rely on their own agency to protect themselves [22] The role of the social context, illustrated in the differences in association by site, also stresses the importance of including young people and their social networks in the design and implementation of interventions in order to better address the social and structural drivers of teen violence .Training community members would ensure that interventions are trusted, followed and sustained.

With these revisions i am of the view that this paper can make a strong contribution to the literature on adolescent peer violence.

**Reviewer #3:**

1.      Summary

This paper uses cross-sectional data on very young adolescents from two urban cities (Kinshasa (DRC) and Blantyre (Malawi) to assess the association between agency (operationalized with three scales: freedom from movement, voice, and decision making) and peer violence (defined as four categorical variables: no victimization/perpetration, victimization, perpetration, both victimization and perpetration).  Analyzing results by sex and site, the authors find that adolescents reporting greater freedom of movement were more likely to experience perpetrator-victim overlap, with more complicated associations for decision making and voice.

2.      Major Comments

The paper was very interesting to read, and I appreciate the focus on defining peer violence looking at both victimization and perpetration (and the combination) with a gender lens. It also highlights the complex relationship between agency and peer violence and provides food for thought on the possible adverse consequences of programs that promote agency, without considering the environments adolescents live in.

Response: Thank you.

I do think the authors could do more to motivate the research question, ensure the empirical analysis reflects the complexity laid out in the motivation, and to build more on the programmatic implications.  Some specific suggestions below.

a.      I am still not sure what to make of the research question.  The introduction does a good job motivating the issue of peer violence and the issue of agency, but less on the association between the two. Why would we expect a relationship between the two in LMICs? And why is this likely to be gendered?  What evidence do we already have on this association? If agency puts you at risk of exposure to violence, what does this mean for policy etc.?  This comes out a bit clearer in the discussion, but I think the introduction could provide clearer motivation of the evidence base and what this paper hopes to add.

Response: Previous studies in low and middle income countries have shown a relationship between autonomy and interpersonal violence (IPV), Whereas economic dependence and lack of negotiation skills on a partner increases IPV, greater autonomy also increases IPV due to the perceptions of male hegemony in a relationship (Page 7, line 135 - 144)

b.      The research question is motivated within a socioecological model, and I am assuming Table 2 controls for all the variables that are in the appendix tables, is that correct?  It would be great to unpack this relationship more, and perhaps explore moderators/mediators more explicitly. I personally think those appendix tables are important and would add value to the main text.    One way to build on what is here is instead to show Table 1 as a regression (with no additional controls) and then add controls at different levels.  Theory/existing evidence may also suggest the possibility of interacting the measures of agency with other relevant measures.  Given the richness of the data, it seems like some additional analysis might shed further light on the complex findings.

Response: We thank the reviewer for their suggestion. We clarify that Table 2 assesses the bivariate relationship between each domain of agency and peer-violence among boys and girls in each site, without accounting for adjustment variables. Table 3 is the multivariable table which adjusts for all the variables in the appendix table. Though we have summarized key results from the table in the text, per the suggestion of Reviewer #1, we are hesitant to overinterpret the effects of the adjustment variables. We agree that there is considerable complexity and nuance in the findings, however, given the number of results presented across two sites and by sex, we believe focusing only on the key research question and variables of interest creates a simpler and more compelling paper. However, we appreciate the Reviewer’s suggestion and agree that interactions may be important to consider. Prior to submission we tested for interactions between agency and SES and agency and school status but did not see any interactions in either site among boys and girls. We have included this clarification.

c.      The decision to create tertiles of the three dimensions of agency appears somewhat arbitrary.  Is there a literature to justify this treatment of the data?  Do results look similar if you have a continuous variable or above/below median?  Are these tertiles created within gender/site? If you continue to use tertiles, the data in Table 1 should also be summarized this way. It would be good to know the mean (s.d.) freedom of movement for each tertile etc.

Response: We had initially intended to keep agency scores continuous. However, the distributions were not normaly distributed. We used tertiles to address this issue and to facilitate interpretation of the results across different agency measurements.

We have added a sentence in the method section to justify our use of tertiles (Line 229-231).

“ These measures were not normally distributed and therefore we created tertiles, based on the distribution of the outcome by site and sex.”

d.      Are the differences between boys and girls significant? Are the differences across locations significant?  Given that one site has only school kids and the other has both, does that explain the difference in findings across contexts?

We have included the p-values testing differences between boys and girls in each site in Table 1. We did not include p values for site interaction in the Tables as these differences may also represent differences in survey implementation. Nevertheless, we included a sentence summarizing differences by site and sex in the results. (Line 341-350)

3.      Minor Comments

*       Given that GEAS covers a lot more countries, and now has longitudinal data for some, why limit to these two countries in the cross-section? It also seems like a bid of an odd choice given the very different sampling frames for the two contexts.  Perhaps only focusing on in school adolescents in the two contexts would allow for a clearer comparison? I am sure the authors have their reasons, but this needs at least a footnote to justify the choice.

Response:

- We had initially included more countries in this cross sectional analysis but excluded Shanghai and New Orleans due to the low proportion of adolescents reporting peer-violence in those settings. We also excluded Belgium and Indonesia due to higher levels of missing data for the covariates, leading to the exclusion of 20% of the sample.
- Out of school participants were included in the Kinshasa sample because of higher levels of early school dropout than in other sites (line 40). School status is adjusted for in the analysis. In addition, we found no significant differences in the associations between agency and peer violence among in and out of school adolescent. Finally, when we ran the analysis excluding out of school adolescents from the Kinshasa sample, we found similar differences by site as for the full sample. We have reported these findings in the method section (line 271-273) and in the results section (line 341-350)
- We are unable to present longitudinal data for this research as the longitudinal data for Blantyre was not available at the time of analysis. However, we expect to re-evaluate the relation between agency and violence over time using several rounds of the Kinshasa data and round 2 of the Malawi study.

*    I suggest adding the data from Figure 1 to Table 1, the figure does not add that much value.

Response:

Thank you we have added the data from Figure 1 to Table 1.
