# Supplementary material for: Assessing the relationship between agency and peer violence among adolescents aged 10 to 14 years in Kinshasa, Democratic Republic of Congo and Blantyre, Malawi: A cross-sectional study
Source: PLoS Med. 2021 Dec 13;18(12):e1003552. doi: 10.1371/journal.pmed.1003552 (PMC8716028; doi:10.1371/journal.pmed.1003552)
Supplement: Attachment — Submitted filename: Response to Reviewers - R2.docx [file pmed.1003552.s013.docx]

Dear Editorial Board of PLoS Medicine,

Thank you very much for reading and reviewing our manuscript titled “Assessing the Relationship Between Agency and Peer-violence among Very Young Adolescents in Two African Settings: A Cross-Sectional Study”. We are appreciative of your comments and suggestions and believe the revised manuscript has substantially improved after incorporating your feedback.

In this submission we include a point-by-point explanation of each comment raised by yourself and the reviewers. We have a copy of the manuscript in track changes and in a clean format. We have incorporated all the changes you have requested. The final manuscript has been reviewed and revised by all co-authors.

Requests from the editors:

1. From the Academic Editor: Please address the following comments:

-The measure of victimization/perpetration is based on only 2 questions focused on physical perpetration/victimization. This type of behavior may be gender-specific, other types of peer-violence (e.g. relational) may be gender-specific as well.

– Response: We have added a gender description of respondent/peer dyads and have added a sentence in the limitation section to acknowledge the limitations of our measure

Peer violence mostly involved same sex peers in Kinshasa, for example among adolescents who were victimized, 64% of boys were victimized by boys only and 61% of girls were victimized by girls only. Likewise, among adolescents who perpetrated violence, 60% of boys committed aggression against boys only and 60% of girls committed aggression against girls only. Experiences were more diversified in Blantyre. Among adolescents who were victimized, 38% of boys were victimized by boys only and 46% by girls only and 38% of girls were victimized by girls only and 38% were victimized by boys only. Among adolescents who perpetrated violence, 39% boys committed aggression on boys only and 40% committed aggression against girls only while 44% of girls committed aggression against girls only and 29% perpetrated violence against boys only. (page 14 – 15, line 314 – 323).

We also acknowledge the limitation of our peer-violence measure, which only focuses on physical violence and doesn’t account for gender-based violence behaviors. The small sample sizes prevented more refined analysis of same sex versus opposite sex violence experiences and additional information would be needed to consider other types of peer-violence (verbal, emotional and cyber) which may also vary by gender and context (page 28, line 476 – 480).

- The temporal ordering between outcome (over the prior 6 months) and primary explanatory variables (agency, at the time of survey) is problematic in the context of the cross-sectional nature of the data.

– Response: We acknowledge the cross-sectional nature of the data does not allow us to determine association. We have therefore removed any language which suggests temporality within the results section and included a statement in the limitations (page 28, line 468 – 472)

-The description of agency-related variables is limited and warrants more detail

- Response: We have added information on the development of the agency instruments that were adapted from empowerment constructs for older populations.

We used the GEAS cross-culture measures of agency, that reflect three common dimensions of women’s empowerment (freedom of movement, voice and decision-making power), that are salient in early adolescence. The dimensions were adapted to the developmental stage of early adolescence, when young people have limited autonomy and validated in formative research across 11 settings in five continents (Zimmerman et al. 2019) (page 11, line 237 – 241)

-Sex differences are referred to and it seems as if these should be referred to as gender differences.

-Response: We have changed sex differences to gender differences throughout the manuscript.

-The discussion of the analytical strategy is limited, and it is not clear how the nested nature of the data was handled.

–Response: We have provided additional information regarding analytical strategy used in this paper (page 12 – 13, line 271 – 294).


Other editorial points:

2. Throughout: Sex vs Gender terminology: It seems as if “sex” was established via the GEAS questions and measure (Supporting File 4) question: “Are you a boy/girl” and we suggest changing “sex” to “gender” throughout. Please also see, <https://nam02.safelinks.protection.outlook.com/?url=https%3A%2F%2Fwww.who.int%2Fhealth-topics%2Fgender&amp;data=04%7C01%7Caramaiy1%40jhu.edu%7C26109a3a157c4a7e735208d98f57d899%7C9fa4f438b1e6473b803f86f8aedf0dec%7C0%7C0%7C637698429727072178%7CUnknown%7CTWFpbGZsb3d8eyJWIjoiMC4wLjAwMDAiLCJQIjoiV2luMzIiLCJBTiI6Ik1haWwiLCJXVCI6Mn0%3D%7C1000&amp;sdata=0BcJBZP8bTcZGiM7RcKAb9lLFXyHp3r9EuwzMCOtFQ0%3D&amp;reserved=0>.

– Response: We have followed this reviewer’s suggestion and changed sex to gender throughout the manuscript.

3. Abstract: Background: It might be helpful to include a sentence briefly describing personal agency.

- Response: We expanded on the definition of “power to”:

“Although there is interest in empowering young people to challenge harmful norms, there is scant research on how individual agency, and specifically the “power to” resist or bring about an outcome (Allen 1999) relates to peer violence perpetration and victimization in early adolescence.” (page 2, line 29 – 31)

4. Abstract: Methods and Findings: Please include the percent breakdown by gender for each survey site.

–Response: We have followed this recommendation and added the following information.

“Girls comprised 49.8% of the sample in Kinshasa and 50.7% in Blantyre” (Page 2, line 36 – 37)

5. Abstract: Methods and Findings: Please report exact p values, unless p<0.001

– Response: We have included the exact p-values (Page 2 – 3, line 46 – 60)

6. Abstract: Methods and Findings: Please clarify that T represents tertile at first use of the abbreviation. – Response: We have removed the abbreviation and kept ‘tertile’ throughout the abstract and manuscript (page 2 – 3, line 50 – 60).

7. Abstract: Line 60-61: We suggest revising to “The main limitation of the study is that due to the cross-sectional nature…”

- Response: We have revised as suggested “A main limitation of this study is that the cross-sectional nature of the data does not allow a causal interpretation of the findings, which need further longitudinal exploration to establish temporality. ” (page 3, line 62 – 64)

8. Author summary: Line 85-86: Here, and throughout the manuscript, please avoid language implying causality: “...higher agency resulted in higher likelihood of being in the perpetrator-victim overlap group…”

– Response: We have revised the manuscript to avoid interpretation of causality. For example the sentence was revised as follows “Results show that generally higher agency (greater voice, decision making or freedom of voice) is associated with an increased relative risk of reporting a perpetrator-victim overlap relative to no violence, especially among boys.” (page 5, line 92 – 94)

9. Introduction: Line 135-140: Please clarify the final sentence of this paragraph. You mention that empowerment of women/girls is a mechanism to reduce violence against women and challenge hegemonic forms of masculinity, but in the final sentence mention that empowerment programs could backfire in threatening male hegemony in relationships.

– Response: We have now clarified the sentence that reads “A number of studies support raising girls’ voice and agency to prevent gender based violence (Yount, Krause, and Miedema 2017), but a few studies also alert to the potential backlash associated with challenging the gender order by raising women’s autonomy and reducing male dominance, that has, in some cases, resulted in increased gender based violence (Fakir et al. 2016; Eswaran and Malhotra 2011; Koenig et al. 2003).” (page 7, line 50 – 54)

10. Methods: Line 174-175: Please provide some more detail on how participants were selected, including selection of schools, participants within schools, participants not enrolled in school.

– Response: We have now provided more detail about how schools and participants were selected for both sites (page 9 – 10, line 192 - 209):

“In Kinshasa, in school and out of school adolescents were selected form two urban poor communes selected by Save the Children for an intervention (Growing Up Great!). Schools were purposively selected in the intervention and control zones after stratification by commune and school type (public school, religiously-affiliated non for profit schools and private religious schools). In the intervention arm, 25 students per school were selected from the Growing Up Great! school clubs. In the control arm, 25 adolescents were randomly selected after stratification by sex and age . Out of school adolescents were recruited from the same communes and randomly selected from a listing of households. A total of 2840 adolescents (2016 in school and 826 out of school) were selected and provided assent and parental consent to participate. All participants who were approached completed the survey.

In Blantyre, sampling was a two-stage process with the selection of schools and students for an intervention (Very Young Adolescence 2.0), which was ultimately cancelled due to the COVID-19 pandemic. Eight public urban schools which offered 6th grade were listed and purposively allocated to ‘intervention’ or ‘control’ status in consultation with the city education authorities and head teachers. Class registers for 6^th^ grade in each of the schools were used to select between 200 and 550 adolescents per school depending on number of students at each school. The sample for the present analysis includes 1,694 participants, out of the 2,140 students who had been approached before the COVID-19 shutdown.”

11. Methods: Line 199-200: Please note that this was parental consent and adolescent assent.

– Response: We have revised the sentence “adolescents aged 10-14 years who provided adolescent assent and parental consent to participate and who were able to understand the questionnaire” were eligible to participate (page 9, line 189 – 190)

12. Methods: Line 228: Wealth quintile is reported in the S5 and S6 Tables, but the assessment of wealth is not described in the Methods.

–Response: We added a description of wealth asset (quintile) in the method section (page 12, line 263) and updated S6_Table (page 42)

13. Methods: Line 259-267: Please consider if the description of your study hypotheses would be better presented in the last paragraph of the Introduction.

–Response: We have moved the hypotheses to the introduction as suggested (page 8, line 174 – 179)

14. Results: Please report the numbers of participants at each site, and proportions by gender. present numerators and denominators for percentages.

– Response: We have included these details in the first sentence of the result section: “A total of 2,540 adolescents were included in Kinshasa; evenly distributed between girls (49.8% (n=1266)) and boys (50.2% (n=1274)). A total of 1,213 adolescents responded in Blantyre with 50.7% of girls (n=615) and 49.3% of boys (n=598)” (page 14, line 296 – 299)

15. Results: Line 281: Please clarify if this should be boys/men and girls/women.

– Response: We have clarified the sentence “boys as tough and girls as vulnerable” (page 14, line 306 – line 307).

16. Results: The table shows the overall difference for male vs female in terms of victimization/perpetration but comparisons for perpetration alone or victimization alone, for example, are not shown. Please present these: “Sex-differences in perpetration or victimization alone were negligible.”

– Response: We have added p-values to the text but not shown it on the table since all the other Chi squares are for the overall group rather than individual category differences: “Kinshasa perpetration: p=0.1, Kinshasa victimization: p=0.2, Blantyre perpetration p=0.3, Blantyre victimization p=0.3” (page 14, line 313 – 314)

17. Results: Line 286-290: Please present the results on sex differences in perpetration/victimization (same sex/opposite sex) in a table (such as the one presented in the response to reviewer comments).

– Response: We have added these results in Table 1 and updated the text in the results (page 14 – 15, line 314 – 323):

“Peer violence mostly involved same sex peers in Kinshasa, for example among adolescents who were victimized, 64% of boys were victimized by boys only and 61% of girls were victimized by girls only. Likewise, among adolescents who perpetrated violence, 60% of boys committed aggression against boys only and 60% of girls committed aggression against girls only. Experiences were more diversified in Blantyre. Among adolescents who were victimized, 38% of boys were victimized by boys only and 46% by girls only and 38% of girls were victimized by girls only and 38% were victimized by boys only. Among adolescents who perpetrated violence, 39% boys committed aggression on boys only and 40% committed aggression against girls only while 44% of girls committed aggression against girls only and 29% perpetrated violence against boys only.”

18. Results: Line 299-311: Please clarify if the p values reported represent the overall chi-square test (including all three tertiles) or if the results reported reflect comparisons between T1 and T3 specifically. Please describe this analysis more completely in the methods, including whether correction for multiple testing was applied.

–Rseponse: We have revised the method section that now reads “We first described the distribution of the individual, family, peer and community characteristics of the study population by gender and site and tested for gender differences using Chi-square tests or unpaired t-tests (in the case of gender norm perceptions and agency scores). (page 13, line 272 – 274)

19. Results: Line 334-343: Please present the complete results (this can be in a table) for tests done to examine the main effect of site and interactions:

Response: We have now shown the complete results for the site and agency interactions in S5_table and expanded on the text:

“While interpretation is limited given methodological differences by site, we also note significant differences in the associations between freedom of movement and peer violence that are stronger among girls in Blantyre compared to girls in Kinshasa girls (S5_table) ), which remained even after excluding out of school adolescent in Kinshasa (S6_table). No site interactions were noted for boys. ” (page 22 – 23, line 371 – 375 and S5_table)

20. Results: Line 342-343: Please present the results of the analyses with out of school students excluded: “These differences remained after excluding out of school adolescent in Kinshasa.”:

Response: We have shown the table (S6_table) and updated this sentence:

“While interpretation is limited given methodological differences by site, we also note significant differences in the associations between freedom of movement and peer violence that are stronger among girls in Blantyre compared to girls in Kinshasa girls (S5_table) ), which remained even after excluding out of school adolescent in Kinshasa (S6_table). No site interactions were noted for boys. ” (page 22 – 23, line 371 – 375 and S5_table).” ((page 22 – 23, line 371 – 375 and S6_table)

21. Results: Line 344-361: Please quantify the results in the text, if possible. Please avoid the use of language implying causality in this section (e.g. “...high parental connectedness relative to low connectedness increased the RRR…”

Response: We have revised this paragraph to include results in the text (page 23 – 24, line 377 – 411):

“Other factors related to peer violence in the multivariate analyses are presented in S7_Table and S8_Table. In both sites, older boys were less likely to report being victimized compared to younger boys (In Kinshasa: RRR = 0.6 (0.4 -0.9), p=0.03; in Blantyre RRR= 0.4 (0.2 - 0.7), p<0.001)). Likewise, older boys in Kinshasa were less likely to report perpetration/victimization overlap than younger boys (RRR =0.6 (0.4-0.8), p=0.001). Boys who were lower than their age expected grade were less likely to be victimized compared to out of school boys in Kinshasa (RRR=0.5 (0.2-0.9), p=0.03), and boys in their age expected grade or higher were less likely to be victimized than boys in lower than age expected grades in Blantyre (RRR=0.5 (0.3-0.97), p=0.04). In Kinshasa, greater wealth was associated with a lower risk of victimization (wealth *quintile 2 versus 1*: RRR=0.6 (0.3-0.99), p=0.05) and a lower risk of perpetration/victimization overlap (wealth *quintile 2 versus 1*: RRR=0.5 (0.3-0.8), p=0.01; wealth *quintile 4 versus 1*: RRR=0.4 (0.3- 0.8), p=0.004) compared to adolescents living in the poorest households. In both sites, adolescents who reported a history of two or more ACEs had elevated risk of all forms of peer violence experiences relative to those with no history of ACEs. Parent and peer-relations, social cohesion and social norms were related to peer violence experiences although these associations differed by site. Among Kinshasa boys, being close to a parent was associated with an elevated relative risk of victimization (RRR=1.6 (1.04- 2.4), p=0.03) and a lower risk of perpetration (RRR=0.7 (0.5-0.99), p=0.05) and of perpetration & victimization overlap (RRR=0.6 (0.4-0.8), p<0.001) compared to boys who were not close to their parents. Likewise, girls in Kinshasa who were close to their parents had a lower relative risk of violence perpetration (RRR=0.5 (0.4-0.8), p=0.001) and of perpetration/ victimization overlap (RRR=0.5 (0.3-0.8), p=0.002) compared to girls who were not close to their parents . In addition, they were less likely to be victimized (RRR=0.5 (0.3- 0.8), p=0.002). In Blantyre, boys with opposite sex friends had an elevated relative risk of perpetration relative to those with same sex friends (RRR=3.9 (1.3-12.1), p=0.02) while girls with opposite sex friends had an elevated relative risk of perpetration/victimization overlap (RRR=2.1 (1.0 t- 4.3), p=0.04). In Kinshasa, high social cohesion was associated with a lower relative risk of perpetration among boys (RRR=0.8 (95% CI: 0.4 to 0.9), p=0.02); however, among girls, it was associated with an elevated relative risk of perpetration (RRR=2.0 (1.3 -3.0), p=0.001) and of combined perpetration/victimization (RRR=2.1 (95% CI: 1.3 to 3.3), p=0.003). In Blantyre, high social cohesion among boys was associated with an elevated relative risk of victimization (RRR=1.7 (1.03 -2.8), p=0.04) and of a victimization & perpetration overlap (RRR=3.2 (1.8 -5.7), p<0.001). Finally, higher perceptions of gender stereotypical traits was associated with an elevated relative risk of a victimization & perpetration overlap among girls in Kinshasa (RRR=1.7 1.1 to 2.6), p=0.01). ”
22. Discussion: Line 440: Please expand on the statement that social desirability bias may have factored in to responses from Kinshasa.

Response: We have expanded this statement to include:

On the other hand, social desirability bias may have impacted responses in Kinshasa which were collected face-to-face (Grimm 2010). We note, however, that violence perpetration was equally prevalent in Blantyre and Kinshasa, which may signal low social desirability bias (page 29, line 491 - 493)

23. Table 1: Please note in the legend the statistical tests use to investigate sex differences.

Response: We have revised the note as follows “Chi-square tested were used to estimate gender difference within each site for all variables except gender norm perceptions and agency, which used unpaired t-test. (Legend under table 1)

24. Table 2: Please adjust the formatting of the table, as the column widths lead to the text being difficult to read. Please note in the legend the statistical tests used.

Response: We have changed the layout to landscape to make the text easier to read (page 19 – 21). We have also included in the legend “Chi-squared tests were used to assess differences in peer-violence experiences by agency tertiles” (Legend under table 2)

25. Table S5 and S6: Instead of using gray boxes, we suggest using different symbols with footnotes to describe the specific reasons why data were unavailable for the analyses.

Response: – We have now included letters and added footnotes on why data were unavailable for the analyses presented in Table S7 and S8.

26. STROBE checklist: Thank you for including the checklist. Please use section and paragraph numbers to identify locations within the text, rather than page numbers.

Response: We have included section and paragraph numbers in the checklist.


Comments from the reviewers:

Reviewer #1: Overall, this revision is very responsive to my previous comments. A few issues regarding clarity remain:

In my earlier comments, I was not clear enough about what I meant by "clarifying the Parameters of interest". In the statistical methods section, I recommend that the authors tell the readers the parameter, or estimand, that they will be using and how to interpret it. For example, the main results in table 3 consist of the "relative risk ratio", which, not being a Stata user myself, is an unfamiliar term to me. This measure ought to be defined in the statistical methods section and interpreted for the readers (beyond stating that the relative risk ratio is a measure that comes out of such and such model). What does it mean? If standard multinomial logistic regression is used, this is the odds ratio for being the dependent variable being in the given category rather than the reference category for a one-unit change in the independent variable.

– Response: Thank you. We have clarified this in the method section: The models produce adjusted Relative Risk Ratios (RRR), which indicate how the risk of the outcome falling in one if the comparison groups (victimization, perpetration or victimization/perpetration overlap) compared to the risk of being in the reference group (no violence) changes with one unit change of the independent variable (agency tertiles), after adjusting for other covariates (UCLA institute for digital research and education n.d.).” (Page 13, line 282 – 286)

Thank you very much for reviewing our manuscript.

Sincerely,

Astha Ramaiya, DrPH
