# Supplementary material for: Assessing the relationship between agency and peer violence among adolescents aged 10 to 14 years in Kinshasa, Democratic Republic of Congo and Blantyre, Malawi: A cross-sectional study
Source: PLoS Med. 2021 Dec 13;18(12):e1003552. doi: 10.1371/journal.pmed.1003552 (PMC8716028; doi:10.1371/journal.pmed.1003552)
Supplement: Attachment — Submitted filename: Response to Reviewers - R3.docx [file pmed.1003552.s014.docx]

Dear Editorial Board of PLoS Medicine,

Thank you very much for reading and conditionally accepting our manuscript titled “*Assessing the relationship between agency and peer-violence among adolescents aged 10-14 years in Kinshasa, Democratic Republic of Congo and Blantyre, Malawi: A cross-sectional study*”. We are appreciative of your comments and suggestions and believe the manuscript has substantially improved after incorporating your feedback.

In this submission we include a point-by-point explanation of each comment raised by the editors. We have a copy of the manuscript in track changes and in a clean format. We have incorporated all the changes you have requested. The final manuscript has been reviewed and revised by all co-authors.

1. Title: Please revise to include the age range and please use sentence-case capitalization: “Assessing the relationship between agency and peer-violence among adolescents aged 10-14 years in Kinshasa, Democratic Republic of Congo and Blantyre, Malawi: A Cross-Sectional Study”: We have changed the title to sentence-case capitalization and changed the title to what you have proposed (Page 1, line 1 – 3)

2. Throughout: Please replace “sex” with “gender” where appropriate, including in the Tables. – We have replaced sex with gender throughout the text and within the tables.

3. Abstract: Line 44: Please note that covariates adjusted for include individual, family, peer, and community factors. - We have edited the abstract to say “Univariate analysis and multivariable multinomial logistic regressions were conducted to evaluate the relationships between each agency indicator and peer violence. The multivariable regression adjusted for individual, family, peer and community level covariates. ” (page 2, line 54 – 55).


4. Author summary: Line 101: We suggest revising to: “both genders” in this point. We have changed sex to gender (page 5, line 115)

5. Author summary: Line 107: Please fully spell out the abbreviation “ACEs” at first use in the text. – We have spelled out ACEs as Adverse Childhood Experiences (page 5, line 121).

6. Introduction: Line 132: Please clarify the direction of relationship between social skills and risk of violence in this sentence. – We have now edited the sentence to say “At the individual level, age, gender, history of adverse child experiences (ACEs), medical/physical conditions which affect the nervous system, psychological characteristic i.e. impulsivity, attention deficit and substance abuse are associated with increased risk of violence, including bullying and general aggression.” (page 6, line 147 - 149).

7. Methods: Line 234, 264: Please change “sex” to “gender” in these sentences. We have changed sex to gender throughout the document.

8. Results and Tables: Please report p values to two decimal places if p>0.01, three decimal places if p>0.001, and for smaller values please report p<0.001. We have amended and added the p-values for all the tables and results based on your guidance.

9. Results: Line 315 and 400-401: Please change “sex” to “gender” if accurate. – we have changed sex to gender throughout the manuscript.

10. Results: Line 381-382: Please clarify if this should be “Boys who were in a grade lower than their age-expected grade…” – We have clarified this and now say “Boys who were in a grade lower than age-expected grade were less likely to be victimized compared to out of school boys in Kinshasa (RRR=0.5 (0.2-0.9), p=0.03), and boys who were in their age expected grade or higher were less likely to be victimized than boys who were in a grade lower than age-expected grade in Blantyre (RRR=0.5 (0.3-0.97), p=0.04).” (page 23, line 439 – 443)

11. Discussion: Line 478: Please change “sex” to “gender” in this sentence. – We have changed sex to gender throughout the manuscript.

12. Discussion: Line 503: We suggest revising to: “...raising the possibility that gender socialization may expose boys to more interpersonal violence as they enjoy more freedom of movement…” or similar. – We have revised this sentence to “Our results have important programmatic implications, raising the possibility that gender socialization may expose boys to more interpersonal violence as they enjoy more freedom of movement and are expected to protect themselves.” (page 29, line 626)

13. References: Please check each citation for completeness and accuracy. For example, please provide the complete information for Reference 7. For guidance, please use to the "Vancouver" style for reference formatting, and see our website for other reference guidelines <https://nam02.safelinks.protection.outlook.com/?url=https%3A%2F%2Fjournals.plos.org%2Fplosmedicine%2Fs%2Fsubmission-guidelines%23loc-references&;data=04%7C01%7Caramaiy1%40jhu.edu%7C33e527899fea4360448308d9aac2c56c%7C9fa4f438b1e6473b803f86f8aedf0dec%7C0%7C0%7C637728575792149996%7CUnknown%7CTWFpbGZsb3d8eyJWIjoiMC4wLjAwMDAiLCJQIjoiV2luMzIiLCJBTiI6Ik1haWwiLCJXVCI6Mn0%3D%7C3000&sdata=63ajJlUxWK%2BtFpilt%2FiZBZSG63EFLQipKz30z9iXZXQ%3D&reserved=0> – We have rechecked the references and made the necessary changes.

14. Table 1: Please revise “Peer sex composition” and “Any opposite sex friends” to use “gender” if accurate to report this way. – We have changed peer sex composition to peer composition and any opposite sex to opposite gender throughout the manuscript.

15. Table 1: Only one category of “Time spent with friends” is reported, though there are 5 categories for this question, please note for this category that responses were collapsed (e.g. please clarify this in the table legend, and in the Methods at line 265). Please also clarify this for “parent connectedness” and any other questions for which responses were collapsed/dichotomized. – We have included a note in the legend of Table 1 and clarified the dichotomization in the methods section (page 17, line 370 – 371; page 12, line 280 – 288)

16. STROBE Checklist: Please revise the paragraph, using section and paragraph numbers to refer to locations in the text. Please do not refer to page numbers (e.g. Methods, paragraph 2). For “Funding” please refer to the “Financial Disclosures” section. – We have updated the STROBE checklist.

17. Supplement 9: Thank you for including the data underlying the study as a supporting information file. Please note that we do not require that authors submit their entire data set if only a portion of the data was used in the reported study. The included file contains a large number of variables. A file explaining the nature of each variable would be help researchers to use the dataset.

Prior to sharing human research participant data, authors should consult with an ethics committee to ensure data are shared in accordance with participant consent and all applicable local laws.
Data sharing should never compromise participant privacy. It is therefore not appropriate to publicly share personally identifiable data on human research participants. The following are examples of data that should not be shared: Name, initials, physical address; Internet protocol (IP) address; Specific dates (birth dates, death dates, examination dates, etc.); Contact information such as phone number or email address; Location data.

Data that are not directly identifying may also be inappropriate to share, as in combination they can become identifying. For example, data collected from a small group of participants, vulnerable populations, or private groups should not be shared if they involve indirect identifiers (such as sex, ethnicity, location, etc.) that may risk the identification of study participants.
Steps necessary to protect privacy may include de-identifying data, adding noise, or blocking portions of the database.

Please check the file and remove any potentially identifying information “e.g. deviceid, subscriberid, simid, devicephonenumber, etc.

Thank you we have now uploaded a new dataset with just the analytical variables (Supplement 9). We have also included a copy of the codebook (Supplement 10) which outline the variable name and response option labels.

18. Supplement 2: Kinshasa IRB: Please confirm that the appropriate usage rights apply to the use of the maps on page 3 and 4 of the protocol. PLOS applies the Creative Commons Attribution (CC BY) license to all the works we publish, and the use of figures must be compatible with this license. Please see our guidelines for map images: <https://nam02.safelinks.protection.outlook.com/?url=https%3A%2F%2Fjournals.plos.org%2Fplosmedicine%2Fs%2Ffigures%23loc-maps&;data=04%7C01%7Caramaiy1%40jhu.edu%7C33e527899fea4360448308d9aac2c56c%7C9fa4f438b1e6473b803f86f8aedf0dec%7C0%7C0%7C637728575792149996%7CUnknown%7CTWFpbGZsb3d8eyJWIjoiMC4wLjAwMDAiLCJQIjoiV2luMzIiLCJBTiI6Ik1haWwiLCJXVCI6Mn0%3D%7C3000&sdata=wV2cACcFv1ZUa6bxr3%2BSS8Qf0VkHKHbVRgbrZsXmH4s%3D&reserved=0>

Please note relevant permissions and license/copyright information in the legends.

We have now removed the maps within the Kinshasa IRB to comply with the CC BY license.

Thank you very much for reviewing our manuscript.

Sincerely,

Astha Ramaiya, DrPH
